# Epithelial WNT secretion drives niche escape of developing gastric cancer

**DOI:** 10.1186/s12943-025-02543-z

**Published:** 2025-12-16

**Authors:** Jaehun Lee, Soomin Kim, Youngchul Oh, Stephan R. Jahn, Jihoon Kim, Yeongjun Kim, Tim Schmäche, Sang-Min Kim, Isaree Teriyapirom, Thomas Groß, Ohbin Kwon, Jungmin Kim, Somi Kim, Anne-Marlen Ada, Andrea Català-Bordes, Youngwon Cho, Jinho Kim, Amanda Andersson-Rolf, Sebastian R. Merker, Joo Yeon Lim, Ji-Yeon Park, Thomas M. Klompstra, Ki-Jun Yoon, Dae-Sik Lim, Ho-Seok Lee, Jong Kyoung Kim, Eunyoung Choi, James R. Goldenring, Jae-Ho Cheong, Hyunki Kim, Daniel E. Stange, Heetak Lee, Bon-Kyoung Koo, Ji-Hyun Lee

**Affiliations:** 1 Center for Genome Engineering, Institute for Basic Sciences, Daejeon, Republic of Korea; 2 Department of Life Sciences, Pohang University of Science and Technology (POSTECH), Pohang, Republic of Korea; 3 Department of Biology, Kyung Hee University, Seoul, Republic of Korea; 4 Department of Visceral, Thoracic and Vascular Surgery, Medical Faculty and University Hospital Carl Gustav Carus, Technische Universität Dresden, Dresden, Germany; 5 Department of Medical and Biological Sciences, The Catholic University of Korea, Bucheon, Republic of Korea; 6 National Center for Tumor Diseases Dresden(NCT/UCC), a Partnership Between DKFZ, Faculty of Medicine and University Hospital Carl Gustav Carusm, Technische Universität Dresden, and Helmholtz-Zentrum Dresden-Rossendorf (HZDR), Dresden, Germany; 7 Department of Pathology, Yonsei University College of Medicine, Seoul, Republic of Korea; 8 Institute of Molecular Biotechnology of the Austrian Academy of Sciences (IMBA), Vienna BioCenter (VBC), Vienna, Austria; 9 Vienna BioCenter PhD Program, Doctoral School of the University of Vienna and Medical University of Vienna, Vienna, Austria; 10 Core Unit for Molecular Tumor Diagnostics (CMTD), National Center for Tumor Diseases (NCT), NCT/UCC Dresden, a Partnership Between DKFZ, Faculty of Medicine and University Hospital Carl Gustav Carus, TUD Dresden University of Technology, and Helmholtz-Zentrum Dresden-Rossendorf (HZDR), Dresden, Germany; 11 Graduate School of Stem Cell and Regenerative Biology, KAIST, Daejeon, 34141 Republic of Korea; 12 Department of Surgery, Yonsei University College of Medicine, Seoul, Republic of Korea; 13 Severance Biomedical Science Institute, Yonsei University College of Medicine, Seoul, 03722 Republic of Korea; 14 Department of Medical Science, Graduate School of Medical Science, Brain Korea 21 Project, Yonsei University College of Medicine, 50 Yonsei-Ro, Seodaemun-Gu, Seoul, 03722 Republic of Korea; 15 Section of Surgical Sciences, Vanderbilt University Medical Center, Nashville, TN USA; 16 Epithelial Biology Center, Vanderbilt University Medical Center, Nashville, TN USA; 17 Precision Medicine Center, Future Innovation Research Division, Seoul National University Bundang Hospital (SNUBH), Seongnam, Gyeonggi-do 13620 Republic of Korea; 18 Department of Genomic Medicine, Seoul National University Bundang Hospital, Seongnam, Gyeonggi-do 13620 Republic of Korea; 19 Department of Laboratory Medicine, Seoul National University Bundang Hospital, Seoul National University College of Medicine, Seongnam, Gyeonggi-do 13620 Republic of Korea; 20 Gradiant Bioconvergence Inc., Seoul, Republic of Korea; 21 Department of Biological Sciences, Korea Advanced Institute of Science and Technology (KAIST), Daejeon, Republic of Korea; 22 KAIST Stem Cell Center, KAIST, Daejeon, 34141 Republic of Korea; 23 Department of Cell and Developmental Biology, Vanderbilt University, Nashville, TN USA; 24 Nashville VA Medical Center, Nashville, TN USA; 25 Department of Biochemistry and Molecular Biology, Yonsei University College of Medicine, Seoul, Republic of Korea; 26 Chronic Intractable Disease for Systems Medicine Research Center, Yonsei University College of Medicine, Seoul, Republic of Korea; 27 Department of Biomedical Systems Informatics, Yonsei University College of Medicine, Seoul, Republic of Korea; 28 German Cancer Consortium (DKTK), Partner Site Dresden, and German Cancer Research Center (DKFZ), Dresden, Germany; 29 Department of Biological Sciences, Sungkyunkwan University, Suwon, 16419 Republic of Korea

**Keywords:** Gastric cancer, Tumor microenvironment, WNT self-sufficiency, KRAS–MAPK–WNT7B axis

## Abstract

**Background:**

WNT signaling plays a key role in maintaining the gastric epithelium and promoting tumorigenesis. However, how gastric tumors achieve WNT niche independence remains unclear, as mutations on *APC* or *CTNNB1*—common mechanisms of ligand-independent WNT activation in colorectal cancer—are infrequent in gastric cancer. Understanding how WNT self-sufficiency is acquired in the stomach is therefore critical.

**Methods:**

We analyzed mouse gastric organoids harboring oncogenic *KRAS*^*G12D*^ with or without *RNF43/ZNRF3* (RZ) or *CDH1/TP53* (CP) mutations, along with corresponding in vivo mouse models. Niche independence was assessed through growth factor withdrawal, Porcupine and pathway-specific inhibitor treatments, and WNT rescue assays. We performed single-nucleus multiome sequencing (RNA + ATAC) to investigate transcriptional and chromatin dynamics. Findings from mouse models were validated using patient-derived gastric cancer organoids, and pan-cancer cell line datasets were analyzed to evaluate clinical and cross-tissue relevance.

**Results:**

Gastric fibroblasts secreted canonical WNT2B to maintain the homeostatic gastric epithelium. Upon *KRAS* activation, epithelial cells were reprogrammed to secrete WNT ligands independently of additional mutations. Single-nucleus multiome analysis revealed that KRAS-driven MAPK signaling opened SMAD2/3-bound enhancers at the *WNT7B* locus, leading to the emergence of *WNT7B*-expressing subpopulations. Inhibition of SMAD2/3 phosphorylation suppressed both organoid growth and *WNT7B* transcription, whereas exogenous WNT restored organoid proliferation. Patient-derived organoids with *HER2* amplification, *KRAS* amplification, or *WNT2* copy-number gain exhibited Porcupine inhibitor-sensitive growth, indicating dependence on WNT secretion from the organoids. Analysis of public transcriptomic datasets further demonstrated that the KRAS–MAPK–WNT7B axis is conserved across other cancer types, including lung cancer.

**Conclusions:**

Gastric tumors can bypass niche dependence by acquiring KRAS–MAPK–SMAD2/3-driven epithelial WNT secretion. Targeting this axis—through MAPK inhibition, SMAD2/3 blockade, or suppression of WNT secretion—may represent a therapeutic vulnerability in gastric cancer and other KRAS-high malignancies.

**Supplementary Information:**

The online version contains supplementary material available at 10.1186/s12943-025-02543-z.

## Introduction

In both humans and mice, the gastric corpus epithelium is continuously regenerated. It invaginates from the lumen to form glands, which are divided into four parts from top to bottom: pit, isthmus, neck, and base. The murine gastric gland is maintained by two distinct stem cell populations—one situated in the isthmus and the other in the base [1–5]. Isthmus stem cells (IsthSCs) are rapidly cycling and maintain the upper region of the gland [4, 6, 7]. These cells are characterized by proliferation markers such as Ki67 and STMN1. On the other hand, basal stem cells (BSCs) are slow-cycling in homeostasis and are marked by WNT-signaling markers TNFRSF19 (TROY) and GPR49 (LGR5). BSCs are a subpopulation of gastric chief cells that function as reserve stem cells upon injury [1, 8, 9], while also being a source of gastric cancer [9–13].

The WNT signaling pathway plays a pivotal role in the maintenance of the gastrointestinal epithelium. For example, Paneth cells in small intestinal crypts secrete WNT3, driving self-renewal of neighboring stem cells and thus tissue maintenance [14]. Mesenchymal cells beneath the epithelium of intestinal crypts are another source of WNTs by secreting WNT2B, next to R-spondin 3 (RSPO3), a WNT signaling enhancer that binds to LGR4/5 expressed by intestinal stem cells [15–18].

Similar to the intestine, mesenchymal cells of the stomach niche play an important role in regulating stem cell function in the stomach by secreting RSPO3 [19–22]. This results in the expression of WNT target genes such as *AXIN2*, *LGR5*, and *TROY* in BSC at the gastric gland base. In line with this, gastric adult stem cell-based organoid cultures require canonical WNT ligands such as WNT3A [1, 8]. Furthermore, recent studies provided evidence that, in addition to RSPO3, WNT ligands are also secreted by sub-glandular mesenchymal cells to support stem cells, though their specific identity in the stomach is not fully elucidated [19, 23].

A full understanding of the source and identity of WNT signals in the stomach is of major interest, as WNT signaling overactivation, driving independence from a WNT niche, is often implicated in various epithelial cancers [24–28]. Among them, gastric cancer is highly prevalent with a high mortality rate [29]. APC mutations are one of the most well-defined ligand-independent modes of constitutive activation of canonical WNT signaling in the intestine, freeing intestinal stem cells from the restricted niche created by WNT and RSPO gradients and allowing the continuous growth of these cells outside of the niche.

The acquisition of niche independence is a key first step in intestinal and colonic tumorigenesis. In gastric tissue, loss of **R**NF43 and/or **Z**NRF3 (RZ)—negative regulators of Frizzled receptors that bind WNT ligands—leads to independence from the WNT enhancer RSPO [30]. Alternatively, RSPO independence can also result from the combined loss of E-**c**adherin and T**P**53 (CP) [31]. However, while APC mutations lead to a complete WNT independence, RSPO independence via RZ or CP loss still depends on WNT ligands to activate the pathway.

Here, we first used single-nucleus profiling and gastric organoid culture to identify the specific WNT ligands that are responsible for the maintenance of the gastric epithelium. We then uncovered two independent mechanisms that enable gastric epithelial stem cells to become fully WNT-independent: Kirsten rat sarcoma viral oncogene homologue (KRAS)-mitogen-activated protein kinase (MAPK) activation by *WNT* gene amplification. Both mechanisms converge on epithelial WNT secretion and allow tumorigenic niche escape. Our results show that, unlike colon cancer, WNT self-sufficiency in gastric cancer is established through different mechanisms that still require the secretion and binding of WNT ligands. This reveals a potential vulnerability of gastric cancer to WNT secretion blockers.

## Results and discussion

### WNT2B is the canonical WNT ligand secreted by gastric mesenchyme to maintain the homeostatic gastric epithelium

**Fig. 1:**
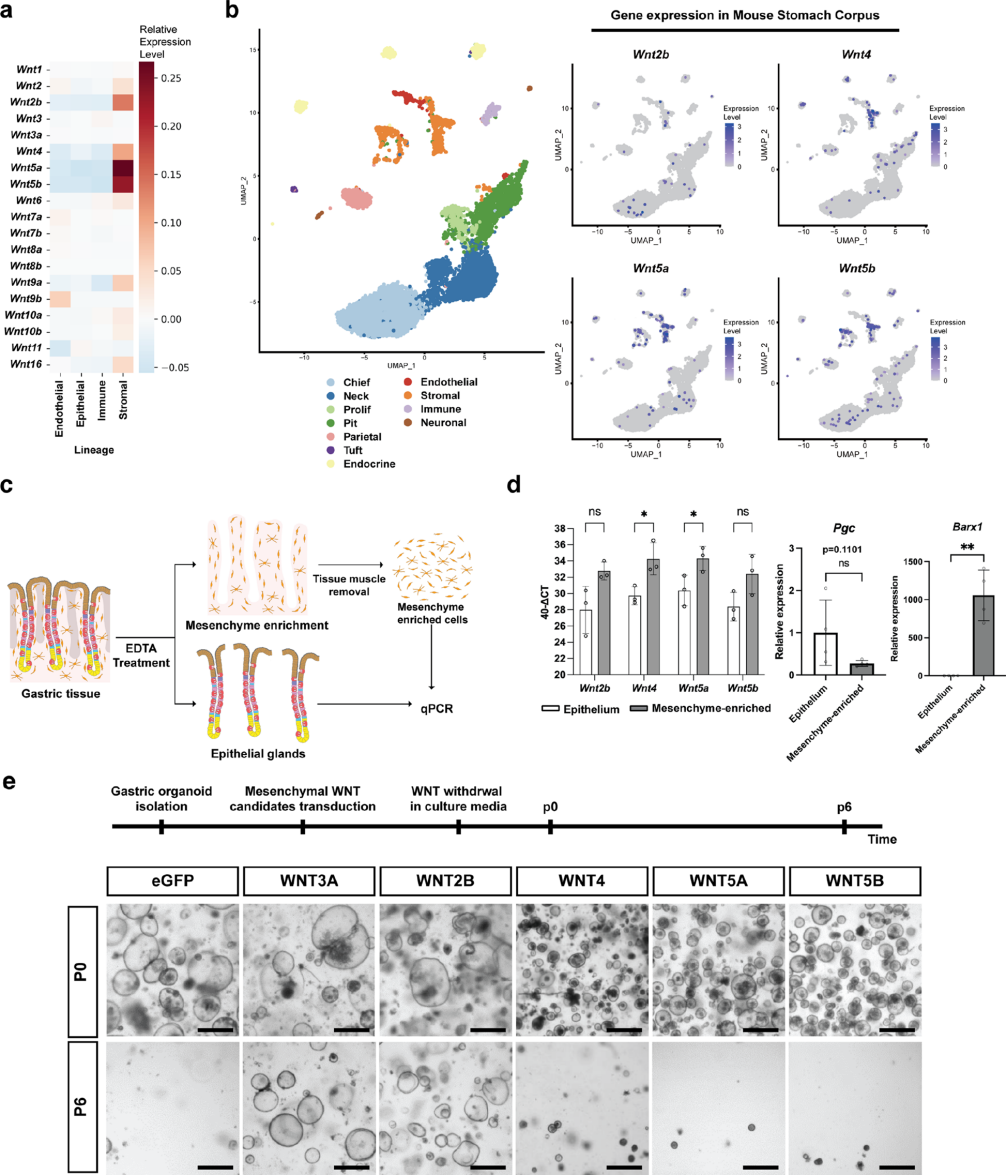
WNT2B is the canonical WNT ligand secreted by the gastric mesenchyme to maintain the normal gastric epithelium. **a** Data from Zhang et al*.* (2024) were used to analyze the relative expression *Wnt* genes in mouse gastric tissue [32]. **b** Left: UMAP of WT mouse gastric corpus tissue sn multiome data. Right: Expression patterns *of Wnt2b, Wnt4, Wnt5a,* and *Wnt5b* projected onto the same UMAP. **c** Scheme for qRT-PCR analysis of gastric tissue following the separation of the epithelial glands from the mesenchymal component. **d** Left: Bar graph showing the expression of different *Wnt* ligands in the epithelial cell and mesenchyme-enriched populations (y-axis was calculated using 40—Δ C_T_). Error bars represent the standard deviation (SD). Statistical significance was determined by unpaired t-test. *, *p* < 0.05; ns, non-significant. Right: qRT-PCR results using *Pgc*, a known epithelial cell marker, and *Barx1*, a known mesenchymal cell marker, in epithelial and mesenchyme-enriched populations. Error bars represent SD. Statistical significances was determined by paired t-test. **, *p* < 0.005; n.s., non-significant. **e** Schematic timeline and representative organoid images of the WNT retrieval assay. Gastric organoids were established from *Rosa26-Cre*^*ERT2*^ mice and subsequently transduced with retrovirus to overexpress a panel of candidate *WNT* genes. Following tamoxifen treatment, WNT-conditioned medium was removed, and organoid growth was observed over six passages. P0: passage 0; P6: passage 6. Scale bar: 1000 μm

To identify the source of canonical WNT ligands that maintain the gastric epithelium, we first examined publicly available single-nucleus RNA sequencing (snRNA-seq) data [32], as well as our own single-nucleus (sn) multiome dataset, which includes snRNA-seq and single-nucleus assay for transposase-accessible chromatin with sequencing (snATAC-seq). Across the whole stomach, *Wnt2b*, *Wnt4*, *Wnt5a*, and *Wnt5b* were the major *Wnt* genes, expressed at higher levels in stromal cells than in other cell types (Fig. 1a and Supplementary Fig. 1a). In our sn multiome dataset of the gastric corpus (Fig. 1b and Supplementary Fig. 1b), enrichment of these four *Wnt* genes in stromal populations was independently confirmed, although a small fraction of epithelial cells also expressed them (Fig. 1b and Supplementary Fig. 1b). Among stromal populations, *Wnt2b* and *Wnt4* were enriched in *Robo2*-high fibroblasts and smooth muscle cells, whereas *Wnt5a* and *WNT5b* were broadly expressed across both *Robo2*-high and *Robo2*-low fibroblasts, as well as smooth muscle cells (Supplementary Fig. 1c, d) [33]. We found little to no enrichment of other WNT ligands specifically in stromal cell types of the gastric corpus (Fig. 1a, and Supplementary Fig. 1a, e). Next, we confirmed WNT ligand expression in the mesenchyme by performing quantitative real-time polymerase chain reaction (qRT-PCR) on freshly isolated epithelial and mesenchymal compartments of gastric tissue (Fig. 1c). Successful enrichment of both compartments was confirmed by the expression of the epithelial marker *Pgc* and the mesenchymal marker *Barx1*, with minimal epithelial contamination in the mesenchyme-enriched population (Fig. 1d). Consistent with the previous datasets (Fig. 1a, b and Supplementary Fig. 1), the mesenchyme-enriched population expressed *Wnt2b*, *Wnt4*, *Wnt5a*, and *Wnt5b* (Fig. 1d), whereas *Wnt* gene expression was barely detectable in the epithelial population (Supplementary Fig. 2a). These data indicate that WNT ligands are mainly secreted from gastric mesenchymal cells.

Next, we used gastric organoids to functionally test the identified mesenchymal WNT ligands—WNT2B, WNT4, WNT5A, and WNT5B—for their ability to maintain the gastric epithelial stem cells in vitro. We individually overexpressed each candidate ligand in gastric corpus organoids derived from *Rosa26*-*Cre*^*ERT2*^ mice using a CRE-inducible retroviral vector for conditional overexpression (Supplementary Fig. 2b, c) [34]. WNT3A, a standard component of organoid medium, served as a positive control, while enhanced green fluorescent protein (eGFP) served as a negative control (Fig. 1e). After 4-hydroxytamoxifen (4-OHT) treatment to induce overexpression, we cultured the transduced organoids in WNT-deficient medium to test whether any of the overexpressed WNT candidates could substitute for the WNT3A medium supplement (Fig. 1e). Overexpression of WNT2B, as well as the WNT3A, supported gastric organoid growth in the absence of an external WNT source, demonstrating that these WNT ligands can activate the canonical WNT pathway that is essential for epithelial stem cell maintenance. In contrast, the remaining mesenchymal ligands—WNT4, WNT5A, and WNT5B—were unable to support organoid growth, similar to the eGFP negative control. Taken together, these data show that mesenchymal WNT2B is the functionally relevant canonical WNT ligand for gastric stem cell maintenance during homeostasis.

### Acquisition of a KRAS mutation drives the secretion of canonical WNT ligands in the gastric epithelium

**Fig. 2:**
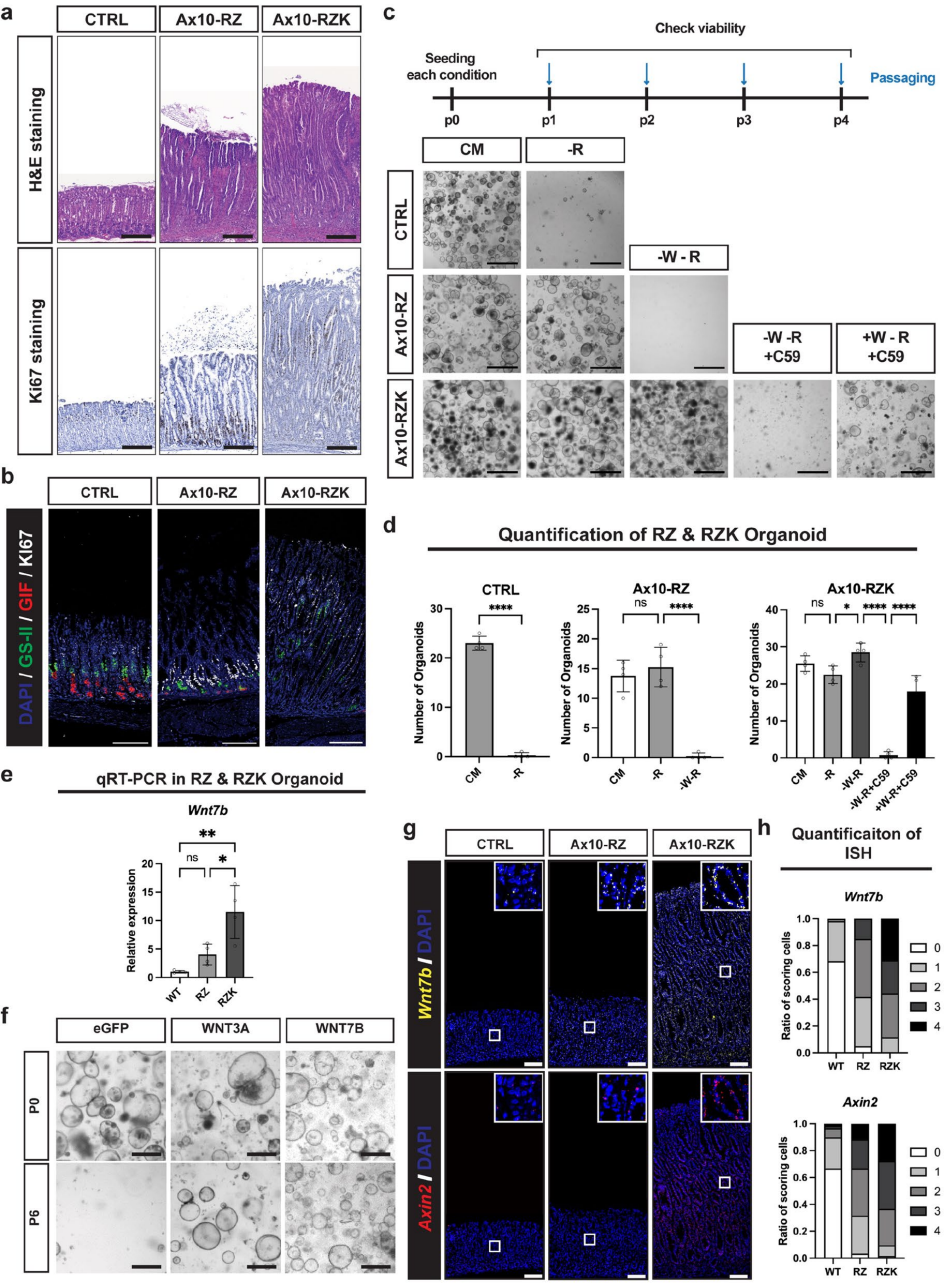
Acquisition of a KRAS mutation drives canonical WNT ligand production in the gastric epithelium. **a** H&E and Ki67 immunohistochemistry of corpus epithelium from control (CTRL), *Anxa10-Cre*^*ERT2*^*; Rnf43*^*f/f*^*; Znrf3*^*f/f*^ (Ax10-RZ), and *Anxa10-Cre*^*ERT2*^*; Rnf43*^*f/f*^*; Znrf3*^*f/f*^*; lsl-Kras*^*G12D*^ (Ax10-RZK) mice, 1 month after tamoxifen induction. CTRL, Ax10-RZ, and Ax10-RZK data are also shown in Supplementary Fig. 3e. Representative images from 2–4 mice per genotype are shown. Scale bars: 100 μm. **b** Immunofluorescence of corpus epithelium from control (CTRL), *Anxa10-Cre*^*ERT2*^*; Rnf43*^*f/f*^*; Znrf3*^*f/f*^ (Ax10-RZ), and *Anxa10-Cre*^*ERT2*^*; Rnf43*^*f/f*^*; Znrf3*^*f/f*^*; lsl-Kras*^*G12D*^ (Ax10-RZK) mice, 1 month after tamoxifen induction. Representative images from 2–4 mice per genotype are shown. Blue: DAPI; Green: GS-Ⅱ (Neck cell marker); Red: Gif (Chief cell marker); White: Ki67. Scale bars: 100 μm. **c** Niche requirements of CTRL, Ax10-RZ, and Ax10-RZK gastric organoids. Organoid growth was examined during 4 passages, except for CTRL; -R and Ax10-RZ; -W-R, which were examined at passage 2. Healthy organoids are cystic with a clear center. Representative images of organoids are shown. CM: complete medium (WENRFG); -R: WENFG; -W-R: ENFG; -W-R + C59: ENFG with C59 (10 μM); + W-R + C59: WENFG with C59 (10 μM). Scale bars: 1000 μm. **d** Organoids in Fig. 2c were quantified by counting those with a diameter of 150 µm or larger. Error bars represent SD. Statistical significance was determined by unpaired t-test (CTRL) and ANOVA (Ax10-RZ, Ax10-RZK). ****, p < 0.00005; *, p < 0.05; ns, non-significant. **e** Bar graph indicating the expression of *Wnt7b*, as determined by qRT-PCR of RNA isolated from CTRL, Ax10-RZ, and Ax10-RZK organoids cultured in CM for 5 days. Expression is normalized to GAPDH. *n* = 4 biological replicates. Error bars represent SD. Statistical significance was determined by ANOVA. **, *p* < 0.005; *, *p* < 0.05; ns, non-significant. **f** Representative organoid images of WNT retrieval assay. Gastric organoids were established from *Rosa26-Cre*.^*ERT2*^ mice and subsequently transduced with retrovirus to overexpress the *Wnt7b* gene. Following tamoxifen treatment, WNT conditioned medium was removed, and organoid growth was observed over six passages. Timeline, control data are the same as in Fig. 1e. P0: passage 0; P6: passage 6. Scale bars: 1000 μm. **g** Representative multiplexed ISH images from CTRL, Ax10-RZ, and Ax10-RZK mouse gastric tissue. Scale bars: 100 μm. **h** The dots in Fig. 2 g were quantified using a semi-quantitative method, and the ratio of cells in each class is represented. Class 0: 0 dots/cell or < 1 dot/10 cells; Class 1: 1–3 dots/cell; Class 2: 4–9 dots/cell, with no or very few clusters; Class 3: 10–15 dots/cell, and/or > 10% of dots are in clusters; Class 4: > 15 dots/cell and/or > 10% of dots are in clusters. Statistical analysis was performed by Fisher’s exact test based on a contingency matrix (Supplementary Table 3)

We and others have previously shown that loss-of-function mutations in RZ confer RSPO1 independence in both the intestine and stomach, although this phenotype still requires a paracrine WNT source [30, 31]. Since *RNF43* mutations are also frequent in gastric cancer [2, 35], we investigated the role of RZ loss-of-function in the stomach. To this end, we used *Anxa10-Cre*^*ERT2*^*; Rnf43*^*f/f*^*; Znrf3*^*f/f*^ (Ax10-RZ) mice (Supplementary Fig. 3a, b), which enabled stomach-specific inducible deletion of RZ [36]. One month after tamoxifen injection, Ax10-RZ mice exhibited gastric gland hyperplasia (Fig. 2a, Supplementary Fig. 3e), characterized by a shifted proliferative zone toward the gland base and a loss of differentiated cell types, compared to wild-type controls (CTRL) (Fig. 2b). Given that the MAPK pathway is frequently activated in gastric cancer (Supplementary Fig. 3c) [37], we next investigated how MAPK pathway activation influences the RZ loss-of-function phenotype. We introduced an oncogenic KRAS mutation (Kras^G12D^) into the RZ-deficient background, generating *Anxa10-Cre*^*ERT2*^*; Rnf43*^*f/f*^*; Znrf3*^*f/f*^*; Kras*^*lsl−G12D*^ (Ax10-RZK) mice (Supplementary Fig. 3 d). One month after induction, Ax10-RZK mice displayed a markedly exacerbated metaplastic phenotype compared to Ax10-RZ and *Anxa10-Cre*^*ERT2*^*; Kras*^*lsl−G12D*^ (Ax10-Kras) mice, including extensive epithelial thickening and widespread Ki67-positive cellular proliferation (Fig. 2a, b, and Supplementary Fig. 3e).

To investigate niche independence of Ax10-RZK cells, we switched from the mouse model to gastric organoid cultures, which provide a controlled proxy for the in vivo niche environment. In these cultures, the medium supplies essential factors, including WNT (WNT3A or Surrogate WNT; W), RSPO1 (R), EGF (E), FGF10 (F), NOGGIN (N), and gastrin (G) [1, 8, 38], that support gastric epithelial growth. Depletion of individual growth factors enables assessment of progressive niche independence in organoids representing different stages of tumor progression. Accordingly, gastric corpus organoids generated from wild-type control, Ax10-RZ, and Ax10-RZK mice were subjected to growth factor withdrawal assays. While wild-type organoids required complete medium (CM) for growth, both Ax10-RZ and Ax10-RZK organoids sustained growth in the absence of RSPO1, consistent with RSPO1 independence previously reported upon RZ loss-of-function [30, 31](Fig. 2c, d). Further, Ax10-RZK organoids showed strong independence from EGF/FGF10 (Supplementary Fig. 3f), accompanied by the highest levels of MAPK pathway activation (Supplementary Fig. 3 g, h), attributed to the oncogenic KRAS mutation. Strikingly, Ax10-RZK organoids continued to grow even in the simultaneous absence of both WNT and RSPO1 (Fig. 2c, d), a capacity not observed in Ax10-RZ organoids. Collectively, these results indicate that oncogenic KRAS activation in the RZ-deficient background not only stimulates the MAPK pathway but also confers WNT independence.

Next, we investigated whether the WNT independence of Ax10-RZK organoids resulted from an acquired ability to self-secrete WNT ligands. To test this, we treated C59, a Porcupine inhibitor that blocks WNT ligand secretion, in WNT- and RSPO-depleted medium. C59 treatment arrested the growth of Ax10-RZK organoids, and this growth defect was rescued by reintroducing exogenous WNT (Fig. 2c, d). These results indicate that the survival of Ax10-RZK gastric organoids in the absence of external WNT requires autonomous secretion of canonical WNT ligand(s) by the epithelial cells. This uncovers an unexpected link between MAPK activation through oncogenic KRAS expression and WNT ligand secretion in the gastric epithelium.

To identify the specific WNT ligands responsible for the WNT secretion phenotype in Ax10-RZK organoids, we compared the expression of *Wnt* genes and canonical WNT target *Axin2* between Ax10-RZ and Ax10-RZK organoids using qRT-PCR (Fig. 2e, Supplementary Fig. 3i) and sn multiome data (Supplementary Fig. 5b). Ax10-RZK organoids exhibited higher expression of *Wnt5a* (via qRT-PCR), *Wnt7b* (via both qRT-PCR and sn multiome), and *Axin2* (via qRT-PCR). Because WNT5A is a non-canonical ligand of WNT signaling and did not support organoid growth in either our overexpression assay (Fig. 1e) or exogenous WNT5A treatment experiment (Supplementary Fig. 3j), it was unlikely to mediate the canonical WNT activity observed in Ax10-RZK organoids. In contrast, WNT7B robustly activated canonical WNT signaling in gastric organoids (Fig. 2f), similar to WNT3A and WNT2B (Fig. 1e). In situ hybridization (ISH) further confirmed strong expression of *Wnt7b* and the WNT target gene *Axin2* throughout the Ax10-RZK gastric epithelium, compared to Ax10-RZ and control mice (Fig. 2g, h, and Supplementary Table 3). Taken together, these results support the notion that WNT7B contributes to canonical WNT activation in the oncogenic KRAS-mutant gastric epithelium.

### KRAS activation primes the spasmolytic polypeptide-expressing metaplasia (SPEM) populations to differentiate into WNT7B producing cells

To investigate changes in gene expression and chromatin accessibility following KRAS activation, we performed sn multiomics—snRNA-seq and snATAC-seq—on wild-type, Ax10-RZ, and Ax10-RZK gastric organoids, yielding a total of 16,920 high-quality cells (WT: 6111, RZ: 5468, RZK: 5341). We first integrated all three conditions using a batch-corrected uniform manifold approximation and projection (UMAP) (Supplementary Fig. 4a). We identified seven distinct epithelial cell clusters by unsupervised clustering and annotated them based on the expression patterns of marker genes of known gastric gland cell types (Supplementary Fig. 4a-c). We detected not only conventional populations found in normal stomach glands—pre-pit (Pre-P), pit (P), neck (N), and proliferating (Pr) cells—but also injury-associated populations such as SPEM (S1 and S2) cells [39, 40]. The S1 cluster expressed early stress-response genes such as *Ero1l* and *Ddit4*; notably, *Ddit4* is known to be transiently upregulated in gastric chief cells to initiate the SPEM transition [41]. The S2 cluster was enriched for *Cd44* and *Glipr1* [42, 43],established markers of SPEM. Because *Lgr5* was broadly distributed across several clusters, including SPEM cells, we inferred that these injury-associated populations possess progenitor-like characteristics. Interestingly, we also identified a novel population characterized by high *Wnt7b* and *Porcn* expression, which we designated as the WNT7 + (W) cluster (Supplementary Fig. 4 d).

**Fig. 3:**
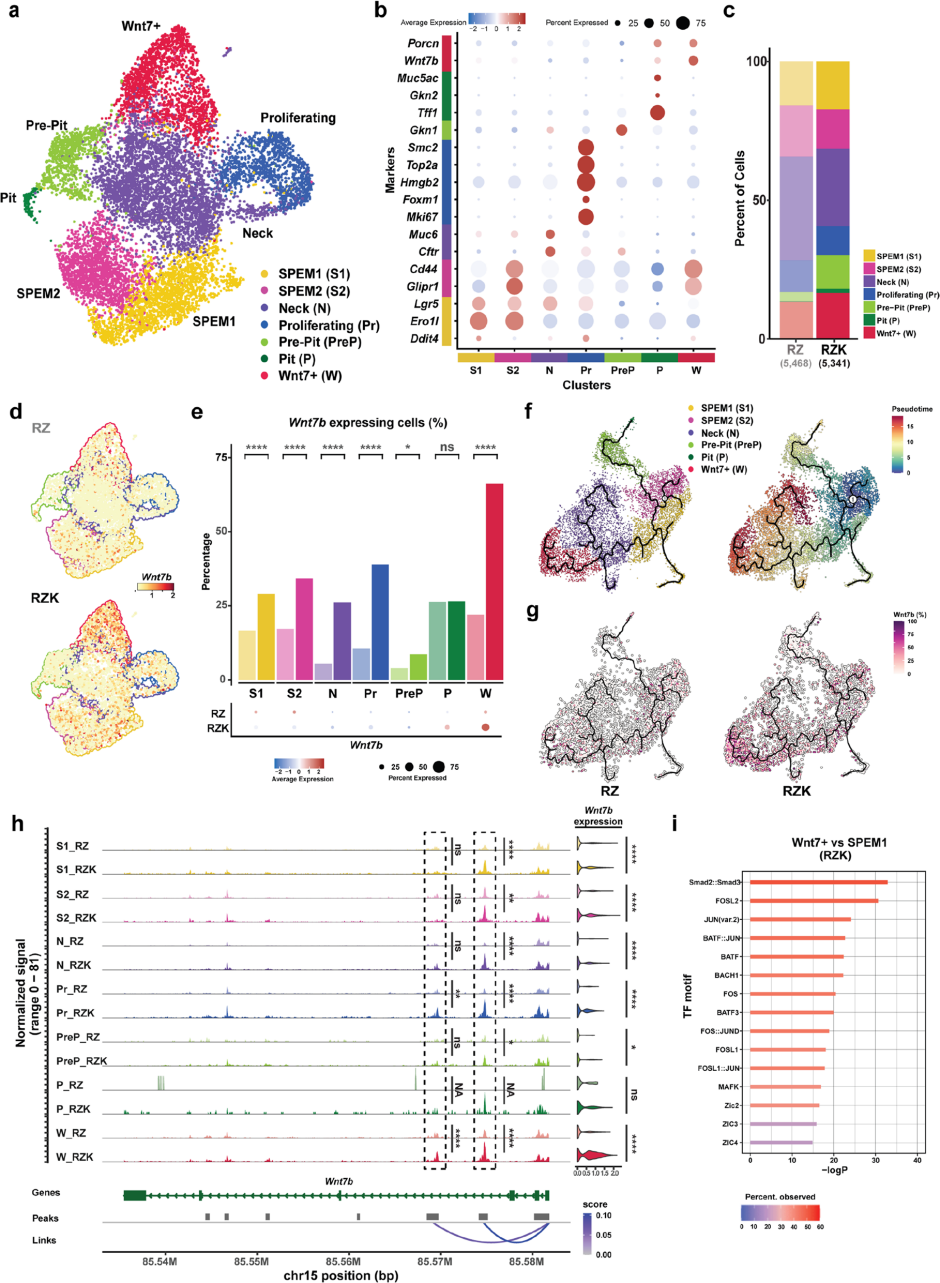
KRAS activation primes the SPEM/Lgr5 + population for differentiation into WNT7B-producing cells. **a** UMAP showing the clustering of epithelial cells from Ax10-RZ and Ax10-RZK organoids based on marker expression: SPEM1, SPEM2, Neck, Proliferating, Pre-Pit, Pit and Wnt7 +. **b** Dot plots showing the expression of marker genes in each cell type. Average expression levels are indicated by color, and the number of expressing cells by dot size, as indicated at the top. S1: SPEM1; S2: SPEM2; N: Neck; Pr: Proliferating; PreP: Pre-Pit; P: Pit; W: Wnt7 +. **c** Fractions of the different epithelial cell types out of the total cells analyzed in Ax10-RZ (lighter colors) and Ax10-RZK (darker colors) organoids. RZ: Ax10-RZ; RZK: Ax10-RZK. **d** Feature plots showing *Wnt7b* gene expression in Ax10-RZ and Ax10-RZK organoids. RZ: Ax10-RZ; RZK: Ax10-RZK. **e** Percentage of WNT7B-expressing cells in the different epithelial cell clusters of Ax10-RZ (lighter colors) and Ax10-RZK (darker colors) organoids. RZ: Ax10-RZ; RZK: Ax10-RZK; S1: SPEM1; S2: SPEM2; N: Neck; Pr: Proliferating; PreP: Pre-Pit; P: Pit; W: Wnt7 +. Statistical analysis was performed by Fisher’s exact test. ****, *p* < 0.00005; *, *p* < 0.05; ns, non-significant. **f** Pseudotime analysis after excluding the Pr cluster. Left: UMAP showing each cluster with trajectory lines Right: Pseudotime analysis was conducted with starting point between the SPEM1 cluster and the SPEM2 cluster set as the origin. **g** *Wnt7b* expression levels in each RZ and RZK are shown as percentages relative to highest expression in the combined group. Left: Relative *Wnt7b* expression in Ax10-RZ organoids. Right: Relative *Wnt7b* expression in Ax10-RZK organoids. **h** ATAC-seq peaks in different epithelial cell clusters of Ax10-RZ (lighter colors) and Ax10-RZK (darker colors) organoids within the *Wnt7b* locus. Colors represent different cluster identities. Statistical analysis of ATAC peaks and RNA expression was performed by student's t-test and a negative binomial test, respectively. ****, *p* < 0.00005; *, *p* < 0.05; ns, non-significant; NA, not applicable. **i** Motif enrichment analysis from differential accessible peak analysis using sn multiome data from Wnt7 +, compared to the SPEM1 cluster in Ax10-RZK. Enrichment is depicted as a percentage on a -logP scale

Next, we asked how Kras activation alters epithelial cell states and induces WNT7B-expressing populations. We compared the Ax10-RZ and Ax10-RZK gastric organoid UMAPs to evaluate changes within a shared genetic background (RZ) (Fig. 3a, b). This approach ensured that the observed differences were specifically attributable to Kras activation (Supplementary Fig. 5a). We observed only minor differences in the percentage of each cell cluster, except for a clear decrease in the N cluster and a notable increase in the PreP/P clusters in Ax10-RZK samples (Fig. 3c). Nevertheless, we observed increased expression of *Wnt7b* in Ax10-RZK organoids as expected (Fig. 3d), primarily in the W cluster. We also observed an increase in the percentage of *Wnt7b* expressing cells in the W cluster, but also at lower levels in other clusters, except for the P cluster (Fig. 3e). Next, we performed a trajectory analysis using the S1 and S2 clusters as a starting point. Here, the P, a subpopulation of the N, and the W clusters represented the mature populations in the trajectory (Fig. 3f). When we separated the trajectory map into RZ and RZK conditions, cells in the RZ condition primarily tended to differentiate into neck cells, while those in the RZK condition altered their differentiation paths toward pit or WNT7-expressing cells (Fig. 3g). This suggests that KRAS activation drives cells toward a more mature state, correlating with previous observations of MAPK signaling-dependent pit cell differentiation [44]. Of note, one of these mature cell states is expressing WNT7B, which in turn explains the WNT niche independence of RZK organoids.

To investigate how KRAS activation leads to WNT7B expression, we analyzed the snATAC-seq profiles of different clusters from our sn multiome dataset. This allowed us to compare DNA accessibility at the *Wnt7b* locus between the Ax10-RZ and Ax10-RZK organoid genomes (Fig. 3h). The *Wnt7b* locus showed seven distinct peaks, indicative of transcription factor binding sites and regions of open chromatin. The first three peaks, indicating internal enhancer regions, were highly correlated with *Wnt7b* RNA expression levels. Compared to Ax10-RZ, Ax10-RZK gastric organoids showed significantly higher peaks at these enhancer regions within the *Wnt7b* gene locus across all cell clusters, documenting more open chromatin in almost all cells (Fig. 3h). In fact, increased chromatin openness was already apparent in progenitor populations, such as S1, S2, and Pr cells, with significantly higher expression of *Wnt7b*—most notably in the W cluster (Fig. 3e, h). These data suggest that KRAS activation initiates the opening of *Wnt7b* enhancers already in progenitor cells, resulting in robust epithelial WNT production by WNT7B-expressing cells (W cluster).

Lastly, to identify candidate transcription factors (TFs) driving this KRAS-driven WNT production, we performed motif enrichment analysis (MEA) between the S1 and W clusters in Ax10-RZ and Ax10-RZK gastric organoids, respectively. This analysis suggested that motifs of SMAD2/3 and the AP-1 complex proteins FOS and JUN are potential mediators driving *Wnt7b* expression in RZK cells (Fig. 3i and Supplementary Fig. 5c).

### SMAD2/3 mediates *Wnt7b* expression under KRAS-mediated MAPK activation

To elucidate the molecular mechanism underlying KRAS activation-mediated WNT secretion, we first focused on one of the candidate TFs, SMAD2/3, identified as the most enriched factor in the TF-MEA (Fig. 3i). SMAD2/3 functions downstream of transforming growth factor-beta (TGFβ) signaling, which involves TGFβ ligands (TGFβ1, TGFβ3), TGFβ receptors (TGFβRI, TGFβRII), and the SMAD2/3 TF complex [45] (Supplementary Fig. 6a). TGFβ ligands initially bind to TGFβRII, which then recruits TGFβRI to form a heteromeric receptor complex. Within this complex, TGFβRII phosphorylates TGFβRI, and activated TGFβRI subsequently phosphorylates SMAD2/3 on their C-terminal domains. Phosphorylated SMAD2/3 (p-SMAD2/3) then translocate to the nucleus, where they function as an active transcription factor complex.

**Fig. 4:**
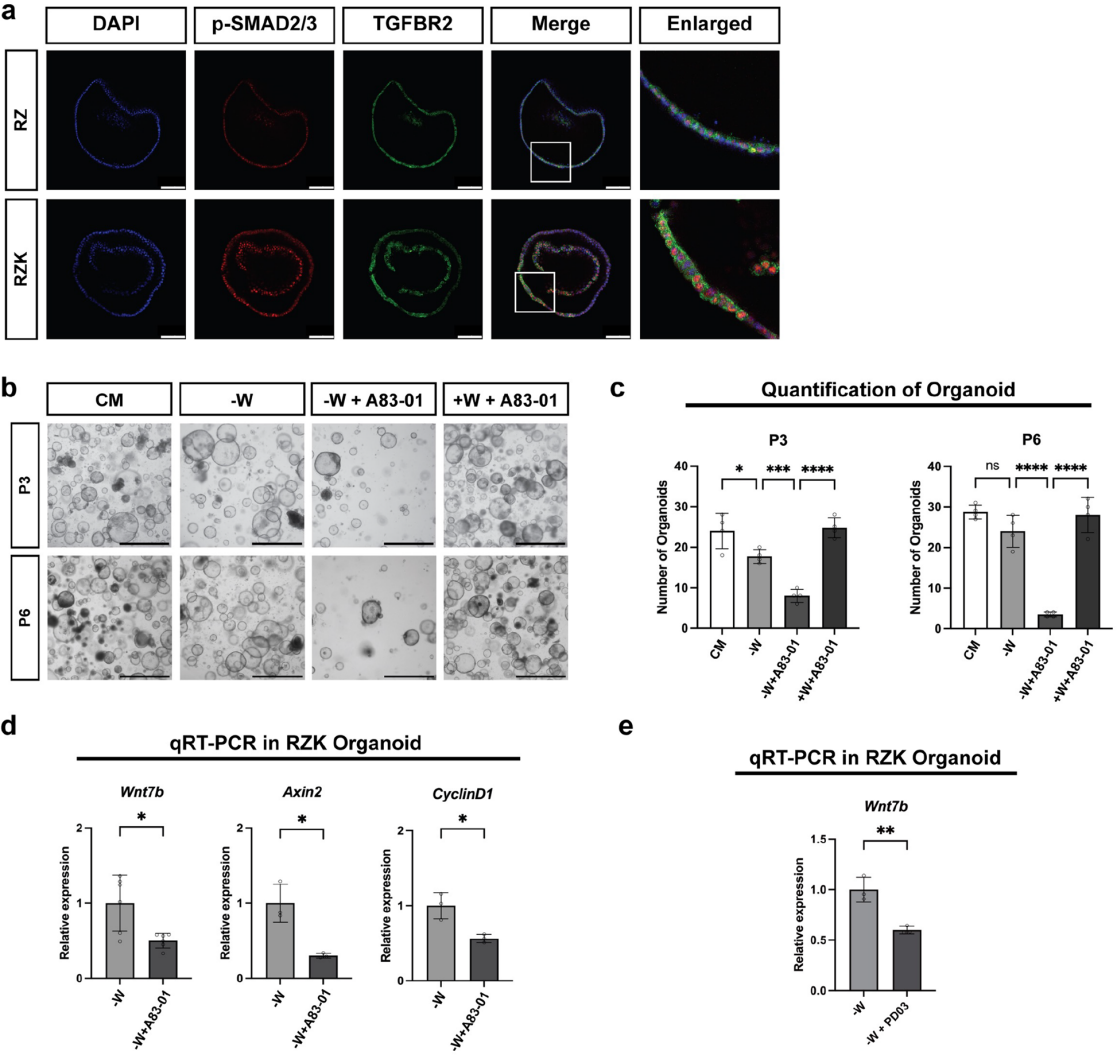
SMAD2/3 regulates *Wnt7b* expression under KRAS-mediated MAPK activation. **a** Representative immunofluorescence images of Ax10-RZ and Ax10-RZK organoids. Blue: DAPI; Red: p-SMAD2/3; Green: TGFBR2. Scale bars: 100 μm. **b** TGF-β inhibitor treatment on Ax10-RZK gastric organoids. Organoid growth was examined after 3 and 6 passages with drug treatment. Healthy organoids are cystic with a clear center. Representative images of organoids are shown. CM: complete medium (WENRFG); -W: ENRFG; -W + A83-01: ENRFG + A83-01(2 µM); + W + A83-01: WENRFG + A83-01(2 µM). Scale bars: 1000 μm. **c** Organoids in Fig. 4b were quantified by counting those with a diameter of 150 µm or larger. Error bars represent SD. Statistical significance was determined by ANOVA. ****, *p* < 0.00005; ***, *p* < 0.0005; *, *p* < 0.05; ns, non-significant. **d** Bar graphs indicating the expression of *Wnt7b* and WNT downstream genes, as determined by qRT-PCR of RNA isolated from RZK organoids cultured in -W medium and -W medium with A83-01(2 µM) conditions (in Fig. 4b). Expression is normalized to GAPDH. n = 3—6 biological replicates. Error bars represent SD. Statistical significance was determined by paired t-test. *, *p* < 0.05. **e** Bar graphs indicating the expression of *Wnt7b* gene, as determined by qRT-PCR of RNA isolated from RZK organoids under -W medium and -W medium with PD0325901(1 µM) conditions. Expression is normalized to GAPDH. n = 3 biological replicates. Error bars represent SD. Statistical significance was determined by paired t-test. *, *p* < 0.05

Compared with RZ organoids, RZK organoids showed higher expression of *Tgfbr2*, which encodes TGFβRII, predominantly in the WNT7 + cluster in the sn multiome data (Supplementary Fig. 5 d). Consistent with this, regions with high TGFβRII expression in RZK correlated with increased nuclear p-SMAD2/3 compared with RZ (Fig. 4a). This is in line with the fact that TGFβ pathway activation is highly dependent on TGFβRII expression, because TGFβRI alone has very weak binding affinity for TGFβ and cannot effectively initiate signaling without TGFβRII [46, 47].

To assess the functional involvement of p-SMAD2/3 in WNT7B secretion in RZK organoids, we treated the organoids with A83-01, a potent TGFβRI inhibitor that prevents SMAD2 phosphorylation (Supplementary Fig. 6b) [48]. A83-01 treatment caused a marked reduction in organoid growth over successive passages, which was fully rescued by the addition of WNT to the medium (Fig. 4b, c). Consistent with this, *Wnt7b* expression was significantly reduced in qRT-PCR analysis of A83-01 treated RZK organoids, accompanied by decreased expression of *Axin2* and *CyclinD1*, two downstream targets of the WNT pathway (Fig. 4d). We further confirmed that the expression of *Wnt7b* and WNT target genes is dependent on TGFβ signaling by treating RZK organoids two additional TGFβ pathway inhibitors, LY2109761 [49] and ITD-1 [50, 51] (Supplementary Fig. 6c). Aside from SMAD2/3, highly accessible chromatin regions included motifs related to the AP-1 complex—FOS and JUN (Fig. 3i). However, treating RZK organoids with the AP-1 complex inhibitor T-5224 [52] resulted in only a subtle decrease in *Wnt7b* expression (Supplementary Fig. 6 d). Taken together, these findings indicate that regulation of *Wnt7b* expression in RZK organoids is primarily mediated by SMAD2/3.

Next, we questioned how the KRAS mutation activates TGFβ signaling. Because receptor tyrosine kinase (RTK) pathway, where KRAS is a key component, primarily diverges into the MAPK and phosphoinositide 3-kinase (PI3K) pathways (Supplementary Fig. 6a), we inhibited either MEK using PD0325901 or PI3K using LY294002 in RZK organoids (Supplementary Fig. 6 g). MEK inhibition prevented the growth of RZK organoids, and this defect was rescued by the addition of external WNT, whereas PI3K inhibition had no significant effect (Supplementary Fig. 6e-g). We further confirmed that MEK inhibition significantly downregulated *Wnt7b* expression (Fig. 4e), likely through reduction in *Tgfbr2* expression (Supplementary Fig. 6 h) rather than through direct effects on SMAD2/3 phosphorylation (Supplementary Fig. 6b). AP-1 inhibition caused only a mild reduction in *Tgfbr2* expression, suggesting the involvement of multiple TFs in regulating *Tgfbr2* (Supplementary Fig. 6 h). Altogether, these results suggest that WNT secretion in RZK organoids is driven by a MAPK-TGFβRII-SMAD2/3 axis.

### MAPK activation by KRAS mutation drives canonical WNT secretion regardless of the mechanism of RSPO independence

**Fig. 5:**
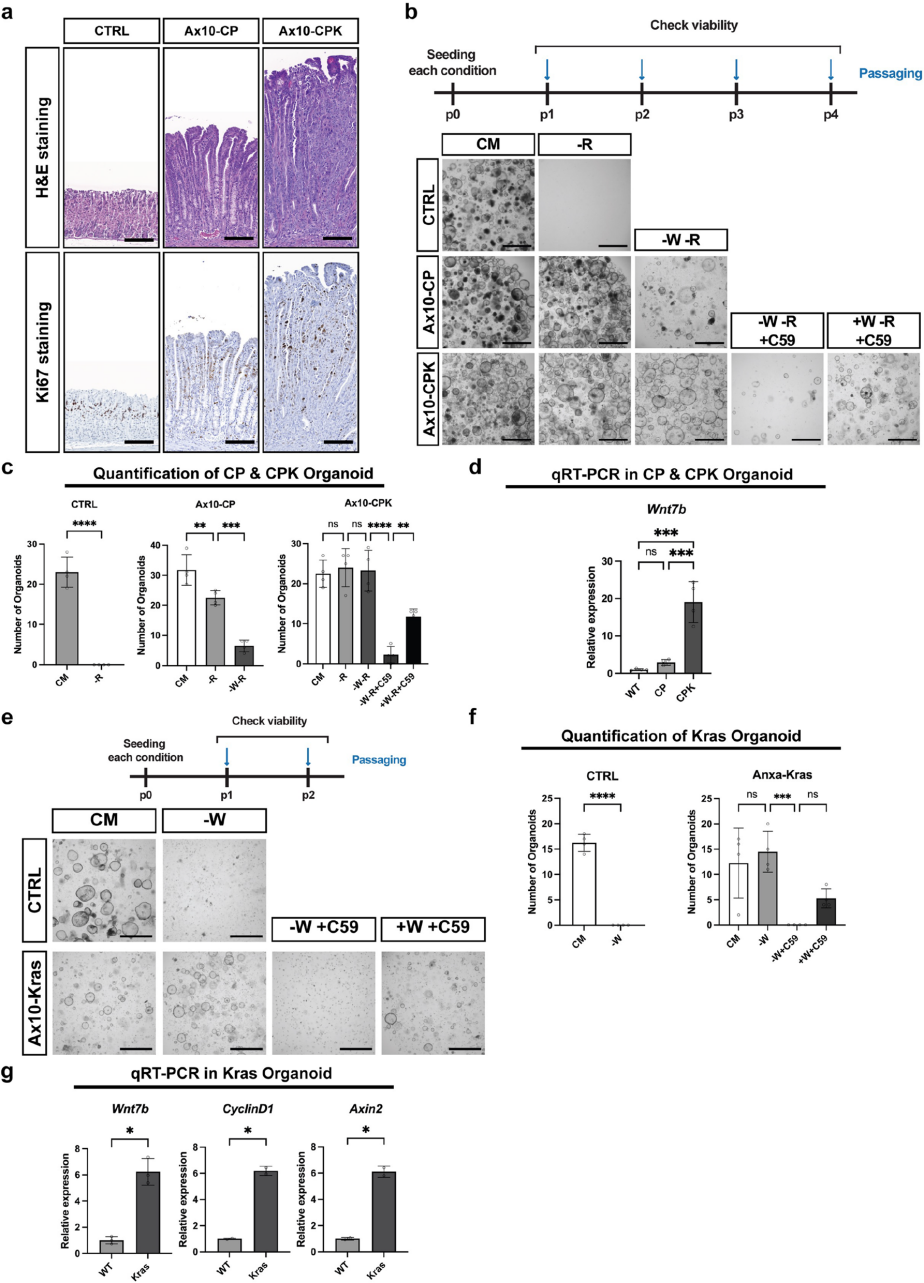
Acquired KRAS mutation drives canonical WNT secretion regardless of RSPO independence. **a** H&E and Ki67 immunohistochemistry of corpus epithelium from control (CTRL), *Anxa10-Cre*^*ERT2*^*; Cdh1*^*f/f*^*; Tp53*^*f/f*^ (Ax10-CP), and *Anxa10-Cre*^*ERT2*^*; Cdh1*^*f/f*^*; Tp53*^*f/f*^*; lsl-Kras*^*G12D*^ (Ax10-CPK) mice, 1 month after tamoxifen induction. CTRL, Ax10-CP, and Ax10-CPK images are also shown in Supplementary Fig. 7e. Representative images from 2–4 mice per genotype are shown. Scale bars: 100 μm. **b** Niche requirements of CTRL, Ax10-CP, and Ax10-CPK gastric organoids. Organoid growth was examined during 4 passages, except for CTRL; -R, which was examined at passage 2. Healthy organoids are cystic with a clear center. Representative images from organoids isolated from 2–4 mice per genotype are shown. CM: complete medium (WENRFG); -R: WENFG; -W-R: ENFG; -W-R + C59: ENFG with C59 (10 μM); + W-R + C59: WENFG with C59 (10 μM). Scale bars: 1000 μm. **c** Organoids in Fig. 5b were quantified by counting those with a diameter of 150 µm or larger. Error bars represent SD. Statistical significance was determined by unpaired t-test (CTRL) and ANOVA (Ax10-CP, Ax10-CPK). ****, *p* < 0.00005; ***, *p* < 0.0005; **, *p* < 0.005; ns, non-significant. **d** Bar graph indicating the expression of *Wnt7b,* as determined by qRT-PCR of RNA isolated from CTRL, Ax10-CP, and Ax10-CPK organoids cultured in CM for 5 days. Expression is normalized to GAPDH. *n* = 4 biological replicates. Error bars represent SD. Statistical significance was determined by ANOVA. ***, *p* < 0.0005; ns, non-significant. **e** Niche requirements of CTRL and *Anxa10-Cre*^*ERT2*^*; lsl-Kras*.^*G12D*^ (Ax10-Kras) gastric organoids. Organoid growth was examined during 2 passages. Healthy organoids are cystic with a clear center. Representative images of organoids isolated from 2–4 mice per genotype are shown. CM: complete medium (WENRFG); -R: WENFG; -W: ENRFG; -W + C59: ENRFG with C59 (10 μM); + W + C59: WENRFG with C59 (10 μM). Scale bars: 1000 μm. **f** Organoids in Fig. 5e were quantified by counting those with a diameter of 150 µm or larger. Error bars represent SD. Statistical significance was determined by unpaired t-test (CTRL) and ANOVA (Ax10-Kras). ****, *p* < 0.00005; ***, *p* < 0.0005; ns, non-significant. **g** Bar graph indicating the expression of *Wnt7b* and WNT downstream genes, as determined by qRT-PCR of RNA isolated from CTRL and Ax10-Kras organoids cultured in NRG medium for 5 days. Expression is normalized to GAPDH. n = 3 biological replicates. Error bars represent SD. Statistical significance was determined by paired t-test. *, *p* < 0.05

Following our finding that KRAS-mediated SMAD2/3 activation, coupled with RZ loss-of-function, leads to WNT secretion in the gastric epithelium, we questioned whether this phenotype specifically depends on the combination of these three mutations (RZK). It has been shown that the gastric epithelium can obtain RSPO independence not only by losing RZ function but also by the combined loss of E-**C**adherin (*Cdh1*) and T**P**53 (*Tp53*) (CP) [31]. To model this alternative path to RSPO1 niche independence in mice, we introduced a floxed knockout allele of *Cdh1* (*Cdh1*^*f/f*^) and a point-mutation allele of *p53* that results in a null phenotype (*Tp53*^*R172H*^), under the control of *Anxa10-Cre*^*ERT2*^ (Ax10-CP) (Supplementary Fig. 7a, b). To investigate the effect of *Kras* activation, we additionally introduced *Kras*^*lsl−G12D*^, generating a triple mutant mouse (Ax10-CPK) (Supplementary Fig. 7c). In parallel, we introduced a *Tp53* knockout to Ax10-C and Ax10-CK organoids to generate CP and CPK organoids, respectively (Supplementary Fig. 7 d). Tamoxifen induction led to gastric epithelial hyperplasia and cellular proliferation in Ax10-CP mice, which was further enhanced in Ax10-CPK mice (Fig. 5a, Supplementary Fig. 7e, f). Growth factor withdrawal from wild-type, CP, and CPK gastric organoids resulted in phenotypes that were similar to those observed in RZ and RZK organoids. CP and CPK organoids survived multiple passages in a medium without RSPO1; however, only CPK organoids maintained continuous growth following the withdrawal of both WNT and RSPO1 (Fig. 5b, c), indicating that CPK organoids are WNT self-sufficient, similar to RZK organoids. We further investigated whether CPK organoids secreted WNT as well, by treating the organoids with the WNT secretion inhibitor C59. Indeed, CPK organoids could not be maintained in the presence of C59 but were rescued by the addition of WNT (Fig. 5b, c), further validating that they secrete WNT. To check whether WNT7B is involved, we compared the expression of *Wnt7b* in CP and CPK organoids using qRT-PCR (Fig. 5d). Expression of *WNT7b* was highly upregulated in CPK organoids, implicating a similar mechanism of WNT7B self-secretion by gastric epithelial cells, as observed in the RZK model (Fig. 2e). Taken together, these results suggest that WNT independence in the gastric epithelium through KRAS-induced WNT7B self-secretion is not limited to cells with RZ loss-of-function, but can also be alternatively achieved through CP mutations.

One remaining question is whether WNT independence can only be obtained subsequent to RSPO1 independence or also directly by the acquisition of KRAS activation. To investigate this, we generated gastric organoids from *Anxa10-Cre*^*ERT2*^*; Kras*^*lsl−G12D*^ (Ax10-Kras) mice (Supplementary Fig. 7c, g) and compared their growth to organoids from wild-type mice (Fig. 5e). While wild-type organoids died following WNT removal, Ax10-Kras organoids sustained growth without WNT (Fig. 5e, f). Treatment with C59 inhibited the growth of Ax10-Kras organoids in WNT-depleted medium, which could be rescued by the reintroduction of WNT to the medium (Fig. 5e, f). Thus, gastric organoids with KRAS oncogenic mutation are capable of self-secreting WNT ligands. qRT-PCR of Ax10-Kras organoids revealed elevated expression levels of *Wnt7b* and its downstream targets, *Axin2* and *CyclinD1* (Fig. 5g).

We further assessed WNT7B secretion in organoids from another mouse model, *Mist1-Cre*^*ERT2*^*; Kras*^*lsl−G12D*^, where *Kras* oncogenic mutations were specifically induced in chief cells through tamoxifen treatment (Supplementary Fig. 7 h) [13, 53, 54]. qRT-PCR of gastric organoids derived from these mice, either one month (Meta1, SPEM-like) or four months (Meta4, dysplastic) following tamoxifen treatment, revealed significantly higher expression levels of *Wnt7b* in Meta4 organoids compared to Meta1. These results show that as the KRAS activation persists, *Wnt7b* expression increases, potentially leading to a more niche-independent state of cells (Supplementary Fig. 7 h). In sum, we conclude that KRAS activation causes a niche escape phenotype in the gastric epithelium via WNT7B expression regardless of RSPO independence. Nevertheless, the RSPO independence substantially aggravated the phenotype of WNT secretion, potentially by better usage of the available WNT ligand (Supplementary Fig. 7e).

### Effect of KRAS activation in different tissues

Next, to investigate whether KRAS-mediated WNT7B secretion is conserved across different organs, we utilized the *Rosa26-Cre*^*ERT2*^; *Red2-Kras*^*G12D*^ mouse, in which only RFP + cells, among the four Confetti colors (nuclear GFP, YFP, RFP, and membrane CFP) express *Kras*^*G12D*^ upon Cre-mediated recombination [55] (Supplementary Fig. 7i). We established and stabilized organoids from the small intestine (SI) and pancreas.

For SI organoids, we isolated RFP + KrasG12D cells and compared their phenotype with Confetti control organoids, in which no cells harbor the Kras mutation. Under normal ENR culture conditions, Confetti control organoids exhibited typical budding morphology, whereas RFP + SI organoids expressing *Kras*^*G12D*^ remained cystic, indicative of elevated WNT pathway activity (Supplementary Fig. 7j). qRT-PCR analysis further showed that RFP + SI organoids expressed high levels of *Wnt7b*, as well as downstream WNT targets *CyclinD1* and *Axin2*, indicating that active KRAS induces epithelial WNT7B secretion in the small intestine (Supplementary Fig. 7 k).

In contrast, when we compared YFP + wild-type cells with RFP + *Kras*^*G12D*^ cells after sorting and stabilizing in pancreatic organoids, KRAS activation did not induce *Wnt7b* expression (Supplementary Fig. 7 l), suggesting that the effects of KRAS activation on WNT7B expression are tissue-dependent.

### MAPK activation and WNT2 copy number gain in gastric cancer patient-derived organoids correlate with epithelial WNT secretion

**Fig. 6:**
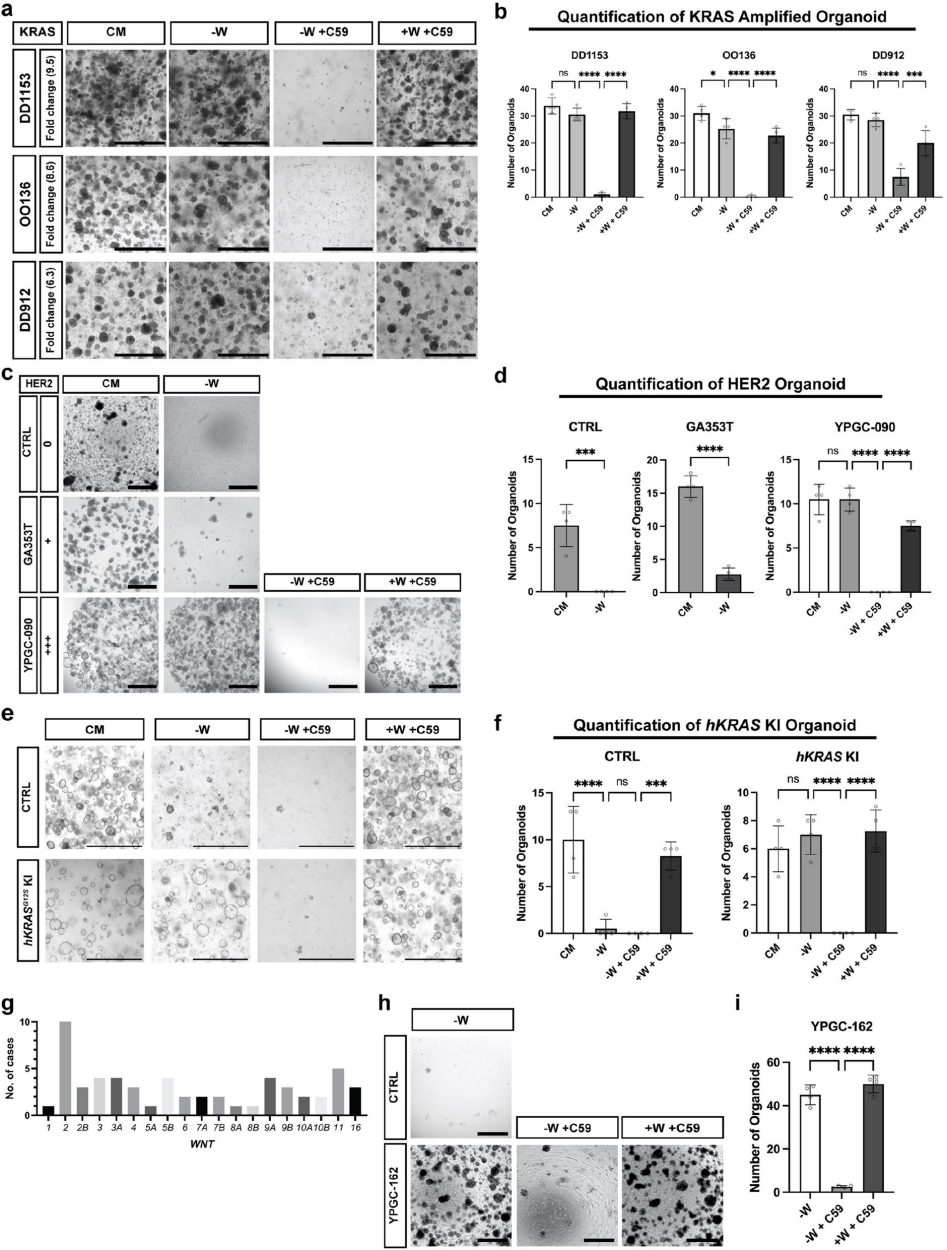
MAPK activity and WNT2 copy number gain in gastric cancer patient-derived organoids correlate with WNT independence. **a** Niche requirements of human gastric cancer patient-derived organoid lines with KRAS amplification. Organoid growth was examined after 6 passages Healthy organoids are cystic with a clear center or growing in grape-like structures. CM: complete medium (WENRFG); -W: ENRFG; -W + C59: ENRFG medium with C59 (1 μM); + W + C59: WENRFG with C59 (1 μM). Scale bars: 1000 μm. **b** Organoids in Fig. 6a were quantified by counting those with a diameter of 150 µm or larger. Error bars represent SD. Statistical significances was determined by ANOVA test. ****, *p* < 0.00005; ***, *p* < 0.0005; ns, non-significant. **c** Niche requirements of human gastric cancer patient-derived organoid lines with HER2 activation. HER2 levels are classified as negative (0, +) and positive (+ + +) based on guidelines published by Bartley et al. [56]. Organoid growth was examined after 3 passages. Healthy organoids are cystic with a clear center or growing in grape-like structures. CM: complete medium (WENRFG); -W: ENRFG; -W + C59: ENRFG medium with C59 (10 μM); + W + C59: WENRFG with C59 (10 μM). Scale bars: 1000 μm. **d** Organoids in Fig. 6c were quantified by counting those with a diameter of 150 µm or larger. Error bars represent SD. Statistical significances was determined by unpaired t-test (CTRL, GA353T), ANOVA test (YPGC-090). ****, *p* < 0.00005; ***, *p* < 0.0005; ns, non-significant. **e** Niche requirements for human gastric organoid lines (WT) and organoids targeted with human *KRAS*^*G12S*^ knock-in (*hKRAS*^*G12S*^ KI). Organoid growth was examined after 2 passages. Healthy organoids are cystic with a clear center. CM: complete medium (WENRFG); -W: ENRFG; -W + C59: ENRFG with C59 (10 μM); + W + C59: WENRFG with C59 (10 μM). Scale bars: 1000 μm. **f** Organoids in Fig. 6e were quantified by counting those with a diameter of 150 µm or larger. Error bars represent SD. Statistical significances was determined by ANOVA test. ****, *p* < 0.00005; ***, *p* < 0.0005; ns, non-significant. **g** The TCGA stomach adenocarcinoma dataset was analyzed using the cBioPortal platform. Copy number alteration profiles were obtained in GISTIC 2.0 format, and a value of + 2 was classified as amplification. All *Wnt* family genes were included in the analysis. **h** Niche requirements of human gastric organoid lines (WT) and human gastric cancer patient-derived organoid lines with WNT2 gene amplification. Organoid growth was examined after 3 passages. Healthy organoids are cystic with a clear center or grow in grape-like structures. -W: ENRFG; -W + C59: ENRFG medium with C59 (10 μM); + W + C59: WENRFG with C59 (10 μM). Scale bars: 1000 μm. **i** Organoids in Fig. 6 h were quantified by counting those with a diameter of 150 µm or larger. Error bars represent SD. Statistical significances was determined by ANOVA. ****, *p* < 0.00005; ns, non-significant

To investigate whether the mechanisms of WNT independence identified in mice are also relevant to human gastric cancer development, we first tested WNT secretion phenotype in gastric cancer patient-derived organoids (GC-PDOs) with *KRAS* gene alterations. We collected three GC-PDOs with KRAS amplification, DD1153, OO136, and DD912, all of which showed marked increase in KRAS gene copy number (Supplementary Fig. 8a). Notably, all three PDO lines displayed a clear WNT secretion phenotype (Fig. 6a, b), suggesting that the KRAS-WNT secretion axis is conserved in humans.

Gene amplification and subsequent overexpression of *ERBB2* (the gene encoding HER2), an RTK acting upstream of the KRAS-MAPK pathway, are also commonly found in gastric cancers (Supplementary Fig. 3c). To explore whether HER2-driven MAPK activation similarly promotes epithelial WNT secretion, we tested two GC-PDO lines GA353T and YPGC-090, which express either low or very high levels of HER2 and are classified by standard immunohistochemistry testing as HER2 negative or HER2 triple-positive (+ + +), respectively (Supplementary Fig. 8b). While the HER2 negative GA353T line was unable to grow in the absence of WNT in the culture medium, the HER2 + + + line YPGC-090 grew robustly in WNT-deficient medium (Fig. 6c, d). Of note, inhibition of WNT secretion by C59 abolished the growth of YPGC-090 organoids, and this growth defect was fully rescued by reintroducing exogenous WNT, demonstrating dependence on epithelial WNT secretion. To validate these findings, we set up a collection of GC-PDOs from primary cancers with varying levels of HER2 expression (Supplementary Fig. 8b) [56, 57]. GC-PDO lines with high HER2 expression (GA372T, YPGC-021, −057, −075, −082, −090, −105, −162, DD-191, OO9, OO14, and OO66) could all be maintained without exogenous WNT (Supplementary Fig. 8c, d). In 10 out of 12 HER2 + + + GC-PDOs, C59 treatment substantially reduced organoid growth, and this effect was again rescued by reintroducing WNT to the medium (Supplementary Fig. 8c, d). These results suggest that HER2-mediated MAPK activation confers WNT independence in human gastric cancer by promoting epithelial WNT secretion.

In the case of GC-PDOs YPGC-057 and −082, both classified as HER2 + + +, organoid growth was not inhibited or only mildly inhibited by C59 treatment. Interestingly, copy number variation (CNV) analysis across all organoid lines (Supplementary Fig. 8e) revealed a substantial increase in the copy number of *MYC*, a well-known downstream target of WNT-β-catenin signaling, specifically in YPGC-057 and YPGC-082 (copy numbers of 57 and 44, respectively) [58, 59]. This extremely high *MYC* amplification may compensate for the inhibition of WNT secretion by C59.

Although HER2 immunostaining confirmed that all samples were HER2 + + +, CNV analysis revealed only a slight or no increase in *ERBB2* copy number in GC-PDOs OO9, OO14, OO66, and DD-191 (Supplementary Fig. 8b, e). To investigate this discrepancy, we performed RNA sequencing on these lines. The analysis showed elevated *ERBB2* expression compared to the average expression across a large cohort of gastric cancers and normal stomach tissues from public datasets, along with high *WNT7B* expression (Supplementary Fig. 8f). These results indicate that non-amplification mechanisms leading to *ERBB2* overexpression can also result in elevated *WNT7B* levels. Collectively, these findings suggest that enhanced MAPK signaling activation in GC-PDOs correlates with increased *WNT7B* expression, consistent with our observations in mouse models.

To determine whether *WNT7B* expression correlates with *KRAS* mutation status and gastric cancer stage, we analyzed TCGA-STAD dataset. After excluding tumors harboring mutations in upstream RTK genes (*ERBB2*, *ERBB3*, and *FGFR2*), we categorized the remaining cases into *KRAS* wild type (*KRAS* WT) and *KRAS* oncogenic alteration (*KRAS* mutant) groups. Notably, *WNT7B* expression was significantly higher in tumors with *KRAS* mutations (Supplementary Fig. 8 g). *WNT7B* expression also tended to increase in more advanced stages of gastric cancer (Supplementary Fig. 8 h), supporting the idea that *WNT7B* serves as a marker of MAPK pathway activation and cancer progression.

Gastric cancer in general as well as the analyzed GC-PDOs harbor a plethora of additional molecular alterations in addition to HER2 overexpression [37]. To clearly confirm that the MAPK activation leads to WNT secretion, we directly assessed the effects of KRAS activation in human gastric organoids using clustered regularly interspaced short palindromic repeats (CRISPR)/Cas9 gene editing. We knocked in an oncogenic variant frequently found in human gastric cancer (hKRAS^G12S^) into the gene locus of a normal human gastric organoid line which was confirmed that no additional oncogenic alterations are present via WGS. Unlike the parental normal organoid line, hKRAS^G12S^-knock-in (KI) organoids were able to grow in the absence of WNT (Fig. 6e, f), which could be inhibited by C59 and rescued upon re-introduction of WNT to the medium (Fig. 6e, f). Thus, KRAS activation in the human gastric epithelium leads to epithelial WNT secretion, as observed in the other examples above.

To investigate whether *WNT* genes could also be direct targets of gene alterations in human gastric cancer, we checked the Cancer Genome Atlas (TCGA) dataset. Interestingly, *WNT* gene copy number alterations were observed in all 19 *WNT* genes, with *WNT2* being predominantly altered (Fig. 6g). WNT2B, a WNT2 paralogue, was the canonical WNT ligands we found to be expressed in mouse gastric *Robo2* high fibroblasts (Supplementary Fig. 1c, d), and functionally capable of maintaining gastric epithelial proliferation (Fig. 1e). Within our GC-PDO cohort, we identified several lines with a *WNT2* copy number gain, with one line (YPGC-162) showing a high amplification (10 copies) (Supplementary Fig. 8e). YPGC-162 was able to maintain growth in medium without WNT, which was once again prevented by C59 and rescued by reintroduction of WNT (Fig. 6h, i). The line also showed additional copy number gains at both *ERBB2* and *KRAS* loci, which might cooperate to achieve epithelial WNT secretion for niche escape.

### The KRAS-WNT7B axis is active across multiple cancer types

To investigate whether the KRAS-WNT7B axis is conserved across cancers from different tissues, we analyzed cancer cell lines from various tissue origins using public datasets. Since our data showed that WNT7B is secreted by KRAS-activated cancer cells rather than the surrounding niche, we focused on cancer cell lines which lack other niche cell types.

**Fig. 7:**
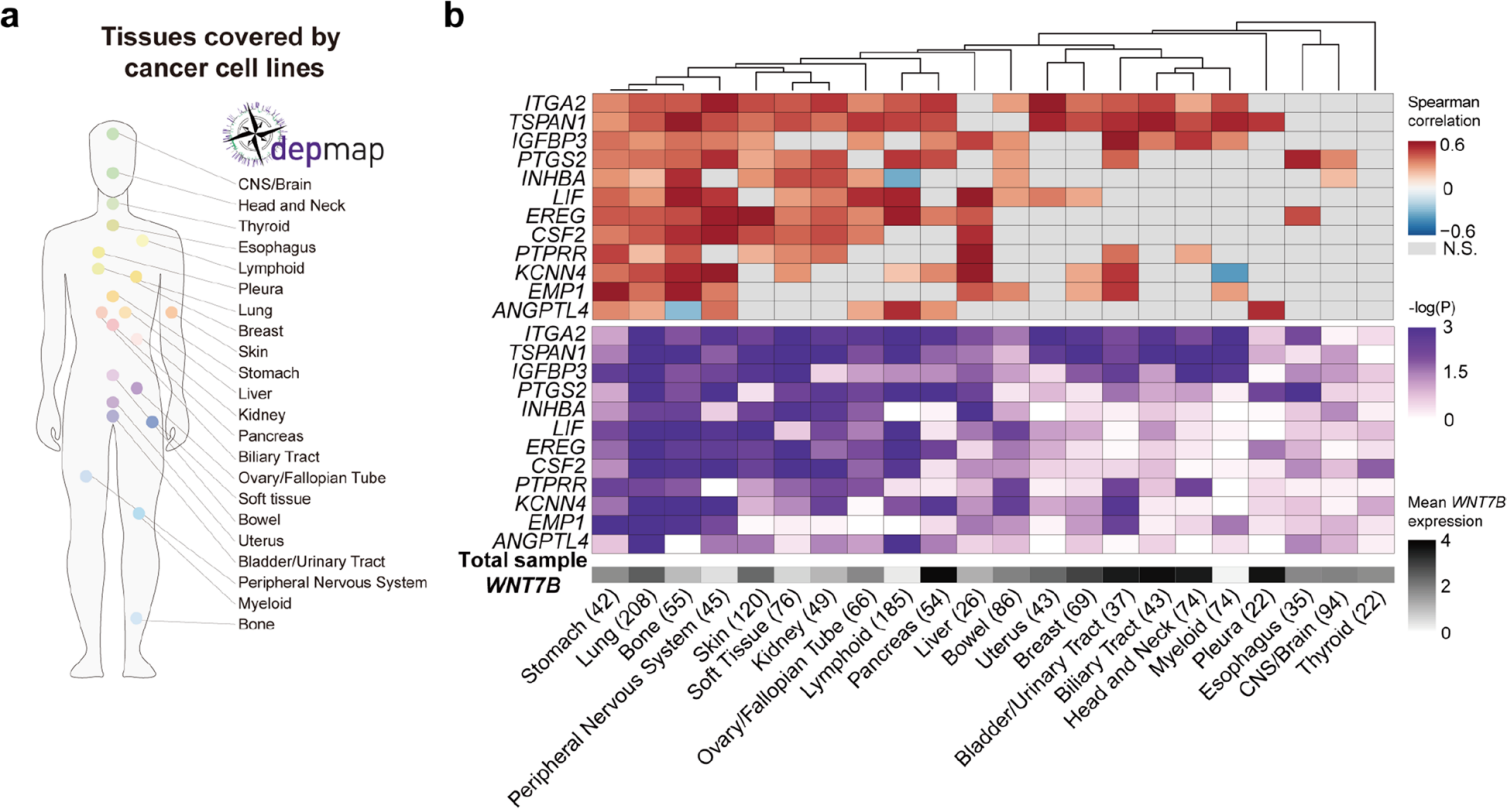
Identification of the KRAS-WNT7B axis across multiple organs using cancer cell line data. **a** Cell lines derived from 22 tissues including more than 20 cases were analyzed using data from DepMap database. **b** Heatmaps showing correlations between *WNT* expression and KRAS signature genes. Upper: Spearman correlation between WNT expression and 12 selected KRAS signature genes in cancer cell lines from various organs. Gray color indicates *p* > 0.05. Middle: Statistical significance of the differences in *WNT7B* expression between the high and low quartiles of the expression of the 12 KRAS signature genes in each organ’s cell line. Bottom: The average expression of *WNT7B* across all cell lines included in the analysis

Using MsigDB, we first identified a human hallmark gene set upregulated by KRAS activation, comprising 220 genes. In parallel, we extracted 512 differentially expressed genes (DEGs) that were upregulated in RZK organoids compared to RZ organoids, based on our sn multiome data. The intersection of these two gene sets yielded 33 overlapping genes, which we analyzed further (Supplementary Fig. 9a). We then examined a panel of 22 cancer types from various tissues, each represented by over 20 different cancer cell lines (Fig. 7a).

To assess the correlation between KRAS signature gene expression and *WNT7B* expression across cancer cell lines, we grouped cell lines from each tissue into quartiles based on the expression of the 33 KRAS signature genes. We then compared *WNT7B* expression between the highest (Q1, H) and lowest (Q4, L) quartiles and found that 20 out of the 33 KRAS signature genes positively correlated with *WNT7B* expression in at least 5 distinct cancer types (Fig. 7b and Supplementary Fig. 9b). This suggests that the KRAS-WNT7B axis is conserved across multiple organs. The top 4 genes, i.e. *ITGA2*, *TSPAN1*, *IGFBP3* and *PTGS2*, displayed a strong correlation with *WNT7B* expression in stomach cancer lines (Supplementary Fig. 9c).

To further validate our findings, we examined the expression patterns of these four genes using our sn multiome data (Fig. 3). All four genes were upregulated upon KRAS oncogenic mutation, while *IGFBP3* and *PTGS2* were specifically enriched in WNT7 + clusters (Supplementary Fig. 9 d). These results suggest that *IGFBP3* and *PTGS2* may serve as potential markers for cancers with an active KRAS-WNT7B axis.

Taken together, our results demonstrate that during homeostasis, gastric epithelial turnover is maintained by WNT2B secreted from *Robo2* high fibroblasts (Supplementary Fig. 9e). In human gastric tumorigenesis, *KRAS* activation, *HER2* overexpression, or *WNT* gene copy number gain can each drive epithelial WNT secretion, enabling niche escape. As revealed in our mouse models, we identified a mechanism by which epithelial WNT7B secretion is induced by the KRAS-MAPK axis and involves upregulation of TGFβRII expression and subsequent SMAD2/3 activation. Our results demonstrate molecular details of how gastric cancer achieves WNT self-sufficiency, i.e. through epithelial WNT production. Unlike colorectal cancer, in which *APC* and *CTNNB1* mutations constitutively activate WNT signaling, gastric cancer achieves WNT self-sufficiency through epithelial WNT secretion. This mechanism is accompanied by a specific vulnerability: inhibitors of WNT secretion should be further explored as a specific treatment avenue for gastric cancer, and potentially also for other cancers with an active KRAS-WNT7B axis.

## Materials and methods

### Mice

The *Villin-Cre*^*ERT2*^*; Rnf43*^*f/f*^*; Znrf3*^*f/f*^*, Rosa26-Cre*^*ERT2*^*; Red2-Kras*^*G12D*^*, and Villin-Cre*^*ERT2*^*; Rosa26-Confetti* mice were generated previously [55, 60]. The *Anxa10-Cre*^*ERT2*^ line [36], used for the gastric epithelium-specific conditional genetic mutation *Kras*^*lsl−G12D*^ (MGI ID: 008179), was obtained from The Jackson Laboratory and crossed with *Cdh1*^*f/f*^*; Tp53*^*R172H*^ mice provided by Daniel E. Stange to generate Ax10-CP and Ax10-CPK lines.

### Animal treatments

To activate *Anxa10-Cre*^*ERT2*^, age-matched mutant mice, together with wild-type negative control C57BL/6 J mice, were injected intraperitoneally with tamoxifen (Sigma), diluted in corn oil, at a dose of 2 mg (mg) per 20 g (g) of body weight 8–12 weeks of age. Both male and female mice were used for the experiments. One month after tamoxifen injection, mice were sacrificed by CO_2_ inhalation, and their stomachs were prepared for histological analysis and gland isolation. To induce Cre recombination in *Rosa26-Cre*^*ERT2*^ mice, tamoxifen (Sigma) was administered by intraperitoneal injection at a dose of 2 mg per 20 g body weight to mice aged 8–12 weeks. Forty-eight hours after injection, mice were euthanized by CO₂ inhalation, and the small intestine and pancreas were harvested for organoid establishment.

### Murine stomach preparation for histological analysis

Isolated stomach tissue was washed with cold phosphate-buffered saline (PBS) and cut longitudinally along the greater curvature, starting from the intestine. The sample was then spread and secured with needles on a piece of cardboard, then fixed in freshly prepared 4% paraformaldehyde (PFA) at 4 ℃ overnight (18 h) or in 10% neutral buffered formalin (NBF) at room temperature overnight with shaking. Fixed stomach tissue was washed 3 times with 1xPBS at 4 ℃ for 30 min.

### Paraffin embedding and immunohistochemistry of tissue sections

Stomach samples were dehydrated in an ethanol gradient of increasing concentration (70%, 80%, and 100%) for 80 min each. The dehydrated samples were then treated with xylene and infiltrated with paraffin in three rounds, each lasting 100 min, followed by embedding in a paraffin block and sectioning at a thickness of 2 mm for all histological analyses.

In preparation for immunohistochemistry and immunofluorescence, tissue sections were first rehydrated, and antigens were retrieved using sodium citrate (pH 6.0), following the protocols of the VBC or IBS Histology Facility.

For chromogenic immunohistochemistry Samples were first incubated in a blocking solution containing 3% H_2_O_2_ at room temperature for 10 min, followed by incubation in a blocking solution containing 2% bovine serum albumin (BSA), 5% goat serum, and 0.3% Triton-X100 in PBS at room temperature for 1 h. Recombinant anti-KI67 primary antibody (1:200; Abcam; ab16667) was applied to each tissue section, and detection was performed using a peroxidase-conjugated 2-step enhancer-polymer system (DCS, SuperVision 2 HRP Single Species). Hematoxylin and eosin staining was carried out without heat using the Epredia Gemini AS Automated Slide Stainer.

For immunohistochemistry of multiple antibodies, tissue sections were incubated in a blocking solution containing 5% dimethyl sulfoxide (DMSO), 2% normal donkey serum, and 0.5% Triton-X100 in PBS at room temperature for 1 h following antigen retrieval. Primary antibodies for GIF (Sigma, HPA040774, 1:200) and KI67 (Invitrogen, 14–5698-82, 1:200) were applied. Followed by secondary antibodies: donkey anti-Rabbit Alexa555 (Invitrogen, A32794, 1:500) and donkey anti-Rat Alexa 647 (Invitrogen, A48272, 1:500)). Neck cells were stained using a GS-II lectin conjugated to an Alexa 488 fluorophore (Invitrogen, L21415, 1:500) during the secondary antibody incubation step.

### *In situ* hybridization of tissue sections

*Wnt7b* and *Axin2* were detected with RNA *in situ* hybridization using the RNAScope Multiplex Fluorescent Detection Kit v2 according to the manufacturer’s protocol (ACDBio323110). Briefly, paraffin-embedded samples were freshly sectioned to 4 μm thickness prior to staining. On the day of staining, slides were manually pretreated to remove paraffin and retrieve target sequences, then stored overnight. Staining was performed according to the manufacturer’s protocol with probes for *Wnt7b* and *Axin2* (Advanced Cell Diagnostics, Cat No. 401131 and Cat No. 400331-C3, respectively). Stained slides were imaged using the Pannoramic FLASK 250 III scanner (3DHISTECH). Images were processed using CaseViewer software.

### Establishment and culture of mouse corpus epithelial gastric organoids

Mouse stomach glands were isolated as previously described [1, 8]. Briefly, freshly collected stomachs were washed with cold PBS, and the corpus region was separated. The corpus tissue was then cut into small pieces and incubated with Gentle Cell Dissociation Reagent (STEM Cell Technologies) for 10 min. Isolated glands were then seeded in Matrigel (Corning) at a density of 100–150 glands per well and cultured in basal medium—Advanced Dulbecco’s Modified Eagle Medium (DMEM)/F12 (Gibco) supplemented with 1% penicillin/streptomycin (pen/strep), 10 mM HEPES (Gibco), 1% GlutaMAX (Gibco), 1 × B27 (Life Technologies), and 1.25 mM N-acetylcysteine (Sigma-Aldrich)—together with a growth factor cocktail containing 50 ng/ml mouse epidermal growth factor (mEGF; Peprotech), 100 ng/ml mNoggin (Peprotech), 10% R-spondin1-conditioned medium or 100 ng/ml R-spondin (PeproTech), 50% Wnt3A-conditioned medium(Gradiant bioconvergence), 0.5 nM Surrogate WNT (IPA therapeutics), or 0.5ug/ml recombinant Human/Mouse Wnt-5a (Biotechne), 100 ng/ml human fibroblast growth factor 10 (hFGF-10; Peprotech), and 10 nM hGastrin (Sigma-Aldrich). During routine organoid maintenance, an Fzd7-specific WNT surrogate dimer [61](TZ-GF101, TheraZyne) was used as needed to replace Wnt3A-conditioned medium or Surrogate WNT, after thoroughly confirming that supports gastric organoid growth. It was not employed as an experimental treatment.

### Establishment and culture of mouse small intestinal organoids

Mouse small intestine organoids were isolated as previously described [62]. Briefly, freshly isolated small intestines were washed with cold PBS, gently scraped using a cover glass, then cut into small pieces and incubated with Gentle Cell Dissociation Reagent (STEM Cell Technologies) for 20 min. Isolated glands were seeded in Matrigel (Corning) at a density of 100–150 glands per well, then cultured in a basal medium—Advanced DMEM/F12 supplemented with 1% pen/strep, 10 mM HEPES (Gibco), 1% GlutaMAX (Gibco), 1 × B27 (Life Technologies), 1.25 mM N-acetylcysteine (Sigma-Aldrich), and 10 mM nicotinamide (Sigma-Aldrich)—along with a growth factor cocktail containing 50 ng/ml mEGF (Peprotech), 100 ng/ml mNoggin (Peprotech), 10% R-spondin1-conditioned medium or 100 ng/ml R-spondin (Peprotech),and 0.5 nM Surrogate WNT (IPA therapeutics).

### Establishment and culture of mouse pancreas ductal organoids

Mouse pancreas ductal organoids were isolated following Broutier et al [63]. Isolated organoids were cultured in a basal medium—Advanced DMEM)/F12 supplemented with 1% pen/strep, 10 mM HEPES (Gibco), 1% GlutaMAX (Gibco), 1 × B27 minus vitamin A (Life Technologies), 1 mM N-acetylcysteine (Sigma-Aldrich), and 10 mM Nicotineamide (Sigma-Aldrich)—along with a growth factor cocktail containing 50 ng/ml mEGF (Peprotech), 25 ng/ml mNoggin (Peprotech), 100 ng/ml R-spondin (PeproTech), 100 ng/ml hFGF10 (Peprotech), and 10 nM hGastrin (Sigma-Aldrich).

### Genotyping of mouse gastric organoids

Genomic DNA was extracted from mouse gastric organoids to genotype *Rnf43, Znrf3, Cdh1, Tp53*, and *Red2-Kras*^*G12D*^ alleles. Matrigel domes and culture medium were collected, and organoids were released from the matrix by gentle pipetting. The suspension was centrifuged at 600 × g for 5 min, and the supernatant was discarded. Pellets were washed once with PBS and centrifuged again under the same conditions. DNA extraction was performed using DirectPCR (Tail) reagent (VIAGEN) supplemented with proteinase K (Enzynomics). Samples were incubated at 60 °C overnight, followed by enzyme inactivation at 85 °C. Genotyping PCR was carried out using gene-specific primers (supplementary Table 2.) and GoTaq Flexi DNA polymerase (Promega), following the manufacturer’s instructions.

For validation of the *Red2-Kras*^*G12D*^ mutation, PCR amplicons were purified using the Expin PCR SV kit (GeneAll) and subjected to Sanger sequencing to confirm allele insertion.

### Mouse gastric organoid staining

Mouse gastric organoids were stained following Dekkers et al. [64]. Briefly, the culture medium was removed, and the organoids were washed with PBS. Cold Cell Recovery Solution (Corning) was then added and incubated until the Matrigel dissolved. Throughout the subsequent steps, pipette tips were pre-coated with 1% PBS/BSA to prevent adhesion. The organoids, now free of Matrigel, were collected into tubes using PBS, spun down, and the supernatant was removed. The organoid pellet was fixed with 4% PFA, subjected to permeabilization and blocking, and then immunolabeled with an anti-p-SMAD2/3 primary antibody (Invitrogen, PA5-110155, 1:50), anti-TGFBR2 primary antibody (Proteintech, 66636–1-IG, 1:200), Donkey anti-Rabbit Alexa555 secondary antibody (Invitrogen, A32794, 1:500) and Goat anti-Mouse Alexa 488 antibody (Invitrogen, A-11001, 1:500). Nuclei were stained with the reference dye 4′,6-diamidino-2-phenylindole (DAPI).

### Western blot from organoid

Organoids were lysed in RIPA buffer (50 mM Tris–HCl, pH 8.0, 150 mM NaCl, 1% Nonidet P-40, 0.5% sodium deoxycholate, 0.1% sodium dodecyl sulfate (SDS), supplemented with protease inhibitors (Roche) and phosphatase inhibitors (Roche)). Samples were loaded onto Bolt Bis–Tris Plus 4–12% gels (Invitrogen), and proteins were separated by electrophoresis at 200 V for 32 min at room temperature. The separated proteins were then transferred onto polyvinylidene fluoride (PVDF) membranes by electroblotting at 110 V for 1 h at 4 °C. Membranes were blocked with 5% bovine serum albumin (BSA) in Tris-buffered saline with 0.1% Tween-20 (TBST) (20 mM Tris, pH 7.5, 150 mM NaCl, 0.1% Tween-20) for 1 h at room temperature. Primary antibodies were diluted in blocking buffer and incubated with the membranes overnight at 4 °C. The following primary antibodies were used: anti-SMAD2/3 (Cell signaling technology, CST #8685, 1:1000), anti-p-SMAD2/3 (ThermoFisher, PA5-110,155, 1:1000), anti-ERK (Cell signaling technology, CST #9102, 1:1000), and anti-p-ERK (Cell signaling technology, CST #4370, 1:1000). After primary antibody incubation, the membranes were washed with TBST and incubated with the appropriate horseradish peroxidase (HRP)-conjugated secondary antibodies (anti-rabbit HRP (AB Frontier, LF-SA8002, 1:5000), anti-goat HRP (AB Frontier, LF-SA8012H, 1:5000)) for 1 h at room temperature. Protein bands were visualized using an enhanced chemiluminescence detection system and imaged with a 7500 Fast Real-Time PCR System. Band intensities were quantified using ImageJ software [65].

For membrane stripping, blots were first washed with TBST and then incubated in Restore Western Blotstripping buffer (ThermoFisher) at room temperature for 7–8 min. Following incubation, membranes were washed three times with TBST to ensure complete removal of residual antibodies. After confirming the absence of remaining signal, membranes were re-blocked and processed again starting from the blocking step as described above.

###  Induction of Confetti and *Red2Kras*^*G12D*^ in mouse organoids

Forty-eight hours after tamoxifen injection of *Rosa26-Cre*^*ERT2*^*; Red2Kras*^*G12D*^, the mouse was sacrificed by CO₂ inhalation. Then, small intestinal and pancreatic ductal organoids were established*. Villin-Cre*^*ERT2*^*; Rosa26-Confetti* mice were euthanized by CO₂ inhalation without prior tamoxifen treatment, and intestinal organoids were subsequently established. To induce Cre recombination in *Villin-Cre*^*ERT2*^ organoids, established cultures were treated with 1 μM 4-hydroxytamoxifen (Sigma) overnight. The following day, the medium was replaced with standard culture medium. At the splitting step, intestinal organoids were treated with prewarmed TrypLE (Gibco, 12,605–010) to separate RFP-expressing cells from YFP-expressing ones from the *Rosa26-Cre*^*ERT2*^*; Red2Kars*^*G12D*^ intestinal organoids. After 1–2 weeks of splitting, the RFP + intestinal organoids were collected using a 1000-L pipette under a bright field microscope. RFP intestinal organoids were cultured in Complete medium supplemented with 2 × Surrogate WNT (final concentration: 1 nM; IPA Therapeutics) and 5 × mNoggin (final concentration: 500 ng/ml; Peprotech). In the case of pancreatic organoids, a Wolf G2 Cell Sorter (Nanocellect) was used to separate YFP-expressing cells from RFP-expressing cells from *Rosa26-Cre*^*ERT2*^*; Red2Kras*^*G12D*^ pancreatic ductal organoids. The two cell types were then grown separately in Matrigel under the same media conditions.

### Separation of gastric glands and mesenchymal tissue

As illustrated in the schematic in Fig. 1c, we isolated the gastric epithelium and mesenchyme from mouse gastric organoids. Ten-week-old Wild type mice were anesthetized with sevoflurane (Sevoran, Abbott) and euthanized by cervical dislocation. The stomach was harvested and immediately rinsed in ice-cold chelating buffer containing 5.6 mM Na₂HPO₄ (Sigma-Aldrich), 8.0 mM KH₂PO₄ (Roth), 96.2 mM NaCl (Roth), 1.6 mM KCl (Sigma-Aldrich), 43.4 mM sucrose (OmiPlus), 54.9 mM D-sorbitol (Roth), and 0.5 mM DL-dithiothreitol (Roth), dissolved in 500 mL distilled water (Invitrogen), as previously described [66]. The stomach was opened along the greater curvature, and luminal contents were carefully washed away. The corpus region was dissected and minced into approximately 2 × 2 mm pieces using a scalpel. Tissue fragments were incubated for 2 h at room temperature in 10 mL chelating buffer supplemented with 10 mM EDTA (Invitrogen). Following incubation, tissue pieces were aligned in a Petri dish, excess liquid was removed using tissue paper, and the fragments were covered with a glass slide. Gland’s integrity was assessed under a stereomicroscope by identifying palisade-like structures. Gentle pressure was applied to the slide to release the gastric glands, which were then suspended in the remaining buffer. The slide was carefully removed and rinsed with ice-cold + + + medium composed of Advanced DMEM/F-12 (Gibco), 1% GlutaMAX™ (Gibco), 1% HEPES 1 M (Gibco), and 1% penicillin–streptomycin (Gibco). The + + + medium containing released glands and tissue fragments was transferred to a 15-mL tube (Greiner Bio-One™) and allowed to settle for 5 min. The supernatant was carefully transferred to a fresh tube without disturbing the sedimented tissue. The supernatant was centrifuged at 200 × g for 5 min at 4 °C to collect epithelial cells. Residual tissue fragments in the original tube were transferred to a Petri dish and mechanically cleared of remaining epithelial tissue by gentle scraping with a scalpel. Using two fine forceps, the white mesenchymal tissue was then separated from the underlying muscle layers..

### WNT retrieval using retroviral WNT overexpression in mouse gastric organoids

#### Retrovirus production

The retroviral infection system was used as previously described [34]. PlatinumE cells (a kind gift from Hans Clevers, Hubrecht Institute, Netherlands) were used to package and produce virus. Cells were thawed, washed twice in DMEM/F12 with 10% heat-inactivated fetal calf serum (FCS) and 1% pen/strep (+ + medium), and centrifuged at 500 × g for 5 min between each washing step. The pellet was resuspended in 5 ml of + + medium and plated in a 25 cm^2^ cell culture flask (Corning). For selection, puromycin (1 µg/µl) and blasticidin (10 µg/µl) were added, and cells were incubated at 37 °C with 95% humidity and 5% CO_2_. Medium was replaced every 3 days with fresh antibiotics. For passaging, cells were washed twice with PBS, then detached by incubating in trypsin for 5 min at 37 °C. Trypsinization was stopped by + + medium addition. After centrifugation at 500 × g for 5 min, cells were either seeded for virus production or transferred to a bigger flask. For virus production, 0.8 × 10^7^ cells were seeded in 15 cm Petri dishes with 25 ml of + + medium without antibiotics. Cells were transfected using 30 µg of pMSCV-loxP-dsRed-loxP-eGFP-Puro-WPRE (Addgene, Plasmid #32,702) or pMSCV-loxP-dsRed-loxP-Cited2-3HA-Puro-WPRE (Addgene, Plasmid #32,703). DNA was mixed with Lipofectamine 2000 transfection reagent (ThermoFisher) to a total volume of 250 µl and incubated for 30 min at room temperature, following which 250 µl of Opti-MEM medium (ThermoFisher) was further added. This transfection mix was then carefully added to the Petri dishes containing PlatinumE cells. One day post-transfection, cells were checked for successful transfection through the detection of red fluorescence. Two days later, the virus-containing supernatant was collected, filtered through a 0.45 µm filter, and centrifuged overnight at 8000 × g at 4 °C. The next morning, the supernatant was discarded, and the pellet was resuspended in 20 µl of infection medium. The infection medium consisted of mouse gastric organoid culture medium without pen/strep, but with primocin and 1:1000 polybrene (Sigma-Aldrich). Unused viral medium was stored at −80 °C.

#### Retroviral infection of murine gastric organoids

Gastric organoids from the corpus region of *Rosa26-Cre*^*ERT2*^ mice were used for infection. Eight wells of a 48-well plate were pooled into a 15 ml tube and mechanically dissociated as described above for normal passaging. Cells were centrifuged at 300 × g and 4 °C for 5 min, the pellet was resuspended in 500 µl of Cell Recovery Solution (Corning), and kept on ice for 10 min. Cells were then washed with 10 ml of PBS, and centrifuged at 500 × g and 4 °C for 5 min. The pellet was resuspended in 2 ml of TrypLE (Gibco) and incubated at 37 °C for 2 min. The reaction was stopped by adding 2 ml of infection medium and distributed in 3 × 15 ml tubes. The suspensions were then centrifuged at 500 × g and 4 °C for 5 min. In the meantime, the virus pellets (Cited2 and eGFP) were resuspended in 250 µl of infection medium. Pellets were then resuspended in the corresponding virus solution or pure infection medium as a control. Cell suspensions were plated on a 48-well plate, sealed with parafilm, and spinoculated at 600 × g and 32 °C for 60 min. After spinoculation, the parafilm was removed and the plate was incubated at 37 °C with 95% humidity and 5% CO_2_ for 6 h. The content of each well was then transferred to a 15 ml tube, 1750 µl of infection medium was added, and the tubes were centrifuged at 300 × g and 4 °C for 5 min. The supernatant was discarded, and the pellet was resuspended in 20 µl of Matrigel and plated in one well. After 15 min of incubation, 250 µl of normal murine gastric organoid medium was added. Three days post-infection, organoids were checked for red fluorescence under the microscope. Upon detection of red signals, puromycin selection was started. Puromycin was added at a concentration of 2 µg/ml and selection performed until the uninfected control organoids were dead. Once selection was completed, organoids were induced using 5 µM hydroxy-tamoxifen (4-OHT; Sigma-Aldrich) in mouse gastric organoid medium. Tamoxifen induction was performed for 3 days, with the medium and 4-OHT changed daily.

### RNA purification and quantitative reverse transcription polymerase chain reaction (qRT-PCR)

To assess the expression of gene of interest in mouse and human gastric organoids, total RNA was isolated using the RNeasy Mini Kit (Qiagen) following the manufacturer’s protocol. Between 300 ng and 1 µg of total RNA, depending on sample concentration, was used for cDNA synthesis with the SuperScript™ IV First-Strand Synthesis System (Invitrogen). Quantitative real-time PCR was performed using KAPA SYBR® FAST (Roche) on a 7500 fast Real-Time PCR System (Applied Biosystems) according to the manufacturer’s instructions. Relative mRNA expression levels were normalized to *Gapdh*. Primer sequences are provided in Supplementary Table 1.

### Establishment of human gastric cancer patient-derived organoids (PDOs)

Human gastric cancer PDOs were established from tissues obtained from ascites, endoscopic or surgical procedures with informed consent and ethics permission from Yonsei University College of Medicine, Seoul, South Korea or the Department of Visceral, Thoracic and Vascular Surgery at the University Hospital Carl Gustav Carus of TU Dresden, Germany. Organoids were generated as described previously [66]. Organoids were maintained in the following culture medium: Advanced DMEM/F12 with 1% pen/strep, 10 mM HEPES (Gibco), 1X GlutaMAX (Gibco), 50% Wnt3A conditioned medium (Gradiant bioconvergence) or 0.5 nM Surrogate WNT (IPA therapeutics), 10% R-spondin1 conditioned medium or 100 ng/ml R-spondin(PeproTech), 100 ng/ml hNoggin (Peprotech), 1 × B27 (Gibco), 1.25 mM N-acetyl-L-cysteine (Sigma-Aldrich), 200 ng/ml hFGF10 (Preprotech), 50 ng/ml mEGF (Peprotech), 1 nM hGastrin (Sigma-Aldrich), 2 μM A83-01 (Tocris) and 0.5 μM PGE2 (Tocris).

HER2 activity was determined by the Pathology Department of the Yonsei University following an established guideline [56]. The expression of HER2 was confirmed using immunohistochemistry in organoids and parent cancer tissues.

### Growth factor withdrawal experiments in gastric corpus organoids

All organoids were maintained in complete medium and passaged at a 1:5 ratio on day 0. Individual wells were supplied with either complete medium or selection medium deficient in R-spondin1, WNT, mEGF, or hFGF. The medium was changed every other day, and organoids were passaged every week until the end of the experiment.

All organoids were maintained in complete medium prior to drug treatment. On day 0, organoids were passaged and maintained in selection medium containing 10 μM Wnt-C59 (Selleckchem), 2 μM A83-01 (Tocris), 50 μM T-5224 (MedChem Express), 10 μM LY294002(MedChem Express), or 5 μM PD0325901(Axon), 5uM ITD (Sigma), or 5uM LY2109761 (Sigma) until the end of the experiment. The medium was refreshed every two days, and organoids were passaged every week.

### Growth factor withdrawal experiments in Kras mutant- and HER2 3 + PDOs

Three KRAS mutant PDOs and twelve HER2 3 + PDOs were cultured in WNT-free normal culture medium supplemented with C59 (Selleckchem) and/or 100 nM WNT (IPA therapeutics). The culture medium was changed every 3 days. Organoids were passaged at a 1:3 ratio every 8–9 days, when the Matrigel dome containing the organoids reached ~ 90% confluency. Following passage, 10 uM ROCK inhibitor (Tocris) was added.

### CRISPR-mediated Tp53 targeting in gastric corpus organoid

Gastric corpus organoids were established from *Anxa10-Cre*^*ERT2*^*-Cdh1*^*f/f*^*-Kras*^*G12D*^ mice and the *Tp53* allele was targeted by co-transfection with sgRNA (GTGTAATAGCTCCTGCATGGGGG) together with the Cas9 expressing vector using a NEPA21 electroporator. Successfully targeted organoids were selected by treatment with 1 mM Nutlin-3 (Selleck Chem) for 2 weeks, with weekly passages.

###  Targeting *hKRAS*^*G12S*^ in human gastric organoid

*KRAS* alleles were targeted using two plasmids. For this purpose, two sgRNA sequences for KRAS, as well as an sgRNA sequence to linearize the repair template plasmid, were inserted into the px458_Conc3 plasmid (Addgene Plasmid #134,450) using Golden Gate Cloning, as described by Andersson-Rolf et al., [67]. The generated plasmid, together with a repair template plasmid introducing the G12S point mutation, was transfected by electroporation as described by Gaebler et al., [68]. In addition to the G12S point mutation, the repair template also contained several silent mutations designed to prevent the sgRNAs from re-binding. Successfully transfected organoids were selected by EGF deprivation for 4 weeks, with weekly passaging. The integration of the repair template at the correct position within the *KRAS* gene was confirmed by PCR using Q5 polymerase. The PCR primers were designed such that one primer binds outside the repair template, while the other binds over the template’s silent mutations. Primer sequences are listed below.

**Table:** 

Oligonucleotide	Sequence (5’—3’)	Function
sg_hKRAS_C1_A	CACCGGAATGACTGAATATAAACTTGGT (28nt)	1 st sgRNA sequence for hKRAS
sg_hKRAS_C1_B	TAAAACCAAGTTTATATTCAGTCATTCC (28nt)	1 st sgRNA sequence for hKRAS
sg_hKRAS_C2_a	ACCGGGTAGTTGGAGCTGGTGGCGTG (26nt)	2nd sgRNA sequence for hKRAS
sg_hKRAS_C2_b	AAAACACGCCACCAGCTCCAACTACC (26nt)	2nd sgRNA sequence for hKRAS
sgPITCH_C3_b	AAACAACACGTACGCGTACGATGC (24nt)	sgRNA sequence to linearize the repair template
sgPITCH_C3_a	CCGGGCATCGTACGCGTACGTGTT (24nt)	sgRNA sequence to linearize the repair template
hKRAS_5’_fwd	TTGTGAGGGTGTGCTACAGG (20nt)	PCR primer in KRAS gene
hKRAS_5’_rev	GCCAACTACCACGAGCTTG (19nt)	PCR primer in KRAS Repair Template
hKRAS_3’_fwd	TGACAGAATACAAGCTCGTGG (21nt)	PCR primer in KRAS Repair Template
hKRAS_3’_rev	ATGGACCCTGACATACTCCC (20nt)	PCR primer in KRAS gene

### Single-nucleus (sn) multiome(RNA + ATAC) sample preparation from mouse stomach

Mouse gastric corpus nuclei were isolated from a minimum of 2–3 mice per sample to minimize individual variability. Dissected corpus tissue was minced in 1 mL of lysis buffer, then transferred to a homogenizer tube, and homogenized 50 times on ice. After adding 1 ml of lysis buffer, the sample was incubated on ice for 5 min with intermittent pipette mixing using a wide-bore tip. The suspension was filtered through a 70 μm strainer, and the flow-through was centrifuged at 500 × g and 4 °C for 5 min. The pellet was resuspended in PBS (1% BSA + 1 U/μL RNase inhibitor), incubated briefly on ice, and centrifuged at 500 × g and 4 °C for 5 min. The final pellet was resuspended in PBS (1% BSA + 1 U/μL RNase inhibitor). Isolated nuclei were subsequently used for sn multiome sequencing, following the standard protocols for 10X Genomics Chromium Next GEM Single Cell Multiome ATAC + Gene Expression.

### sn multiome sample preparation from mouse organoids

Mouse gastric corpus organoids were maintained in complete medium (wild-type) or R-spondin-deficient medium (Ax10-RZ and Ax10-RZK) until 10 days prior to the experiment. The culture medium was then replaced by complete medium for all samples and maintained for one week. Organoids were passaged 3 days before the experiment. For single-cell dissociation, mechanically disrupted organoids were incubated with TrypLE (Gibco) at 37℃ for 30 min, following which nuclei were extracted. Libraries for each sample were prepared and sequenced by the Vienna BioCenter Core Facilities or macrogen using an Illumina NovaSeq system.

### sn multiome data processing of stomach tissue

Stomach tissue snMultiome data were processed using the Seurat (v4.1.0) pipeline [69]. Raw and filtered matrices were obtained from the Cell Ranger ARC (v2.1.0) output using ‘Read10X_h5′ [69]. Ambient RNA contamination was corrected using SoupX (v1.6.2) [70], and the corrected RNA count matrix was used to construct the Seurat object for downstream analysis. We filtered out low-quality nuclei with nFeature_RNA less than 300 or greater than 3,000, total UMI counts exceeding 10,000, or mitochondrial RNA fraction above 5%. Using the selected nuclei, we sequentially conducted ‘NormalizeData’ (log-normalization), ‘FindVariableFeatures’ (‘vst’ method, 2,000 features), ‘ScaleData’, ‘RunPCA’ with 30 principal components (PCs), ‘RunUMAP’ using the PCA reduction, and ‘FindNeighbors’. Clustering was performed using the Louvain algorithm with a resolution of 0.9. Doublets were identified and removed using the DoubletFinder (v2.0.6) [71] R package with default settings.

### sn multiome data processing of organoid

To generate count matrices, consisting of both gene expression and chromatin accessibility peaks for each organoid sample—such as wild type (WT), Rnf43/Znrf3 double knock-out (RZ), and Kras activated RZ-DKO (RZK)— cellranger-ARC count (v6.1.1) [72] was utilized with default option and mouse reference (‘refdata-cellranger-arc-mm10-2020-A-2.0.0’) provided by 10 × Genomics. Based on both Signac (v1.8.0) [73] and Seurat (v4.1.0) [69]pipelines, analytic objects from ‘filtered_feature_bc_matrix’ and ‘atac_fragments.tsv.gz’ of Cell Ranger were generated, using the mouse genome annotations from ‘GetGRangesFromEnsDb’ with ‘EnsDb.Mmusculus.79’. We filtered out poor-quality cell nuclei with unique molecular identifiers (UMIs) for RNA less than 1,000 and more than 25,000, and for ATAC less than 1,000 and more than 100,000, percent mitochondrial genes more than 15%, and nucleosome signal more than 2 for those three samples. Using the selected nulei, we sequentially conducted NormalizeData (log-normalization) and FindVariableFeatures (‘vst’ method, 2,000 features) functions for RNA expression profiles. We also implemented FindTopFeatures with min.cutoff is ‘q0’, RunTFIDF, and RunSVD for both ATAC and macs2 assays for peak profiles from Signac package. After preprocessing individual objects from RZ and RZK samples, we performed SCTransform for RNA expression profile to prepare integration for the objects. The listed objects were integrated with the sequential functions such as ‘SelectIntegrateionFeatures’, ‘PrepSCTIntegration’, ‘FindIntegrationAnchors’, and ‘IntegrateData’. To generate cluster map for the integrated object, we performed ‘ScaleData’, ‘RunPCA’, ‘RunUMAP’, ‘FindNeighbors’, and FindClusters (‘resolution’ is 0.75, Louvain algorithm) with 30 PCs. We also performed joint integration of WT, RZ, and RZK samples using a similar workflow. For this dataset, RunUMAP’ was performed using the neighbor graph generated by ‘FindNeighbors’ and a clustering resolution of 1.2 was applied in ‘FindClusters’ function.

### sn multiome data analysis

To annotate cell types in the dataset, specific markers were used, such as *Muc6* and *Cftr* for neck cells; *Lgr5, Ero1l*,and *Ddit4* for SPEM1 cells; *Glipr1* and *CD44* for SPEM2; *Wnt7b, Porcn* for Wnt7 + cells; *Mki67, Foxm1, Hmgb2, Top2a,* and *Smc2* for proliferating cells; *Gkn1* for pre-pit cells; and *Muc5ac*, *Tff1,* and *Gkn2* for pit cells. The ‘DotPlot’ function from the Seurat package was used to generate Fig. 3b, and the ‘dittoBarPlot’ function from the dittoSeq R package (v1.6.0) [74] was used to generate Fig. 3c, with the corresponding cell type markers and annotation. In Fig. 3e, Wnt7b-expressing cells were defined as those with a non-zero UMI count for Wnt7b in the RNA count-matrices. To identify differentially expressed genes, we used the ‘FindMarkers’ function in Seurat with likelihood-ratio (LR) testing. Only genes detected in at least 5% of cells and with a log2(fold-change) > 0.1 were obtained. To account for variation in chromatin accessibility, the number of ATAC fragments per cell was included as a latent variable (latent.vars = “atac_peak_region_fragments”). Motif enrichment analysis was performed using Signac (v1.8.0) with position frequency matrices (PFMs) obtained from the JASPAR2020 CORE vertebrate collection [75]. Differentially accessible peaks (DAPs) were identified from the MACS2-called peak set [76], and peaks with adjusted *p*-value < 0.05 were used for downstream analysis. The nearest gene to each peak was annotated using the ‘ClosestFeature’ function. For motif analysis, PFMs were loaded using ‘getMatrixSet’ and motifs were added to the chromatin object with ‘AddMotifs’ using the reference genome. Motif enrichment was evaluated using ‘FindMotifs’ on DAPs with adjusted *p*-value < 0.005. Motifs with motif-level adjusted *p*-value < 0.05 were considered significantly enriched, and enriched motifs were ranked by -log(adjusted p-value) for visualization [77, 78].

### Trajectory inference

Trajectory inference was performed using Monocle3 (v1.3.7) [77]. The UMI count matrix and cell metadata from the Seurat object were used to construct a Monocle3 object using the ‘new_cell_data_set’ function. A proliferating cluster is enriched for markers that are highly expressed during the proliferative phase, regardless of a cell’s eventual differentiation fate. As a result, cells from different lineages can cluster together due to shared cell-cycle activity rather than true lineage similarity. To avoid this confounding effect and to clearly define the trajectory root, the proliferating cluster was excluded from the analysis. This approach is consistent with previous single-cell lineage studies in which proliferating clusters were removed to prevent cell-cycle-driven artifacts. Trajectories were inferred across the remaining clusters using default parameters. The ‘align_cds’ function of Monocle3 applied the mutual nearest neighbor (MNN) [78] tool for batch correction and data integration between the RZ and RZK samples. Data dimensionality was further reduced using uniform manifold approximation and projection with 100 calculated PCs. Clustering was performed using the Leiden algorithm [79] with the ‘cluster_cells’ function at a resolution of 1e-3.

### Targeted panel sequencing and copy number analysis

Targeted sequencing was performed using the TruSight Oncology 500 Panel (Illumina, San Diego, CA, USA), following the manufacturer’s protocol. A total of 120 ng of DNA from each organoid sample was used for library preparation. Barcoded libraries were sequenced (2 × 150 bp paired-end) on an Illumina NextSeq500 platform. Raw sequencing data were processed using the TSO500 Local App (v2.2.0.2) in a Docker environment, with GRCh37 as the reference genome and default parameters. Copy-number variation was detected using CRAFT (v1.0.0.52). Copy-number fold change was calculated by normalizing gene-level sequencing coverage against a baseline derived from CRAFT-derived internal panel of normals.

### TCGA data acquisition and processing

Publicly available molecular and clinical data from TCGA Stomach Adenocarcinoma (TCGA-STAD) were obtained. The dataset had been processed by the Genaomic Data Commons (GDC) and released in July 2025. All files were downloaded via cBioPorta [80]. The dataset provides mutation calls, gene-level copy number alterations (CNA), transcriptome expression profiles, and clinical annotations. Samples were included in downstream analyses only if all data types (mutation, CNA, and RNA-seq) were available. For mutation data, only variants listed as oncogenic or likely oncogenic in OncoKB [81] or Cancer Hotspots [82, 83] were considered as driver alterations. CNA profiles were provided in GISTIC 2.0 [84] format, with a value of + 2 classified as amplification. RNA-seq expression data were downloaded as TPM values and converted to log2(TPM + 1) for downstream analyses. Clinical metadata, including AJCC pathological stage, were incorporated into downstream analysis. For pathological stage-based analyses, samples were grouped into 4 major stage categories (Stages I–IV) to ensure adequate statistical power. Mutual exclusivity among gene alterations was evaluated using one-sided Fisher’s exact test, and effect sizes were quantified using odds ratios. For comparisons of gene expression between group, a one-sided Mann–Whitney U test was used.

### Copy number variation analysis

Whole-genome sequencing (WGS) and whole-exome sequencing (WES) data was analyzed using the nf-core/sarek pipeline (v3.4.0) [85] with default parameters. The quality of input FASTQ files was evaluated with using FastQC (v0.12.1) (https://www.bioinformatics. babraham.ac.uk/projects/fastqc/), and adapters were trimmed using FastP (v0.23.4) [86]. Reads were aligned to the human reference genome (GRCh37) using BWA MEM (v0.7.17-r1188) [87]. Duplicate reads in the aligned BAM files were processed with GATK4 MarkDuplicates following which base quality was recalibrated using GATK4 BaseRecalibrator and ApplyBQSR (v4.4.0.0) [88]. Gene copy numbers were detected using CNVkit (v0.9.11) [89], and gene copy number ratios (cnr) were estimated using pooled normal reference constructed separately for datasets from three different research groups. Copy numbers were visualized using the heatmap function of the seaborn python package (v0.13.2) [90].

### Bulk-RNA seq analysis

RNA-seq data were processed using the nf-core/rnaseq pipeline (v3.12.0) [91] with default parameters. The quality of the input FASTQ files were assessed using FastQC (v0.11.9) (https://www.bioinformatics.babraham.ac.uk/projects/fastqc/) and adapters were trimmed using TrimGalore! (v0.6.7) [92]. Reads were aligned to the human reference genome (GRCh37) using STAR (v2.6.1d) [93], and the aligned BAM files were quantified using Salmon (v1.10.1) [94]. Transcript-level were imported and abundances, counts, and transcript lengths were summarized at the gene level using the tximport R package (v1.30.0) [95]. Subsequently, the raw counts were normalized using the TMM (trimmed mean of M values) method implemented in the edgeR R package (v4.0.16) [96]. Publicly available RNA-seq data from adjacent normal tissue or gastric cancer samples were incorporated from the GSE122401(1)(1) dataset [97]. Among adjacent normal and tumor samples, only those with GEO accession numbers were included, resulting in 79 normal and 80 tumor samples. Raw FASTQ files downloaded from the GEO database [98] and processed using the same pipeline to ensure a consistent analysis. The ‘stripplot’ and ‘boxplot’ functions of the seaborn python package (v0.13.2) [90] were used for visualization purposes. Statistical analysis was performed using a sample Statistical analysis was performed using a one sample Wilcoxon signed rank test (scipy v1.13.0) [99] to determine whether gene expression in GC-PDOs analyzed in this study was significantly higher than in public data.

### Pathway activity estimation

MAPK pathway activity was inferred at the single-cell level using PROGENy [100], which is implemented in Decoupler package (v2.1.1) [101]. Pathway scores were computed for each cell using the “decoupler.run_ulm” function, which applies a univariate linear model. To mitigate the effects of sparsity inherent in single-cell RNA-seq data, ten pseudo-bulk samples for each genotype were generated by aggregating 500 randomly selected cells per pseudo-bulk sample. PROGENy-derived MAPK gene sets (the top 100, 500 and 1,000 genes, weighted both positively and negatively) were used to assess pathway enrichment. Single-sample gene set enrichment analysis (ssGSEA) was performed for each pseudo-bulk sample using the “decoupler.run_gsea” function (tmins = 5, times = 10,000). Statistical significance was evaluated using a one-sided Wilcoxon rank-sum test with the “scipy.stats.ranksums” function in the SciPy package (v1.16.0) [99].

### Statistical analysis

qRT-PCR experiments were performed with biological triplicates or more, and each set was analyzed by paired t-test. For comparisons involving more than two groups, analysis of variance (ANOVA) was performed using Prism software (Graphpad). Organoid quantification was analyzed using unpaired t-tests. Alternative statistical methods, when used, are clarified in the figure legends. Statistical significance was set at a threshold p-value < 0.05.

### WNT ligand expression in mouse stomach

To infer WNT ligand expression, the processed h5ad file containing sn transcriptomic data from mouse stomach (GSE247719) was downloaded, and log10-scaled normalized counts were used [32]. Data analysis was performed following the procedures described in the reference study.

### Cancer Cell line WNT7B correlation analysis

The KRAS signature gene set was established by overlapping DEGs from the comparison between RZ and RZK organoids, accounting for the effect of the KRAS^G12D^ mutation, with known genes of the KRAS signaling pathway from public databases. DEGs were identified by using ‘FindMarkers’ function in Seurat R package (v4.1.0) and MAST test (v1.24.1) [102] with an adjusted *p*-value < 0.01 and log_2_(fold-change) > 1 from transcriptomic profiles of RZ and RZK organoids snMultiome data. Mouse DEGs were subsequently mapped to their human orthologs using the ‘gconvert’ function in gprofiler2 R package (v0.23) [103]. KRAS signaling pathway genes were derived from the ‘HALLMARK_KRAS_SIGNALING_UP’ gene set in MSigDB using the msigdbr R package (v7.5.1) [104]. By intersecting the mentioned gene sets, 33 KRAS signature genes were identified. Batch-corrected (ComBat) [105] count matrices with log2(transcripts per millions + 1) values derived from ‘DepMap Public 24Q4 version’ [106] containing data for individual cancer cell lines, were used to calculate expression correlations between WNT7B and KRAS signature genes across tissues. Cancer cell lines were then grouped by tissue type, and Spearman correlations between the expression of WNT7B and each KRAS signature gene were calculated for individual tissues. Heatmap clustering was performed by calculating distances using the ‘dist’ function with the binary method, followed by hierarchical clustering using the ‘hclust’ function with the average linkage method. The binary matrix for clustering consisted of 0 (non-significant) and 1 (p < 0.05), determined based on correlation p-values. The dendrogram was visualized using the dendextend R package (v1.19.0). Although the correlation coefficient is high, the cell lines within tissues may exhibit low levels of WNT7B expression. To illustrate the expression trends of WNT7B, mean expression levels were calculated for individual tissues. To assess statistical significance, a one-sided Wilcoxon rank-sum test was used to compare WNT7B expression fold changes between the first (Q1) and fourth (Q4) quartiles, based on KRAS signature gene expression levels.

## Supplementary Information

Supplementary Material 1: Supplementary Table 1 - 3.

Supplementary Material 2: Supplementary Figure 1 - 9.

Supplementary Material 3: Raw images of gels and membranes used in Supplementary figures.

## Data Availability

All original computer code used in this manuscript is available at our GitHub repository (https://github.com/BIS-lab/Gastric_cancer_WNT). This repository contains scripts for preprocessing, integration, and downstream analysis of single-cell multiome data, cancer cell line expression profiles (from public datasets such as DepMap), and bulk RNA-seq and WGS/WXS data. Processed single-cell multiome data are deposited under GEO accession number: GSE229124 (https://www.ncbi.nlm.nih.gov/geo/query/acc.cgi?acc=GSE229124). Raw bulk RNA-seq, WGS, and WXS data are available via NCBI SRA: (https://dataview.ncbi.nlm.nih.gov/object/PRJNA953002?reviewer=j0mol7gc1iie4goh1kik2e8qdi). All resources are accessible without login or personal information.
