# Supplementary material for: Epithelial WNT secretion drives niche escape of developing gastric cancer
Source: Mol Cancer. 2025 Dec 16;25:1. doi: 10.1186/s12943-025-02543-z (PMC12766950; doi:10.1186/s12943-025-02543-z)
Supplement: Supplementary file 1 — Supplementary Material 1: Supplementary Table 1 - 3. [file 12943_2025_2543_MOESM1_ESM.docx]

| **Figure 1 qRT-PCR primer** | | | |
| --- | --- | --- | --- |
| Gene | Forward primer | | Reverse primer |
| Wnt2b | GTCCTGGTGGTACATAGGG | | GTAGCGTTGACACAACTGC |
| Wnt4 | GAGCAATTGGCTGTACCTG | | CAGGCCTTTGAGTTTCTCG |
| Wnt5a | TAAGGGTTCCTATGAGAGCG | | GCCAGGTTGTATACTGTCCT |
| Wnt5b | ACACCAGTTTCGACAGAGG | | TCTCGGCTACCTATCTGCA |
| Barx1 | CCTGACAGAATAGATCTAGCTG | | TCTTCCATTTCATCCTCCGA |
| Pgc | CTCAAGGGCAGACCTTCTC | | TGCTTTGGACTCTCAGAGTG |
| Gapdh | AGCTTGTCATCAACGGGAAG | | CGGAGATGATGACCCTTTTG |
| **Figure 2-5 qRT-PCR primer** | | | |
| Gene | | Forward primer | Reverse primer |
| Wnt2b | CTGCTGCTGCTACTCCTGACT | | ATGTCTGGGTAGCGTTGACAC |
| wnt4 | GAGCAATTGGCTGTACCTG | | CAGGCCTTTGAGTTTCTCG |
| wnt5a | GTCTTCCAAGTTCTTCCTAATG | | ACAGGGTTATTCATACCTAGAG |
| wnt5b | GCATTGGGATGGGTTGA | | TAGTGACCACCAGGAGTT |
| wnt7a | TCGGGAAGGAGCTCAA | | CAGCAGTGATGGCATGG |
| wnt7b | GCAGAAAGGTTCTGGAGGA | | CTTAGGTAGCGTGGTCCAG |
| Axin2 | TAGGTTCCGGCTATGTCTTTG | | TGTTTCTTACTCCCCATGCG |
| CyclinD1 | AGTGCGTGCAGAAGGAGATT | | CACAACTTCTCGGCAGTCAA |
| Gapdh | CATGGCCTTCCGTGTTCCTA | | CCTGTTCACCACCTTCTTGA |
| **Figure S5H qRT-PCR primer** | | | |
| Gene | Forward Primer | | Reverse Primer |
| Wnt7a | GACAAATACAACGAGGCCGT | | GGCTGTCTTATTGCAGGCTC |
| Wnt7b | ACAGGAGGGTGGGGATAGA | | GAAACAGCCCAGGAAACCGT |
| Actin | ATATCGCTGCGCTGGTCGTC | | AGGATGGCGTGAGGGAGAGC |

**Table 1. List of qRT-PCR primers used in this study**

| **Genotyping primer** | | | |
| --- | --- | --- | --- |
| Gene | Primer name | Primer sequence | Product Size (bp) |
| Cdh1 | Fwd_ds | GGGTCTCACCGTAGTCCTCA | Wt: 243bp |
|  | Rev_ds | GATCTTTGGGAGAGCAGTCG | Mut: 310bp |
| P53 | Tp53[tm2Tyj] _WT_F | AGGTGTGGCTTCTGGCTTC | Wt 370bp |
|  | Tp53[tm2Tyj] _Com_R | GAAACTTTTCACAAGAACCAGATCA | Mut 174bp |
|  | Tp53[tm2Tyj] _Mut_F | CCATGGCTTGAGTAAGTCTGCA |  |
| Kras (G12D) | Kras_Ki_F | CTAGCCACCATGGCTTGAGT | Wt: 450bp |
|  | Kras_WT_F | TCCGAATTCAGTGACTACAGATG | Fl: 320bp |
|  | Kras_WT_R | ATGTCTTTCCCCAGCACAGT |  |
| RNF43 | NeoL | ATTGGCATCGCAATTTTCTAA | WT: 102bp |
|  | NeoR | TCGGTGTTCTTAACTGCTGA | Mut: 207bp |
| ZNRF | NeoL | GCAGTAATAAACAGGTAAAGAGCAAA | WT: 246bp |
|  | NeoR | GCTGGAGAGACCGTTCATAAG | Mut: 324bp |

**Table 2. List of genotyping primers used in this study**

| **Wnt7b** |  |  |  |  |  |  |  |  |  |  |  |
| --- | --- | --- | --- | --- | --- | --- | --- | --- | --- | --- | --- |
|  | **Class 0** | **rest** | **total** |  | **Class 0** | **rest** | **total** |  | **Class 0** | **rest** | **total** |
| **WT** | 41 | 19 | 60 | **RZ** | 3 | 57 | 60 | **WT** | 41 | 19 | 60 |
| **RZ** | 3 | 57 | 60 | **RZK** | 0 | 61 | 61 | **RZK** | 0 | 61 | 61 |
| **total** | 44 | 76 | 120 | **total** | 3 | 118 | 121 | **total** | 41 | 80 | 121 |
| **p-value** |  |  | **5.24279E-14** | **p-value** |  |  | **0.1188277** | **p-value** |  |  | **6.0482E-18** |
| **Significance** |  |  | ******** | **Significance** |  |  | **ns** | **Significance** |  |  | ******** |
|  | **Class 1** | **rest** | **total** |  | **Class 1** | **rest** | **total** |  | **Class 1** | **rest** | **total** |
| **WT** | 18 | 42 | 60 | **RZ** | 22 | 38 | 60 | **WT** | 18 | 42 | 60 |
| **RZ** | 22 | 38 | 60 | **RZK** | 7 | 54 | 61 | **RZK** | 7 | 54 | 61 |
| **total** | 40 | 80 | 120 | **total** | 29 | 92 | 121 | **total** | 25 | 96 | 121 |
| **p-value** |  |  | **0.114293714** | **p-value** |  |  | **0.00083901** | **p-value** |  |  | **0.00766918** |
| **Significance** |  |  | **ns** | **Significance** |  |  | ******** | **Significance** |  |  | ******* |
|  | **Class 2** | **rest** | **total** |  | **Class 2** | **rest** | **total** |  | **Class 2** | **rest** | **total** |
| **WT** | 1 | 59 | 60 | **RZ** | 26 | 34 | 60 | **WT** | 1 | 59 | 60 |
| **RZ** | 26 | 34 | 60 | **RZK** | 20 | 41 | 61 | **RZK** | 20 | 41 | 61 |
| **total** | 27 | 93 | 120 | **total** | 46 | 75 | 121 | **total** | 21 | 100 | 121 |
| **p-value** |  |  | **7.8955E-09** | **p-value** |  |  | **0.07350968** | **p-value** |  |  | **2.2043E-06** |
| **Significance** |  |  | ******** | **Significance** |  |  | **ns** | **Significance** |  |  | ******** |
|  | **Class 3** | **rest** | **total** |  | **Class 3** | **rest** | **total** |  | **Class 3** | **rest** | **total** |
| **WT** | 0 | 60 | 60 | **RZ** | 9 | 51 | 60 | **WT** | 0 | 60 | 60 |
| **RZ** | 9 | 51 | 60 | **RZK** | 15 | 46 | 61 | **RZK** | 15 | 46 | 61 |
| **total** | 9 | 111 | 120 | **total** | 24 | 97 | 121 | **total** | 15 | 106 | 121 |
| **p-value** |  |  | **0.001413763** | **p-value** |  |  | **0.0768902** | **p-value** |  |  | **1.3063E-05** |
| **Significance** |  |  | ******** | **Significance** |  |  | **ns** | **Significance** |  |  | ******** |
|  | **Class 4** | **rest** | **total** |  | **Class 4** | **rest** | **total** |  | **Class 4** | **rest** | **total** |
| **WT** | 0 | 60 | 60 | **RZ** | 0 | 60 | 60 | **WT** | 0 | 60 | 60 |
| **RZ** | 0 | 60 | 60 | **RZK** | 19 | 42 | 61 | **RZK** | 19 | 42 | 61 |
| **total** | 0 | 120 | 120 | **total** | 19 | 102 | 121 | **total** | 19 | 102 | 121 |
| **p-value** |  |  | **1** | **p-value** |  |  | **4.2911E-07** | **p-value** |  |  | **4.2911E-07** |
| **Significance** |  |  | **ns** | **Significance** |  |  | ******** | **Significance** |  |  | ******** |

| **Axin2** |  |  |  |  |  |  |  |  |  |  |  |
| --- | --- | --- | --- | --- | --- | --- | --- | --- | --- | --- | --- |
|  | **Class 0** | **rest** | **total** |  | **Class 0** | **rest** | **total** |  | **Class 0** | **rest** | **total** |
| **WT** | 40 | 20 | 60 | **RZ** | 2 | 58 | 60 | **WT** | 40 | 20 | 60 |
| **RZ** | 2 | 58 | 60 | **RZK** | 1 | 61 | 62 | **RZK** | 1 | 61 | 62 |
| **total** | 42 | 78 | 120 | **total** | 3 | 119 | 122 | **total** | 41 | 81 | 122 |
| **p-value** |  |  | **1.76471E-14** | **p-value** |  |  | **0.3716976** | **p-value** |  |  | **5.1039E-16** |
| **Significance** |  |  | ******** | **Significance** |  |  | **ns** | **Significance** |  |  | ******** |
|  | **Class 1** | **rest** | **total** |  | **Class 1** | **rest** | **total** |  | **Class 1** | **rest** | **total** |
| **WT** | 14 | 46 | 60 | **RZ** | 17 | 43 | 60 | **WT** | 14 | 46 | 60 |
| **RZ** | 17 | 43 | 60 | **RZK** | 5 | 57 | 62 | **RZK** | 5 | 57 | 62 |
| **total** | 31 | 89 | 120 | **total** | 22 | 100 | 122 | **total** | 19 | 103 | 122 |
| **p-value** |  |  | **0.136294293** | **p-value** |  |  | **0.00266172** | **p-value** |  |  | **0.01369261** |
| **Significance** |  |  | **ns** | **Significance** |  |  | ******** | **Significance** |  |  | ****** |
|  | **Class 2** | **rest** | **total** |  | **Class 2** | **rest** | **total** |  | **Class 2** | **rest** | **total** |
| **WT** | 4 | 56 | 60 | **RZ** | 21 | 39 | 60 | **WT** | 4 | 56 | 60 |
| **RZ** | 21 | 39 | 60 | **RZK** | 17 | 45 | 62 | **RZK** | 17 | 45 | 62 |
| **total** | 25 | 95 | 120 | **total** | 38 | 84 | 122 | **total** | 21 | 101 | 122 |
| **p-value** |  |  | **9.32593E-05** | **p-value** |  |  | **0.10366383** | **p-value** |  |  | **0.0017589** |
| **Significance** |  |  | ******** | **Significance** |  |  | **ns** | **Significance** |  |  | ******** |
|  | **Class 3** | **rest** | **total** |  | **Class 3** | **rest** | **total** |  | **Class 3** | **rest** | **total** |
| **WT** | 1 | 59 | 60 | **RZ** | 13 | 47 | 60 | **WT** | 1 | 59 | 60 |
| **RZ** | 13 | 47 | 60 | **RZK** | 22 | 40 | 62 | **RZK** | 22 | 40 | 62 |
| **total** | 14 | 106 | 120 | **total** | 35 | 87 | 122 | **total** | 23 | 99 | 122 |
| **p-value** |  |  | **0.000463109** | **p-value** |  |  | **0.03910442** | **p-value** |  |  | **5.0303E-07** |
| **Significance** |  |  | ******** | **Significance** |  |  | ***** | **Significance** |  |  | ******** |
|  | **Class 4** | **rest** | **total** |  | **Class 4** | **rest** | **total** |  | **Class 4** | **rest** | **total** |
| **WT** | 1 | 59 | 60 | **RZ** | 7 | 53 | 60 | **WT** | 1 | 59 | 60 |
| **RZ** | 7 | 53 | 60 | **RZK** | 17 | 45 | 62 | **RZK** | 17 | 45 | 61 |
| **total** | 8 | 112 | 120 | **total** | 24 | 98 | 122 | **total** | 18 | 104 | 121 |
| **p-value** |  |  | **0.02757761** | **p-value** |  |  | **0.01691886** | **p-value** |  |  | **2.523E-05** |
| **Significance** |  |  | ***** | **Significance** |  |  | ****** | **Significance** |  |  | ******** |

**Supplementary Table3.** Statistical analysis of Quantification of ISH (Figure 2h)
