# Supplementary material for: Epithelial WNT secretion drives niche escape of developing gastric cancer
Source: Mol Cancer. 2025 Dec 16;25:1. doi: 10.1186/s12943-025-02543-z (PMC12766950; doi:10.1186/s12943-025-02543-z)
Supplement: Supplementary file 2 — Supplementary Material 2: Supplementary Figure 1 - 9. [file 12943_2025_2543_MOESM2_ESM.docx]

. **
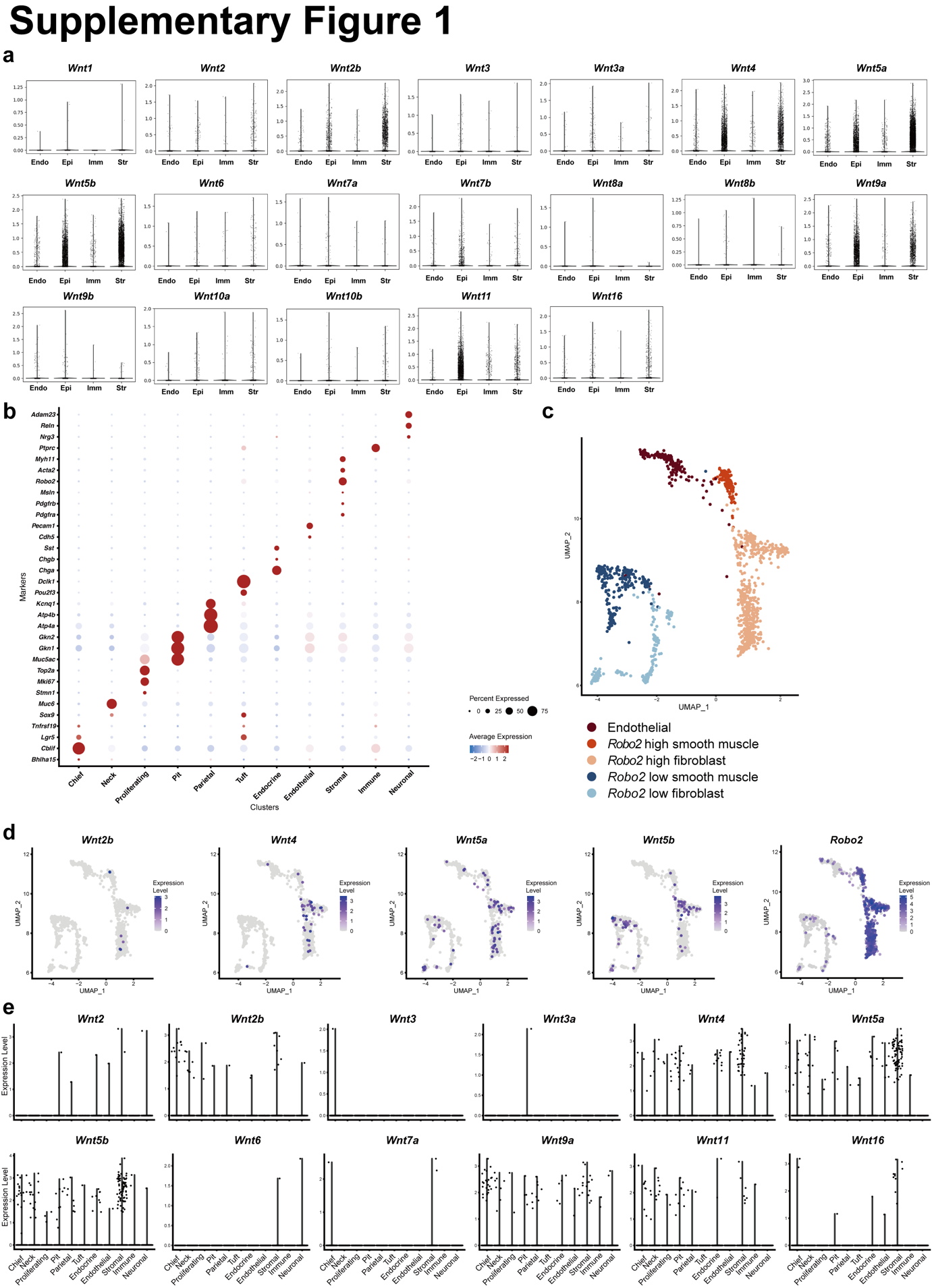
**

**Supplementary Figure 1. Expression patterns of *Wnt* genes in WT mouse gastric tissue**

(a) Expression patterns of all *Wnt* genes from the data of Zhang *et al.* (2024)^32^. Endo: Endothelial cell lineage; Epi: Epithelial cell lineage; Imm: lmmune cell lineage; Str: Stromal cell lineage.

(b) Dot plot showing marker gene expression across cell types in WT mouse gastric corpus tissue sn multiome data. Average expression levels are indicated by color, and the number of expressing cells by dot size.

(c) Stromal cell populations were extracted from UMAP in Fig. 1b and re-annotated for more detailed characterization.

(d) Feature plots of *Wnt* genes and *Robo2* expression within the highlighted stromal region of the WT mouse gastric tissue sn multiome UMAP.

(e) Expression patterns of all detectable *Wnt* genes from the WT mouse gastric corpus sn multiome data.

**
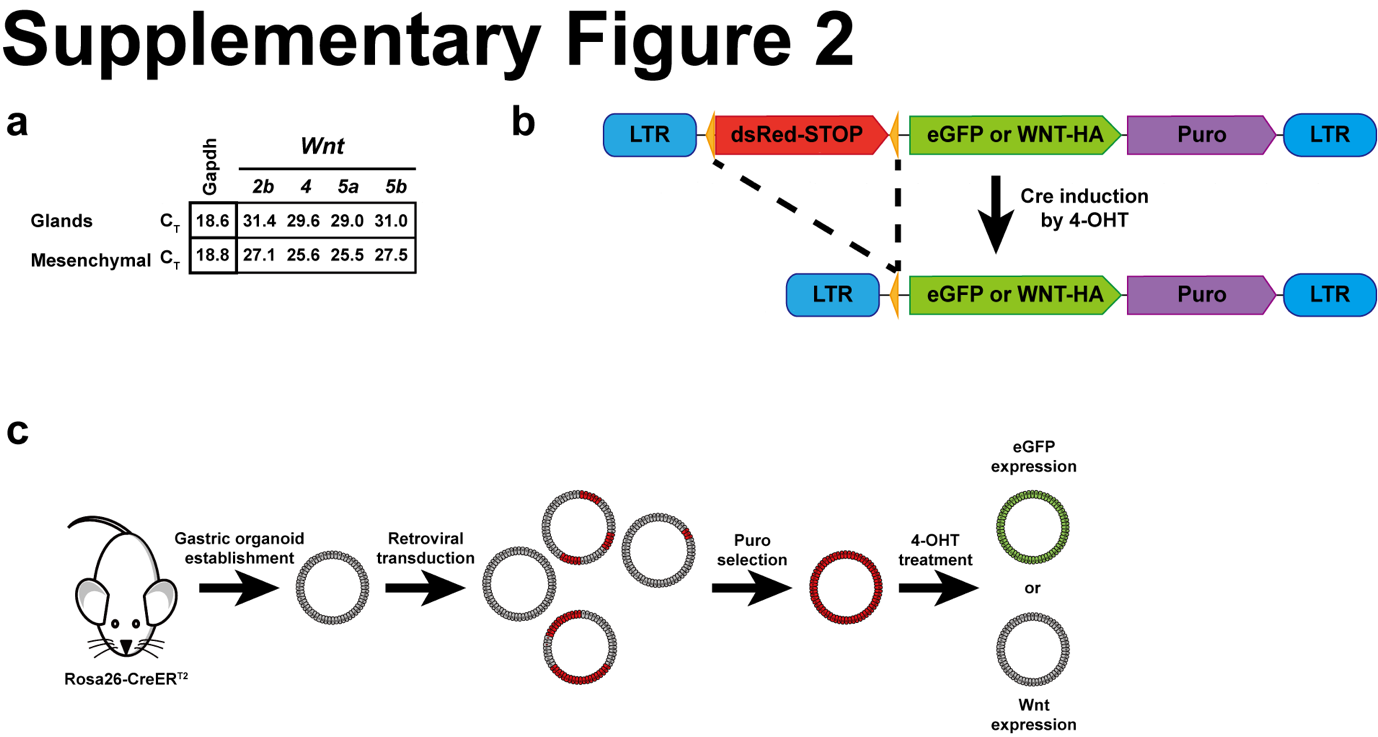
**

**Supplementary Figure 2. Retrovirus-mediated gene overexpression in gastric corpus organoids**

(a) Calculated C_T_ values for expression of a panel of *Wnt* genes in epithelial and mesenchyme-enriched populations.

(b) Schematic of Cre-mediated recombination for the *eGFP* or *Wnt* expression.

(c) Experimental workflow for retrovirus-mediated *eGFP* or *Wnt* expression in gastric corpus organoids.

**
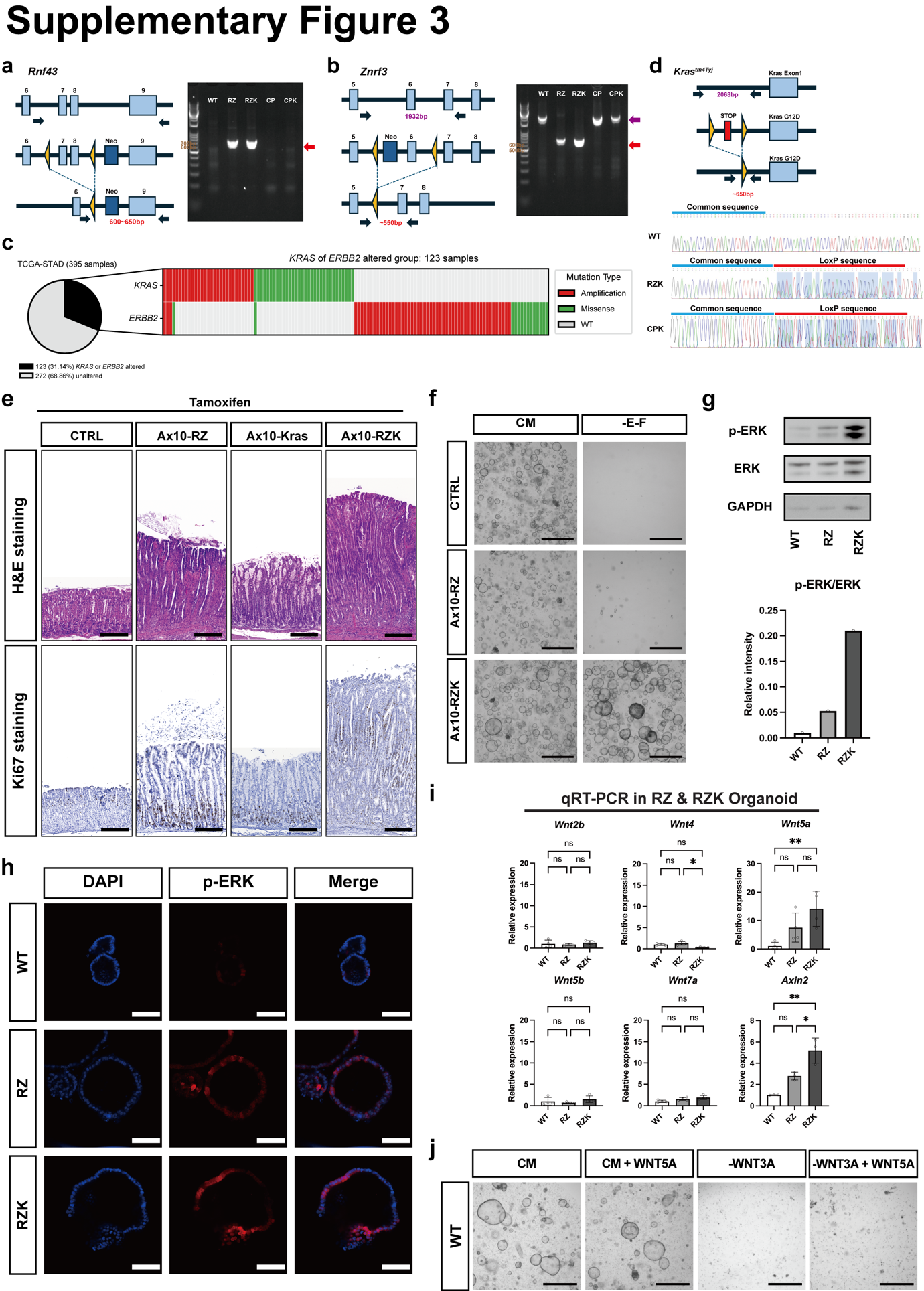
**

**Supplementary Figure 3.** **Generation and genotyping of Ax10-RZ and Ax10-RZK mouse lines**

(a) Schematic and primer binding sites for *Rnf43^f/f^* allele generation. Right: genotyping gel.

(b) Schematic and primer binding sites for *Znrf3^f/f^* allele generation. Right: genotyping gel.

(c) Mutation patterns of KRAS or ERBB2 at the DNA level were examined in the TCGA-STAD (stomach adenocarcinoma) dataset. Samples with available DNA alteration data were filtered, resulting in a total of 395 samples included in the analysis. Red: samples with gene amplification, Green: samples with missense mutations, Gray: samples with non-mutated.

(d) Schematic and primer binding sites for *lsl-Kras^G12D^* (*Kras^Tm4Tyj^*) allele generation. Below: sequencing results to identifying loxP site.

(e) H&E and Ki67 immunohistochemistry of corpus epithelium from control (CTRL), *Anxa10-Cre^ERT2^; Rnf43^f/f^; Znrf3^f/f^* (Ax10-RZ), *Anxa10-Cre^ERT2^; lsl-Kras^G12D^* (Ax10-Kras), and *Anxa10-Cre^ERT2^; Rnf43^f/f^; Znrf3^f/f^; lsl-Kras^G12D^* (Ax10-RZK) mice, 1 month after tamoxifen induction. CTRL, Ax10-RZ, and Ax10-RZK data are the same as in Fig. 2a. Representative images from 2–4 mice per genotype are shown. Scale bars: 100 μm.

(f) Niche requirements for CTRL, Ax10-RZ, and Ax10-RZK gastric organoids. Organoid growth was examined after 4 passages. Healthy organoids are cystic with a clear center. Representative images of organoids isolated from 2–4 mice per genotype are shown. CM: complete medium (WENRFG); -E-F: WNRG medium. Scale bars: 1000 μm.

(g) Protein levels of ERK and p-ERK in WT, RZ, RZK gastric organoid samples were assessed by western blotting. WT organoids were cultured in complete medium, RZ organoids in -W medium and RZK organoids in -W-R medium. Relative band intensities were normalized to GAPDH, and the p-ERK/ERK ratio was subsequently calculated.

(h) Representative immunofluorescence images of WT, RZ, RZK organoids. Blue: DAPI; Red: p-ERK. Scale bars: 100 μm. RZK organoid images are also shonw in Supplementary Fig. 6e. All samples were processed in the same experimental batch.

(i) Bar graphs indicating the expression of *Wnt* genes, as determined by qRT-PCR of RNA isolated from CTRL, Ax10-RZ, and Ax10-RZK organoids cultured in CM for 5 days. Expression is normalized to GAPDH. n = 4 biological replicates. Error bars represent SD. Statistical significance was determined by ANOVA. **, p<0.005; *, p<0.05; ns, non-significant.

(j) Representative images of WT gastric organoids after 1 passage under continuous WNT5A treatment. Healthy organoids are cystic with a clear center. CM: complete medium (WENRFG); -WNT3A: ENRFG medium. Scale bars: 1000 μm.


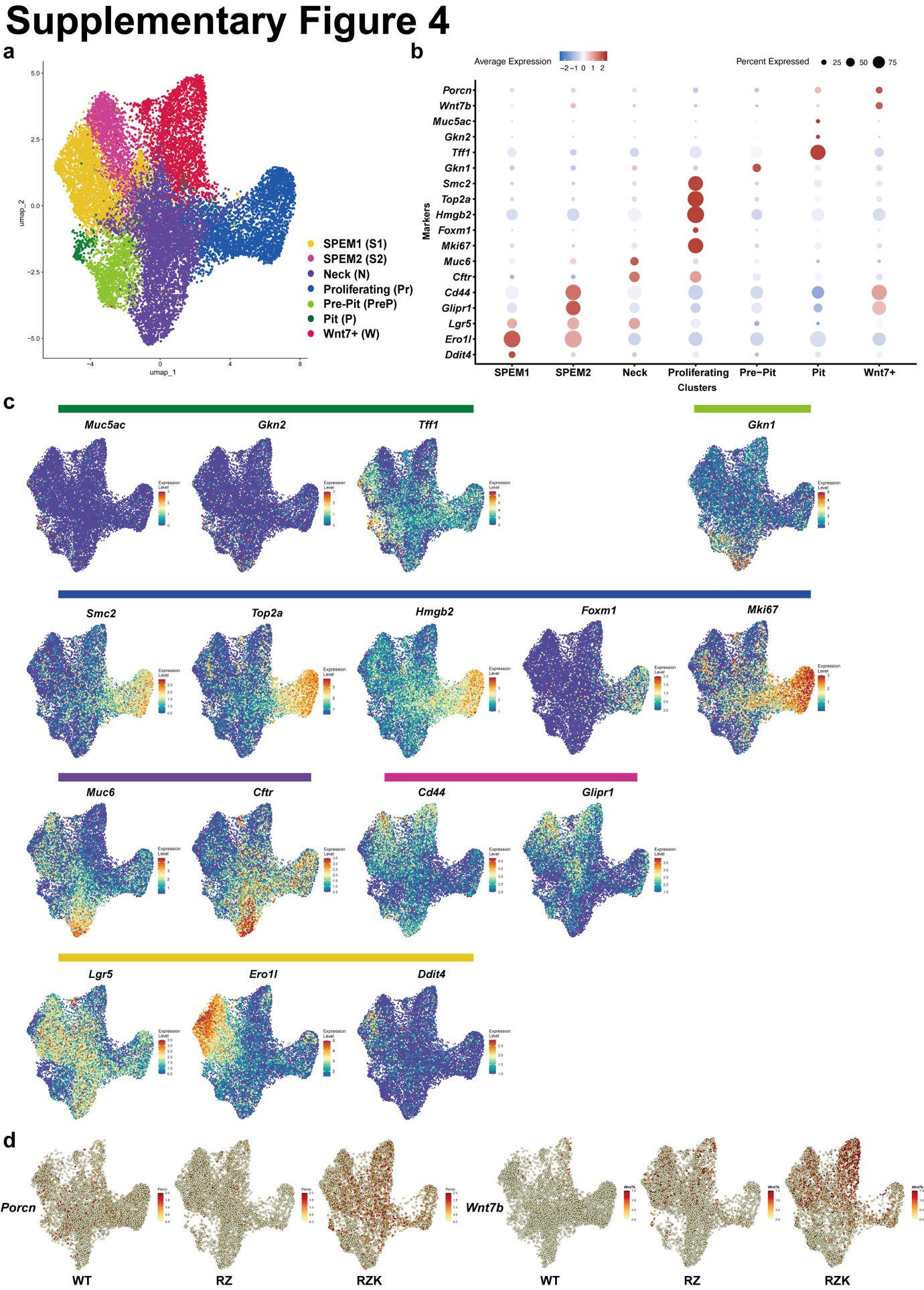


**Supplementary Figure 4. Cell type annotation and gene expression in WT, RZ, and RZK organoid sn multiome data.**

(a) UMAP showing integrated clustering of all nuclei from WT, RZ, and RZK organoids, including 6,111 WT nuclei, 5,468 RZ nuclei, and 5,341 RZK nuclei.

(b) Dot plot showing the expression of marker genes used for clustering in the WT, RZ, and RZK organoid sn multiome dataset.

(c) Feature plots of the marker genes used for UMAP clustering.

(d) Feature plots showing the expression of *Porcn* and *Wnt7b* in WT, RZ, RZK organoids.


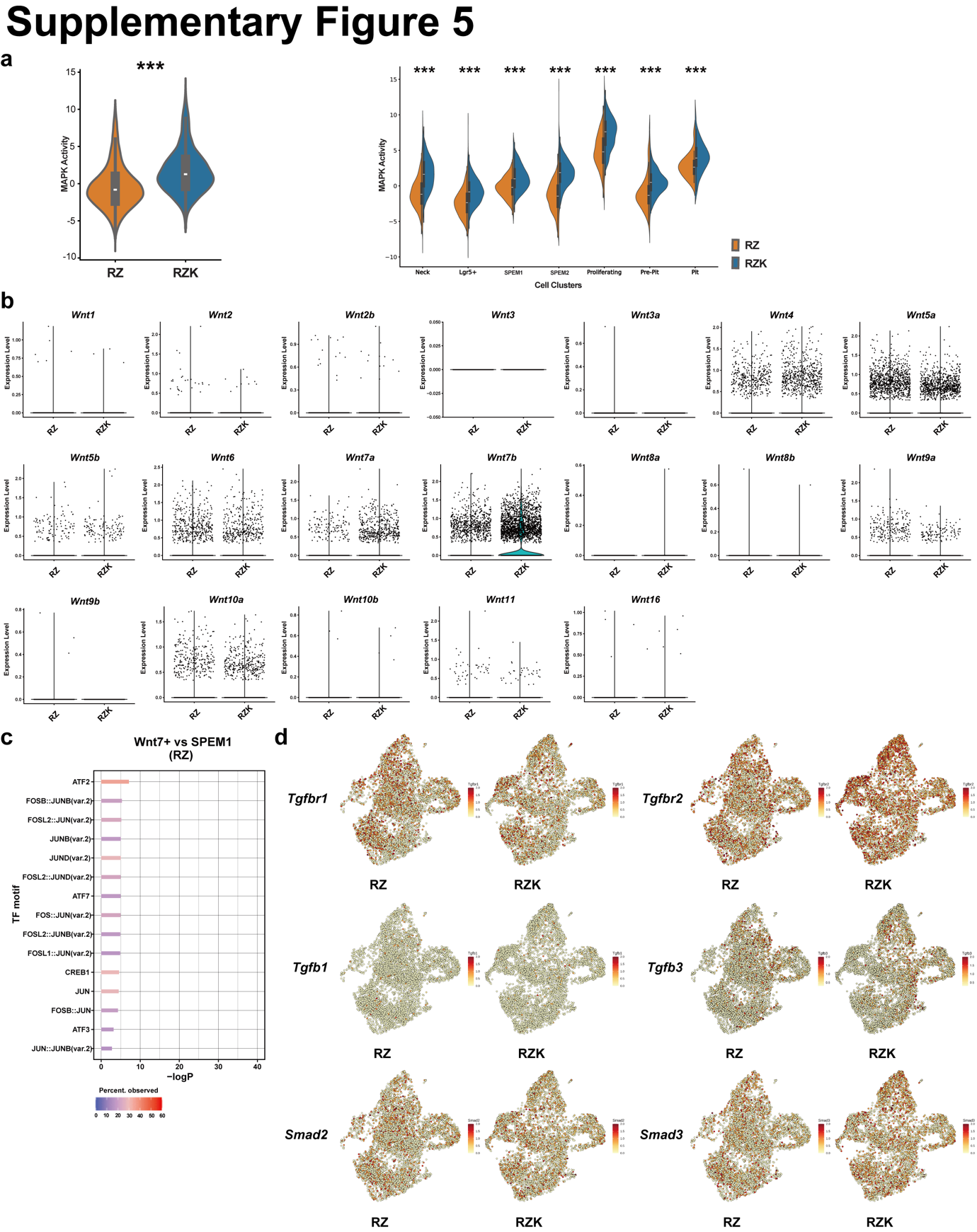


**Supplementary Figure 5. KRAS activation primes the SPEM population for differentiation to WNT7B producing cells**

(a) MAPK activity was analyzed in RZ and RZK organoids at both the whole-population level and across individual cell types. Statistical significance was determined using the Wilcoxon test. ***, p<0.001.

(b) Violin plots showing *Wnt* gene expression in Ax10-RZ and Ax10-RZK organoids.

(c) Motif enrichment analysis from differential accessible peak analysis using sn multiome data from Wnt7+, compared to the SPEM1 cluster in the Ax10-RZ. Enrichment is depicted as a percentage on a -logP scale.

(d) Feature plots showing the expression of *Tgfbr1, Tgfbr2, Tgfb1, Tgfb3, Smad2,* and *Smad3* in RZ, RZK organoids.

**
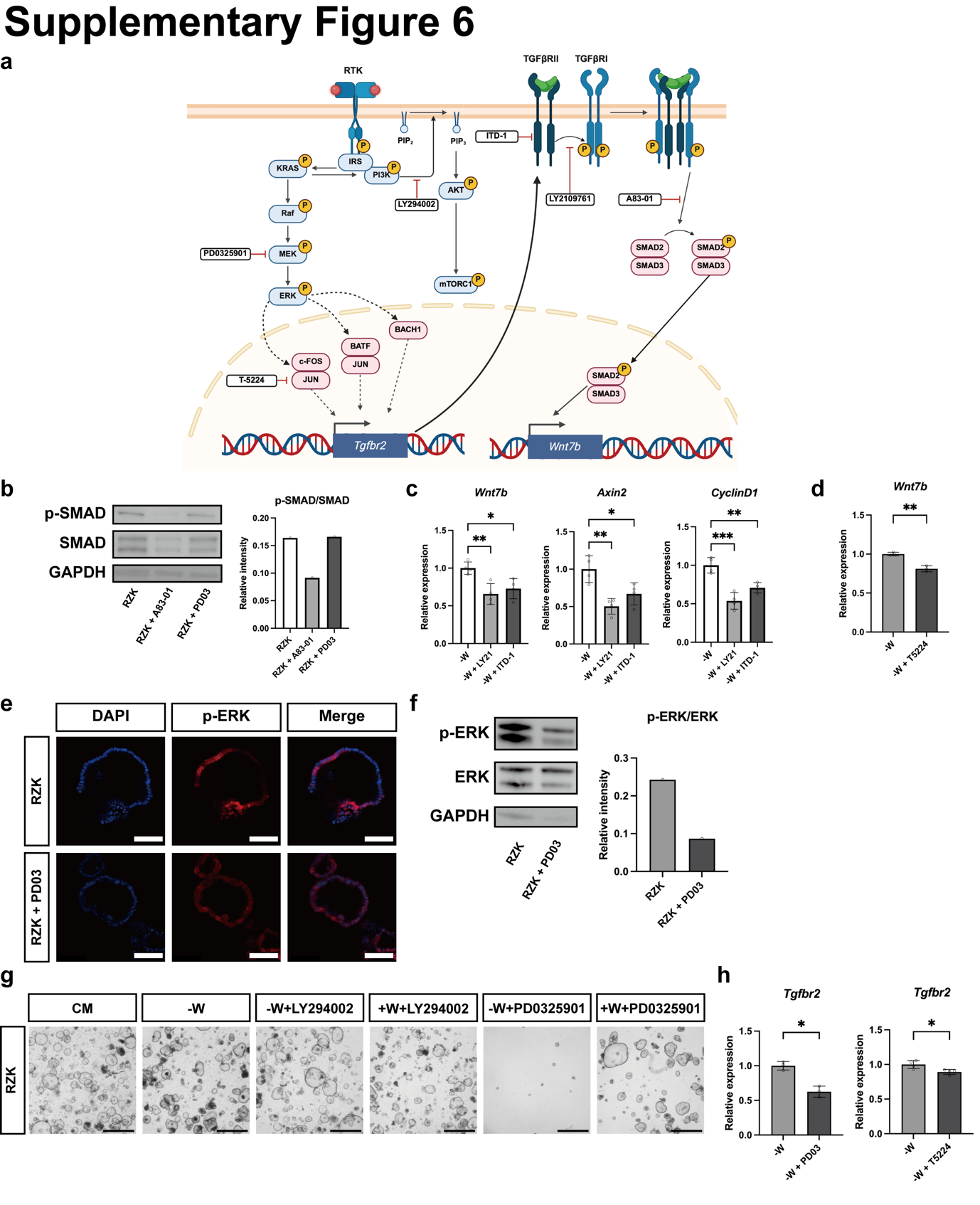
**

**Supplementary Figure 6. Mechanistic evidence for SMAD2/3-mediated control of *Wnt7b* expression.**

(a) Scheme for the RTK signaling pathway and its inhibitors^109^.

(b) Protein levels of SMAD and p-SMAD in RZK gastric organoid samples were assessed by western blotting. RZK organoids were cultured in -W medium, -W medium with A83-01 (2μM) and -W medium with PD0325901 (1μM). Relative band intensities were normalized to GAPDH, and the p-SMAD/SMAD ratio was subsequently calculated.

(c) Bar graphs indicating the expression of *Wnt7b* and WNT downstream genes, as determined by qRT-PCR of RNA isolated from RZK organoids under -W medium, -W medium with LY2109761 (5μM), -W medium with ITD-1 (5μM) conditions. Expression is normalized to GAPDH. Error bars represent SD. Statistical significance was determined by ANOVA. *, p<0.05.

(d) Bar graphs indicating the expression of *Wnt7b* gene, as determined by qRT-PCR of RNA isolated from RZK organoids under -W, -W medium with T-5224 (50μM) conditions. Expression is normalized to GAPDH. n = 4 biological replicates. Error bars represent SD. Statistical significance was determined by paired t-test. **, p<0.005.

(e) Representative immunofluorescence images of RZK organoid cultured in -W condition and -W+PD0325901(1μM). Blue: DAPI; Red: p-ERK. Scale bars: 100 μm. RZK organoid images are also shonw in Supplementary Fig. 3h. All samples were processed in the same experimental batch.

(f) Protein levels of ERK and p-ERK in RZK gastric organoid samples were assessed by western blotting. RZK organoids were cultured in -W medium and -W medium with PD0325901 (1μM). Relative band intensities were normalized to GAPDH, and the p-ERK/ERK ratio was subsequently calculated.

(g) Treatment of Ax10-RZK gastric organoids with PI3K inhibitor (LY294002, 10μM) and MEK inhibitor (PD0325901, 5μM). Organoid growth was examined after 3 passages with drug treatment. Healthy organoids are cystic with a clear center. Representative images of organoids are shown. Scale bars: 1000 μm.

(h) Bar graphs indicating the expression of *Tgfbr2* gene, as determined by qRT-PCR of RNA isolated from RZK organoids under -W, -W medium with PD0325901 (1μM) conditions. Expression is normalized to GAPDH. n = 3-4 biological replicates. Error bars represent SD. Statistical significance was determined by paired t-test. *, p<0.05.

**
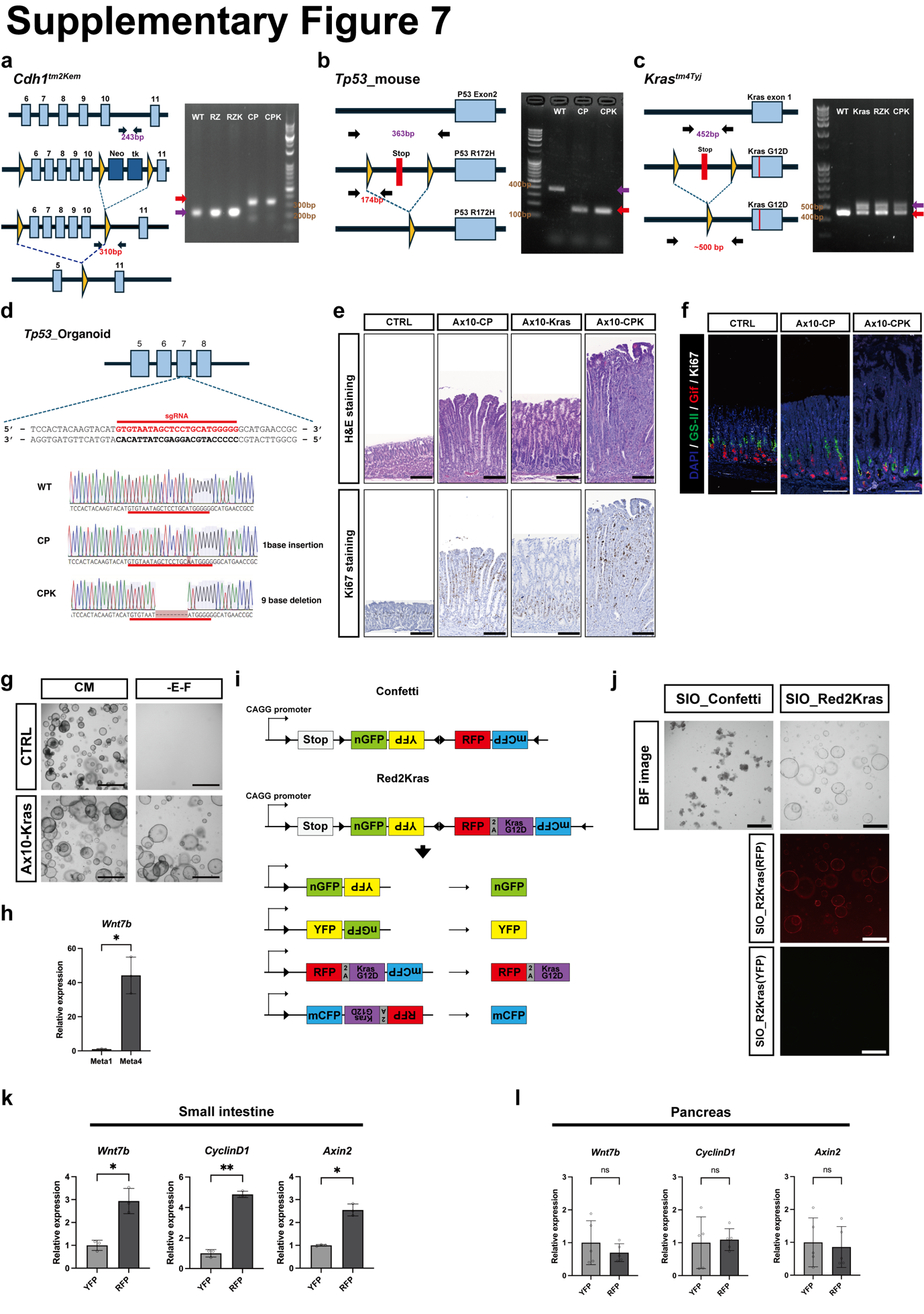
**

**Supplementary Figure 7. Generation of Ax10-CP, Ax10-CPK, and Ax10-Kras mouse lines and the effects of Kras activation in different contexts.**

(a) Schematic and primer binding sites for *Cdh1^f/f^* (*Cdh1^tm2Kem^*) allele generation. Right: genotyping gel.

(b) Schematic and primer binding sites for *Tp53^f/f^* (*Tp53^Tm2Tyj^*) allele generation. Right: genotyping gel.

(c) Schematic and primer binding sites for *lsl-Kras^G12D^* (*Kras^Tm4Tyj^*) allele generation. Right: genotyping gel.

(d) Schematic and sgRNA design of *Kras^f/f^* allele generation. Below: sequencing results.

(e) H&E and Ki67 immunohistochemistry of corpus epithelium from control (CTRL), *Anxa10-Cre^ERT2^; Cdh1^f/f^; Tp53^f/f^* (Ax10-CP), *Anxa10-Cre^ERT2^; lsl-Kras^G12D^* (Ax10-Kras), and *Anxa10-Cre^ERT2^; Cdh1^f/f^; Tp53^f/f^; lsl-Kras^G12D^* (Ax10-CPK) mice, 1 month after tamoxifen induction. Ax10-CP and Ax10-CPK data are the same as in Fig. 5a. Representative images from 2–4 mice per genotype are shown. Scale bars: 100 μm.

(f) Immunofluorescence of corpus epithelium from control (CTRL), *Anxa10-Cre^ERT2^; Cdh1^f/f^; Tp53^f/f^* (Ax10-CP), and *Anxa10-Cre^ERT2^; Cdh1^f/f^; Tp53^f/f^; lsl-Kras^G12D^* (Ax10-CPK) mice, 1 month after tamoxifen induction. Representative images of 2–4 mice per genotype are shown. Blue: DAPI; Green: GS-Ⅱ (Neck cell marker); Red: Gif (Chief cell marker); White: Ki67. Scale bars: 100 μm.

(g) Niche requirements for CTRL and Ax10-Kras gastric organoids. Organoid growth was examined after 2 passages. Healthy organoids are cystic with a clear center. Representative images of organoids are shown. CM: complete medium (WENRFG); -E-F: WNRG. Scale bars: 1000 μm.

(h) Bar graph indicating the expression of *Wnt7b*, as determined by qRT-PCR of RNA isolated from Meta1 and Meta4 organoids. Expression is normalized to Actin. n = 3 biological replicates. Error bars represent SD. Statistical significance was determined by paired t-test. *, p<0.05.

(i) Schematic of Confetti and Red2Kras alleles.

(j) Comparison of YFP and RFP clones from small intestine organoids of mice with the Confetti allele and the Red2Kras allele, respectively. All organoids were cultured in ENR media. Scale bars: 1000 μm.

(k) Bar graphs indicating the expression of *Wnt7b* and WNT downstream genes, as determined by qRT-PCR of RNA isolated separately from YFP and RFP clones of Red2Kras small intestine organoids. RNA for WNT ligands was obtained by culturing in WENR media for 5 days, while RNA for WNT downstream targets was collected after culturing in ENR media for 5 days. Expression is normalized to GAPDH. n = 3 biological replicates. Error bars represent SD. Statistical significance was determined by paired t-test. **, p<0.005; *, p<0.05.

(l) Bar graphs indicating the expression of *Wnt7b* and WNT downstream genes, as determined by qRT-PCR of RNA isolated separately from YFP and RFP clones of Red2Kras pancreas ductal organoids. Expression is normalized to GAPDH. n = 5 biological replicates. Error bars represent SD. Statistical significance was determined by paired t-test. ns, non-significant


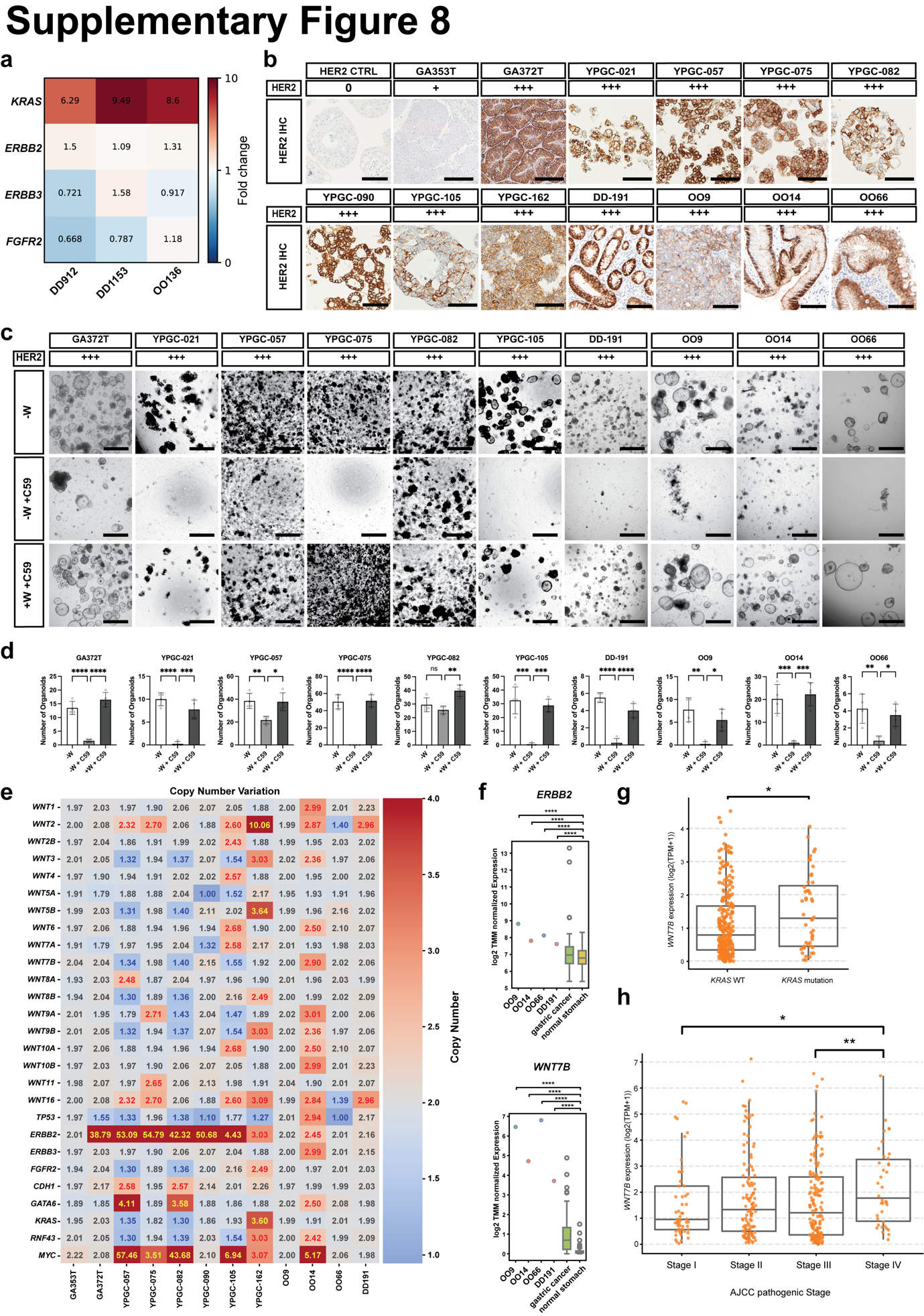


**Supplementary Figure 8. Validation of WNT independence in gastric cancer patient-derived organoids**

(a) Fold change analysis of KRAS-amplified samples based on panel sequencing (Fig. 6a). Scale bar indicates absolute fold change values.

(b) HER2 immunohistochemistry of corpus organoids derived from a human gastric cancer patient. HER2 levels are classified as negative (0, +) and positive (+++) based on guidelines published by Bartley *et al*.^58^. Scale bars: 100 μm.

(c) Niche requirements of human gastric cancer patient-derived organoids (GC-hPDOs) except those shown in Fig. 6c, 6h(YPGC-090, YPGC162). Organoid growth was examined after 2-3 passages. Healthy organoid growth is cystic with a clear center or grow in grape-like structures. -W: ENRFG; -W+C59: ENRFG with C59 (10 μM); +W +C59: WENRFG with C59 (10 μM). Scale bars: 1000 μm.

(d) Organoids in Supplementary Fig. 6c were quantified by counting those with a diameter of 150 µm or larger. Error bars represent SD. Statistical significances was determined by ANOVA. ****, p<0.00005; ***, p<0.0005; **, p<0.005; *, p<0.05 ns, non-significant.

(e) Copy number variation analysis of GC-hPDOs. Scale bar indicates absolute value of copy number.

(f) Bulk RNA sequencing results of GC-hPDOs, including the OO lines, were compared with gastric cancer and normal gastric tissues using public data. Statistical analysis was conducted by Wilcoxon test. ****, p<0.00005.

(g) *WNT7B* expression patterns were examined according to *KRAS* mutation status in TCGA-STAD (stomach adenocarcinoma) data set. During the analysis, samples with *ERBB2, ERBB3*, or *FGFR2* mutations were excluded to specifically assess *KRAS*-driven effect. Statistical significance was determined using the Mann-Whitney U test. *, p<0.05

(h) *WNT7B* expression was examined across tumors categorized by AJCC classification in the TCGA-STAD dataset to reflect cancer progression and malignancy. Statistical significance was determined using the Mann-Whitney U test. *, p<0.05


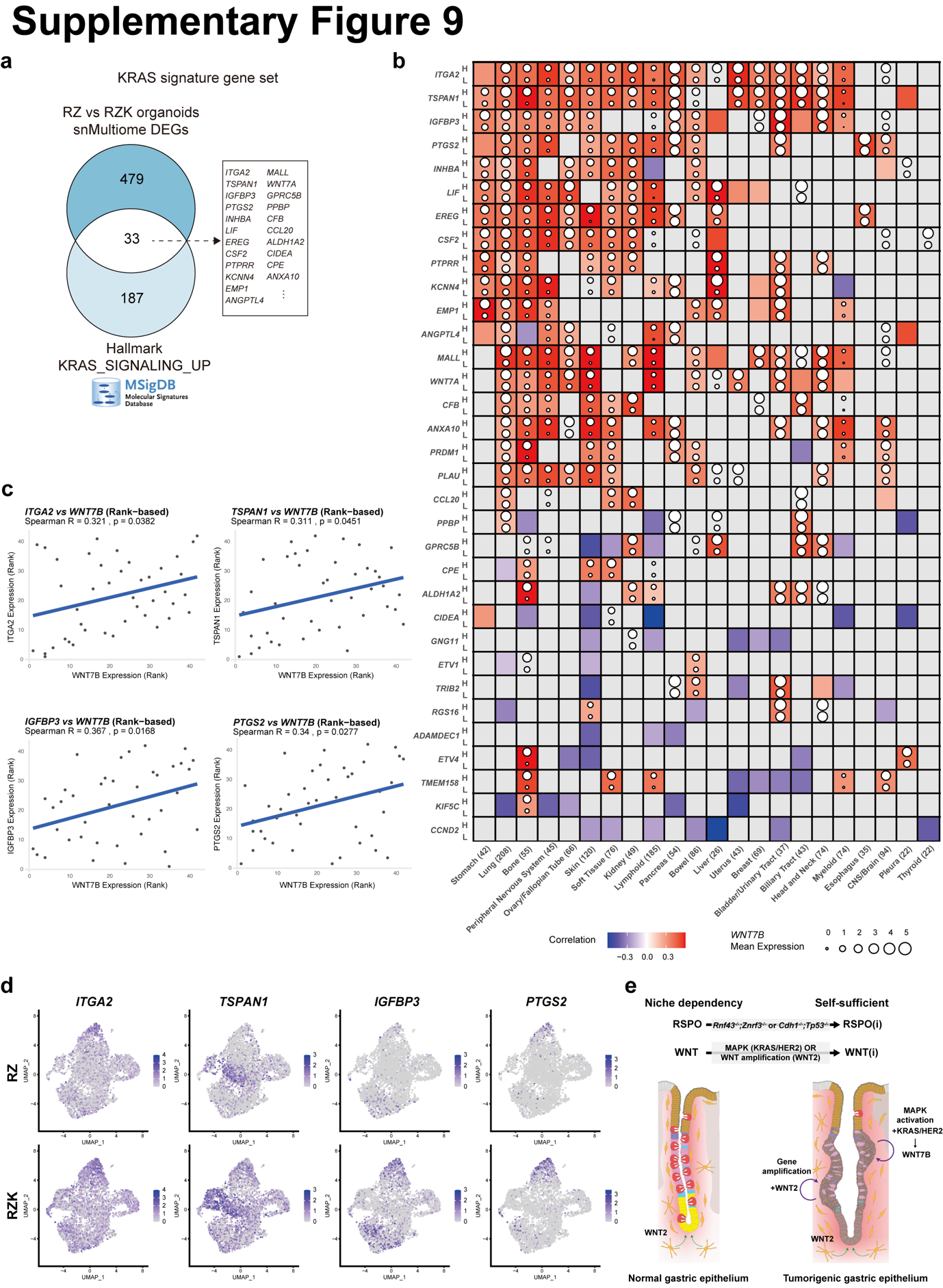


**Supplementary Figure 9. Cross-organ analysis of the KRAS-WNT7B axis in cancer cell lines**

(a) Venn diagram illustrating the scheme for selecting the KRAS signature gene set.

(b) Spearman correlation between 33 KRAS signature genes and *WNT7B* expression in cancer cell lines from different organs (color) and comparison of *WNT7B* expression (circle). Gray color indicates p> 0.05. H: high quartile group (Q1), L: low quartile group (Q4).

(c) Representative genes (*ITGA2, TSPAN1, IGFBP3, PTGS2*) correlated with *WNT7B* expression in the stomach.

(d) Expression patterns of KRAS-WNT7B axis candidate marker genes in RZ and RZK organoids from sn multiome data in Fig. 3a.

(e) In normal gastric epithelium, WNT ligands such as WNT2B are supplied by the mesenchyme. In tumorigenic gastric epithelium, however, WNT ligand independence is achieved via various pathways.
