# Supplementary material for: Epithelial WNT secretion drives niche escape of developing gastric cancer
Source: Mol Cancer. 2025 Dec 16;25:1. doi: 10.1186/s12943-025-02543-z (PMC12766950; doi:10.1186/s12943-025-02543-z)
Supplement: Supplementary file 3 — Supplementary Material 3: Raw images of gels and membranes used in Supplementary figures. [file 12943_2025_2543_MOESM3_ESM.pptx]

## Slide 1
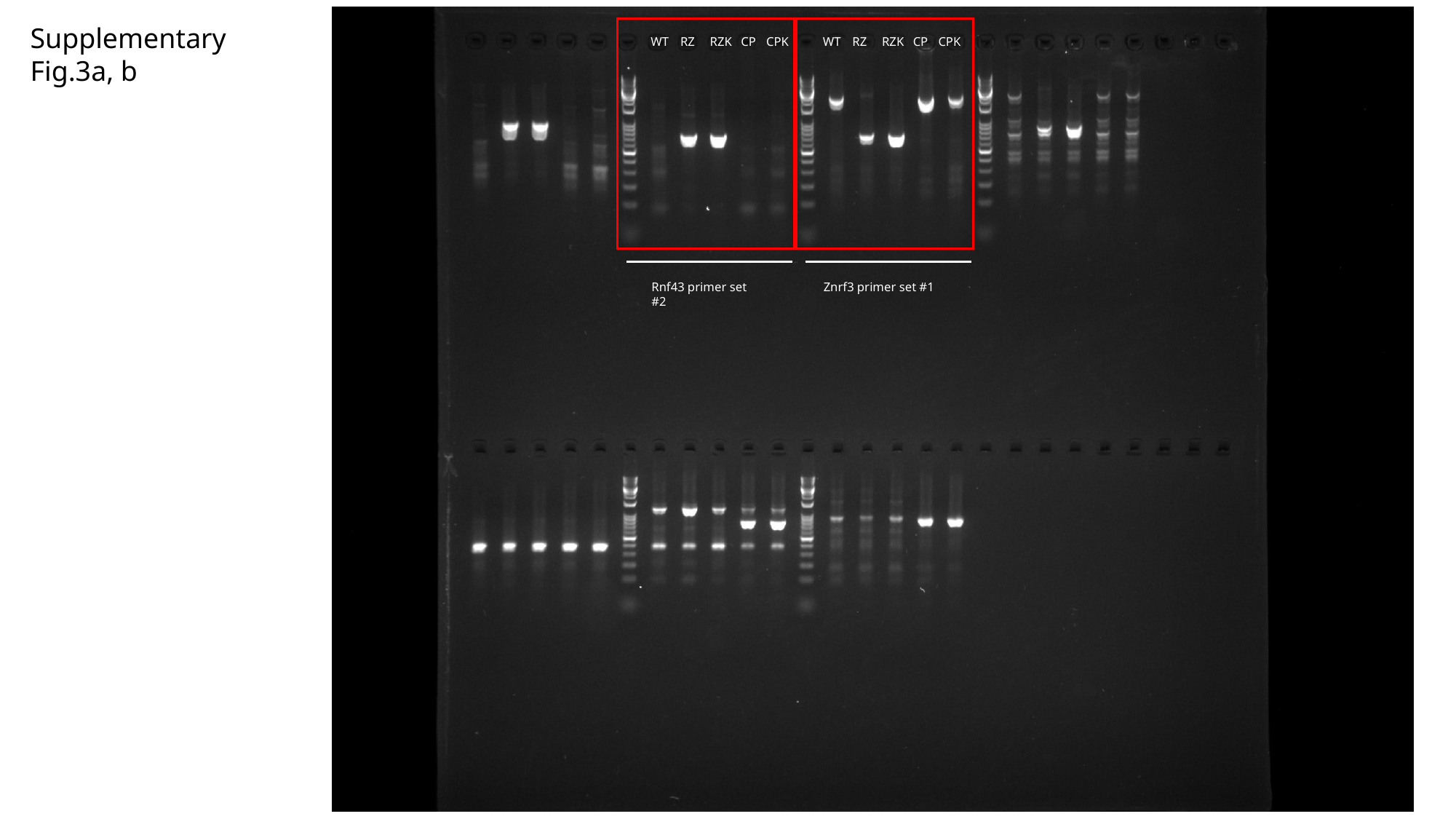

Supplementary Fig.3a, b
WT
RZ
RZK
CP
CPK
WT
RZ
RZK
CP
CPK
Rnf43 primer set #2
Znrf3 primer set #1

## Slide 2
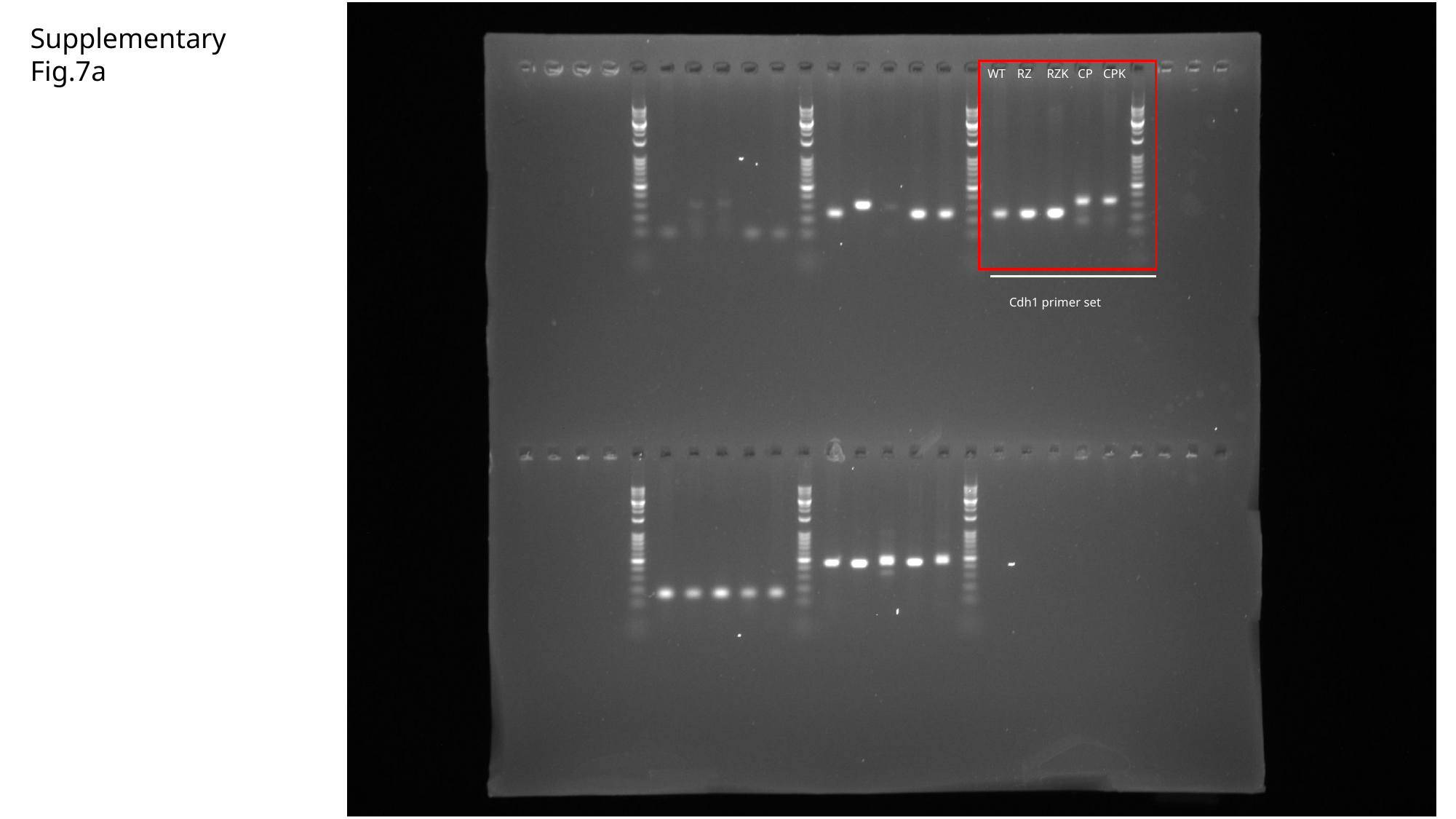

Supplementary Fig.7a
WT
RZ
RZK
CP
CPK
Cdh1 primer set

## Slide 3
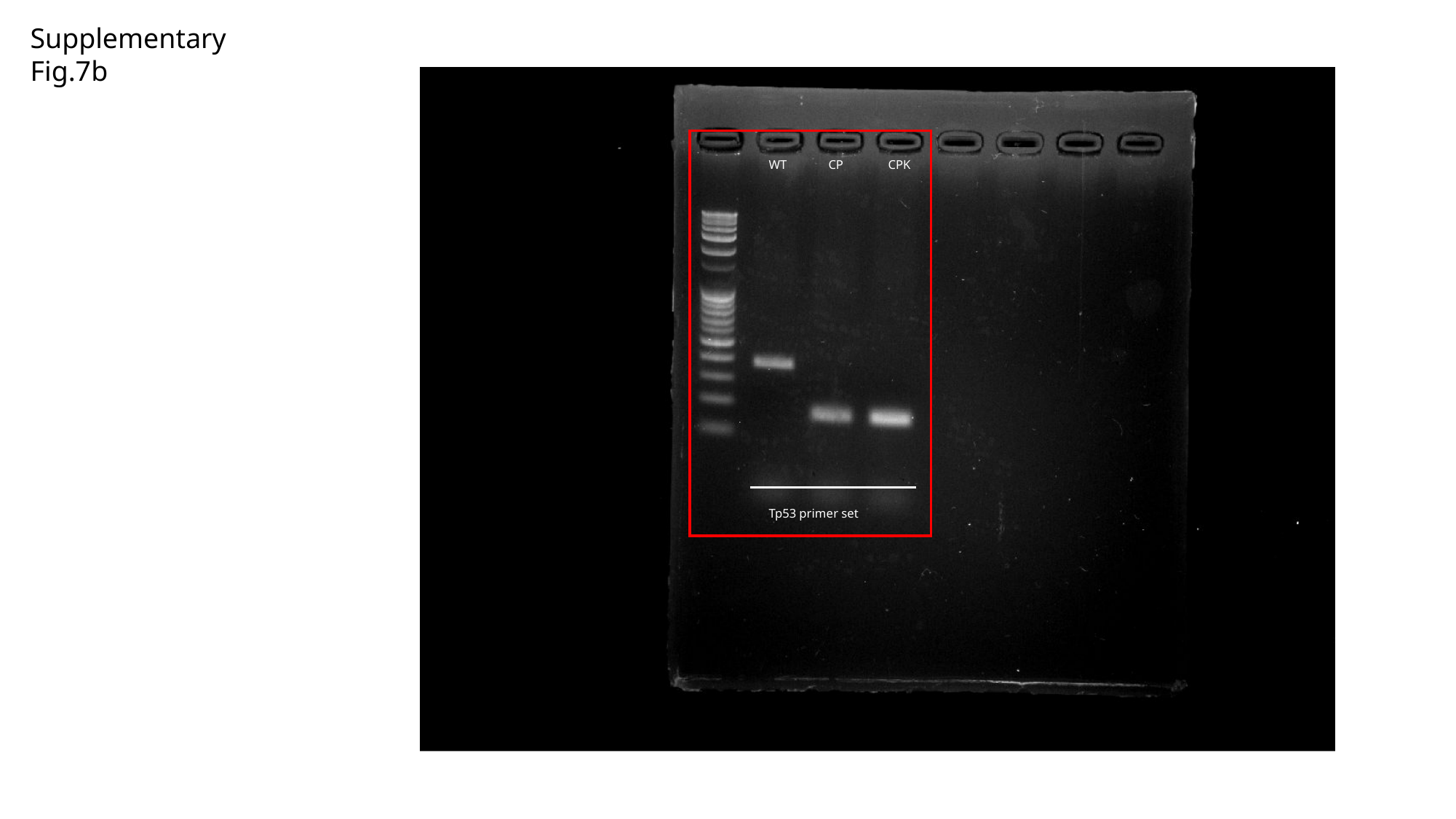

Supplementary Fig.7b
WT
CP
CPK
Tp53 primer set

## Slide 4
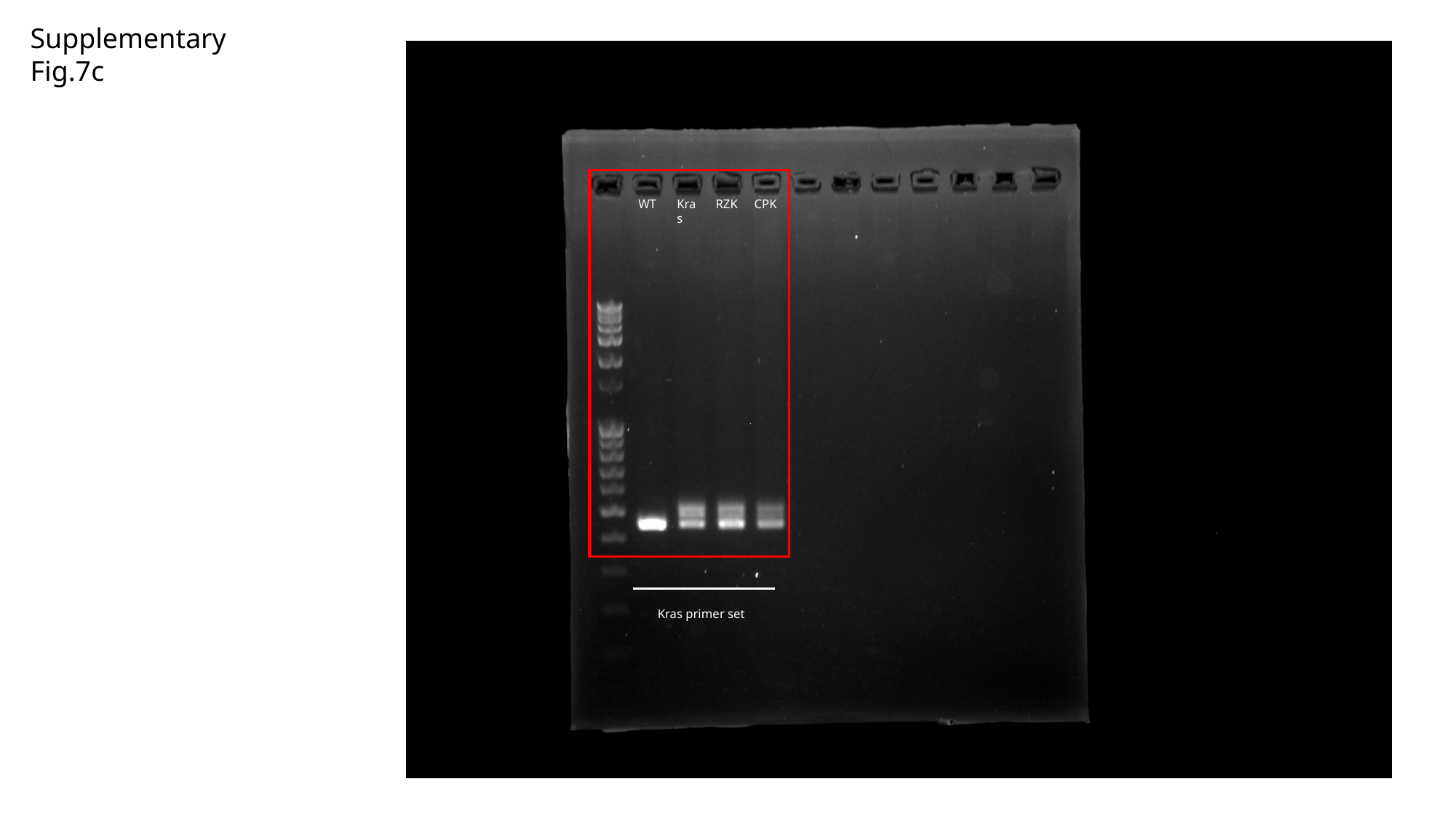

Supplementary Fig.7c
WT
RZK
CPK
Kras
Kras primer set

## Slide 5
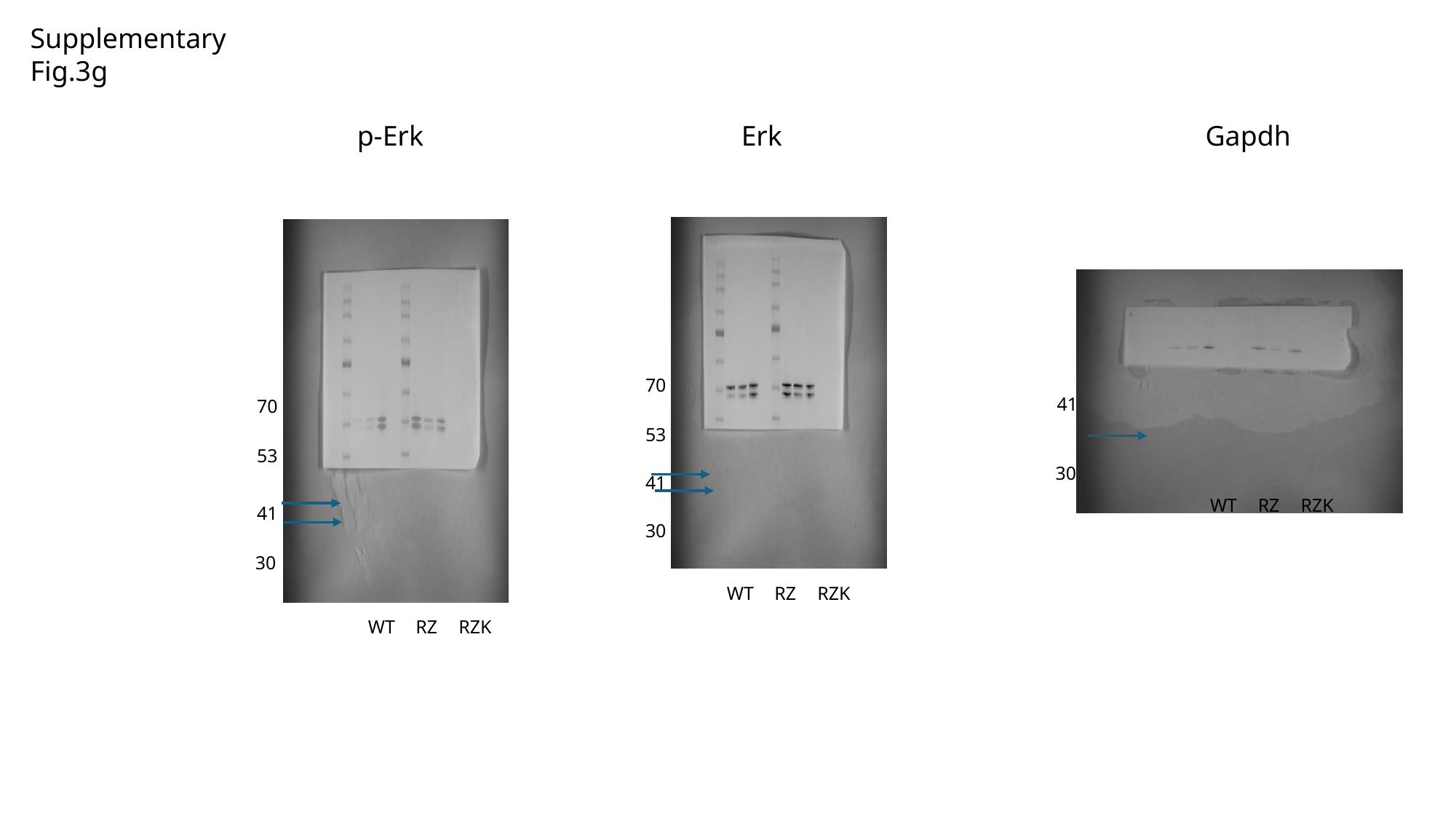

Supplementary Fig.3g
p-Erk
Erk
Gapdh
70
41
70
30
53
53
30
41
RZK
WT
RZ
41
30
30
RZK
WT
RZ
RZK
WT
RZ

## Slide 6
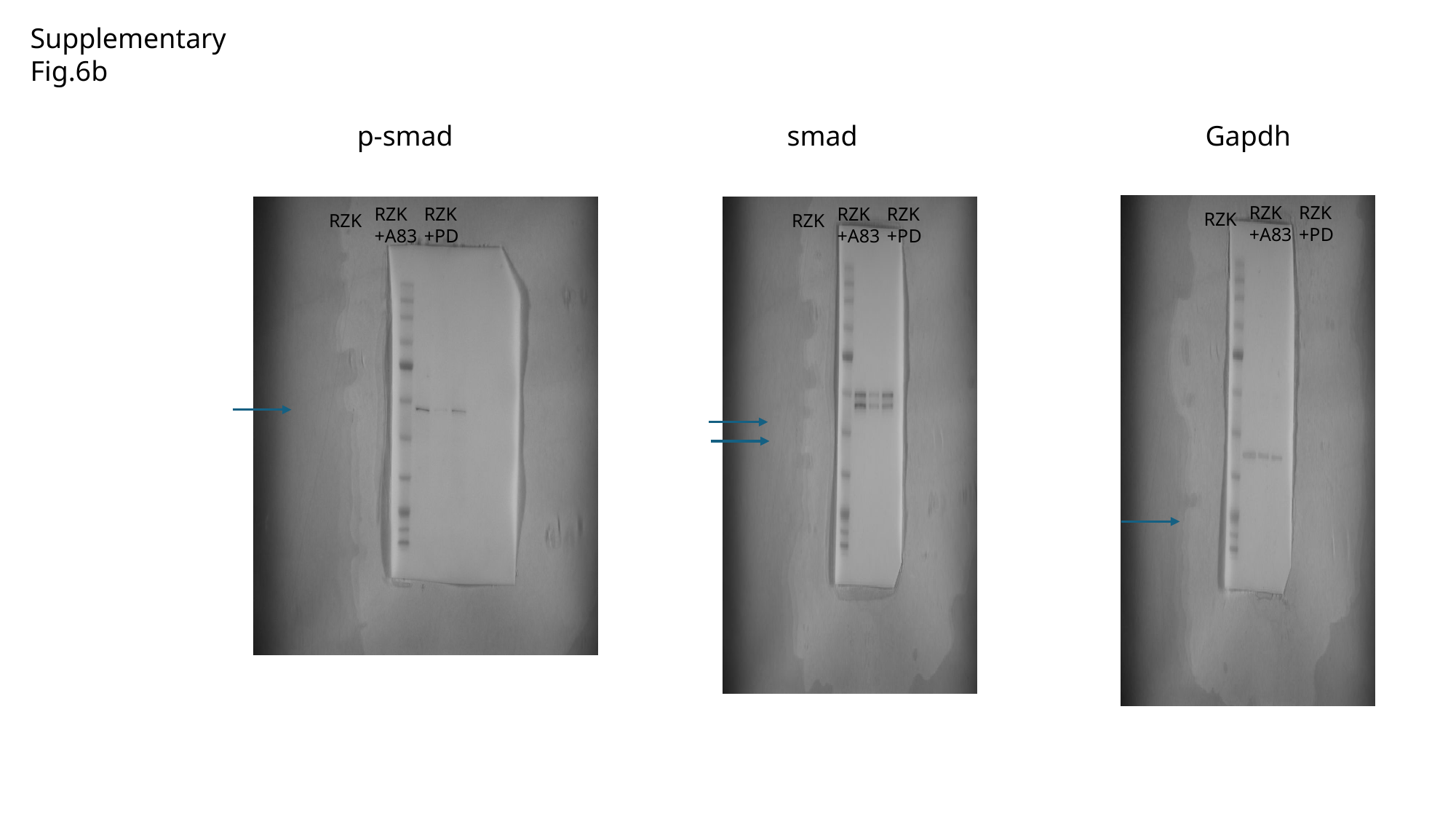

Supplementary Fig.6b
p-smad
smad
Gapdh
RZK +A83
RZK +PD
RZK +A83
RZK +PD
RZK +A83
RZK +PD
RZK
RZK
RZK

## Slide 7
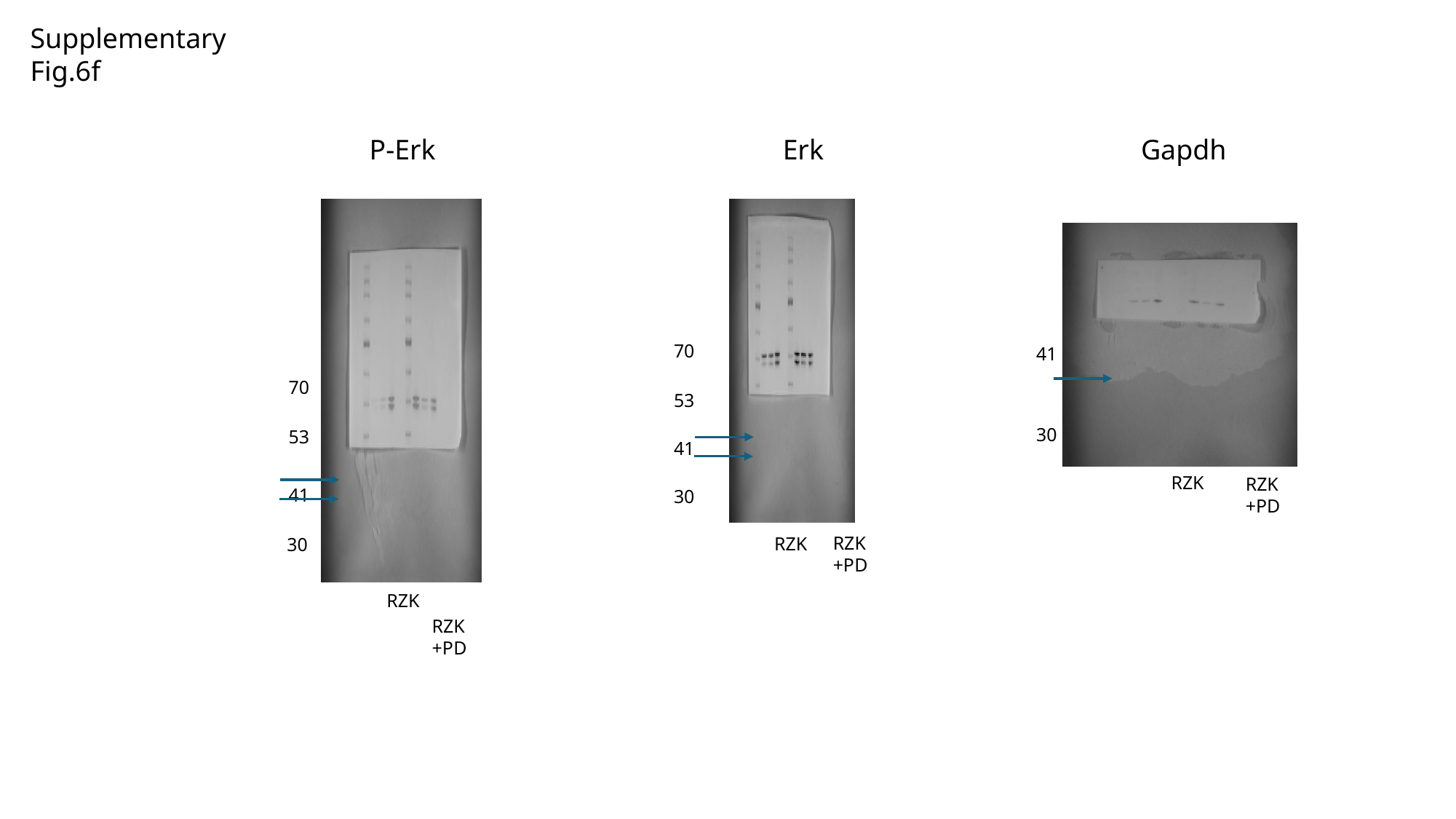

Supplementary Fig.6f
P-Erk
Erk
Gapdh
70
41
70
53
30
53
41
RZK
RZK +PD
41
30
RZK +PD
RZK
30
RZK
RZK +PD
